# Supplementary material for: Protocol for developing core outcome sets for evaluation of psychosocial interventions for children and families with experience or at risk of child maltreatment or domestic abuse
Source: BMJ Open. 2021 Aug 23;11(8):e044431. doi: 10.1136/bmjopen-2020-044431 (PMC8383853; doi:10.1136/bmjopen-2020-044431)
Supplement: Supplementary data [file bmjopen-2020-044431supp001.pdf]

Supplementary Appendices – Rapid Review Protocols

A - Rapid review of systematic reviews of intervention studies

Review question: How are outcomes defined and measured in controlled trials of interventions aiming to improve outcomes of children and families with children exposed to DVA/CM and those aiming to reduce subsequent abusive behaviour by perpetrators of DVA/CM?

a. This includes the definition and measurement of DVA/CM.

This rapid review will be carried out in two steps: firstly searches for systematic reviews (SR) will be carried out, then these reviews will be used to extract individual studies which will be screened for relevance. This process will be carried out in parallel for the DVA and CM literature.

Study inclusion: Peer-reviewed systematic reviews of controlled or quasi experimental comparator intervention studies: with or without randomisation.

The DARE criteria for SRs are at least 4 of the following: reporting of inclusion/exclusion criteria; adequate search; synthesis of included studies; quality assessment of studies; sufficient detail presented (CRD, 1995). For the purposes of this review, SRs will be included if they use an electronic database and have a structured search strategy.

- Published since 2014.
- No restrictions by country. English language only.

- Individual studies must include DVA/CM in one of the following ways:
  - Entry to the intervention is determined by experience, perpetration or identified as at risk of DVA/CM. (Identification of risk is by researchers, practitioners or participants thus we do not have a definition)
  - Subgroup analysis is carried out of participants who have experienced (or are considered to be at risk of) DVA/CM
  - DVA/CM is measured as an exposure (this could be retro or prospectively reported)

Exclusion: Non peer-reviewed studies, qualitative studies, general literature reviews, protocols, case reports, cross-sectional studies, general discussion papers, letters, commentaries, book chapters, conference papers, theses and dissertations.

Population inclusion: children or families with children at risk of experiencing, or experiencing DVA/CM'. This includes unborn children, children (aged 0 to 18 years), designated as victim or witness. For DVA any adult family members who have a parenting role (Early Intervention Foundation, 2014), whether designated as perpetrator, victim, witness or household member.
For CM any adult family members who have a caring role, whether designated as maltreating parent, witness or household member. These adults and children could either be the primary study population of interest or form a subgroup in a wider study population.

Intervention inclusion: Any interventions or services where:

Howarth E, *et al. BMJ Open* 2021; 11:e044431. doi: 10.1136/bmjopen-2020-044431

- Experience of or increased risk of experiencing DVA/CM is a criterion for being offered the service
  OR
- DVA/CM is measured as an exposure or outcome of interest
  AND
- At least one child or family-level outcome is measured. Family-level outcomes do not need to be explicitly labelled as 'family' level, we will make a judgement. However, they include any outcome that affects the family/household unit. For example, worklessness in study where at least some participants are reported to be parents would be included.

- Studies must include evaluation of a defined activity/programme and evaluation of a hypothesised effect
- Interventions may be delivered to any family member(s) as an individual or in a group. Any duration of intervention will be included. Any setting will be considered.

Exclusion: universal interventions that do not specifically target children and families at risk of DVA/CM; targeted interventions that do not measure any child or family level outcomes e.g. perpetrator programmes that focus solely on attitudinal change; DVA (only) interventions focused solely on elder abuse, sibling abuse or child perpetration of domestic violence where participants have not been identified as exposed to DVA.

Comparator inclusion: Any control or comparison group/period with participants receiving no care, treatment as usual or any other treatment.

Outcome inclusion:

- Any child outcome related to i) the child's experience of adversity ii) child functioning, including risky behaviours (see (Maclean et al., 2016) for full list of health and wellbeing outcomes).
- Any outcomes related to the quality of the caregiving environment (e.g. parenting, maternal depression, stressful life events, maternal psychological distress, parental substance misuse).
- Any outcomes related to material deprivation e.g. low income, economic hardship or stress (including perceived), social capital, hunger, food poverty, housing instability.
- Any other outcome judged to relate to children or families by the research team.
- Outcomes can be reported by professionals, child, parent or other family member and they can be retrospective or prospective.
- Outcomes can be end points, surrogate markers for end points or intermediate outcomes.
- No minimum or maximum follow-up is required.

Context inclusion: Studies from any country in any setting.

Searches

The following electronic databases will be searched from 2014: Medline, Embase, PsycInfo, Cochrane and Web of Science. Searching will include expert recommendations of relevant broader studies, including relevant parenting programmes.

The search strategy will include MeSH terms relating to DVA/CM and the BMJ systematic review strategy ((*Study Design Search Filters | BMJ Best Practice*, n.d.)). Key word terms for DVA/CM, abuse,

violence, family members and systematic reviews will be used. These have been developed from the two main NIHR-funded studies in the area ((Howarth et al., 2016) and (Macdonald et al., 2016)) and adapted as required for the different databases with guidance from an expert librarian.

These reviews will be carried out separately for DVA and CM. The DVA search will be run first and any CM studies that do not mention DVA will be excluded (and vice versa). As part of the review involves collecting definitions of DVA/CM, any study deemed to fit within the umbrella by the research team will be included.

Data extraction (selection and coding)
All systematic reviews identified by database searches will be downloaded to CADIMA (Kohl et al., 2018) and de-duplicated. Screening criteria will be tested by two reviewers on 200 titles/abstracts and interrater reliability assessed. Titles/abstracts will be screened by one reviewer for inclusion in full-text review. A second reviewer will independently review 10% of title/abstracts. If there is a high level of disagreement, the second reviewer will continue reviewing titles/abstracts until agreement is reached. Full-text systematic reviews will be screened for inclusion and a second reviewer will independently review 10% of these as above. Key data from the systematic reviews (e.g. definition of DVA/CM) will be extracted into CADIMA by one reviewer.

Individual studies will be extracted from the included full-text systematic reviews. These studies will be downloaded to Zotero and de-duplicated. The remaining studies will then be screened for inclusion in full-text review and data extraction. Data will be extracted into Access using a standardised form and a second researcher will review extraction from the first 5 papers. The following data will be extracted: bibliographic information, study design, setting, sample characteristics, definitions of DVA/CM, intervention details, primary and secondary outcomes (applicable for children and families) and their measures, descriptions of mechanisms. (Where DVA/CM is not measured as an outcome, nor is there a subgroup analysis, only exposure definition will be extracted.) Quality control/risk of bias will not be assessed because the aim of the review is solely to collect outcomes.

Strategy for data synthesis
Narrative synthesis and tabulation of outcomes extracted.

B - Rapid review of qualitative studies

Review questions:

1) What outcomes (benefits or harms) are sought or experienced by actual or potential recipients of interventions/services aiming to prevent or reduce the risk of harm associated with DVA/CM?

2) What outcomes (benefits or harms) are sought by stakeholders* involved in developing and/or delivering interventions to children/families experiencing DVA/CM?

**'stakeholder' is defined as in the IMPROVE study i.e. young people with experience of DVA/CM services, parents/caregivers with experience of using DVA/CM services or professionals involved in commissioning and delivering services to families affected by DVA/CM.*

This review will be carried out in parallel for DVA and CM.

Study inclusion:

- Primary qualitative (i.e. analysis of interviews, focus groups or other verbal analysis which is not quantified) intervention studies either as a standalone study or a discrete component of mixed-method studies.
- Direct and sufficient verbatim text from participants for analysis (i.e. more than two lines) c.f. Arai et al. (2019).
- Published since October 2015 (DVA) and July 2014 (CM) to build on Howarth et al. (2016) and Macdonald et al. (2016).
- No restrictions by country. English language only.

- Individual studies must include DVA/CM in one of the following ways:
    - Participation in the study is determined by experience, perpetration or specifically identified as at risk of DVA/CM. Participants may have received an intervention or may be discussing the impact of DVA/CM and their desired outcomes for the future. (To ensure we are not limited by outcomes defined by current interventions).

  OR
    - Stakeholders involved in developing and/or delivering interventions to children/families experiencing DVA/CM (c.f. Howarth et al, 2016, p.52), or stakeholder discussion of outcomes that are sought either in relation to an intervention or the future in general.

Exclusion: Non peer-reviewed studies, surveys or quantitative studies with descriptive free-text only, general literature reviews, case reports, general discussion papers, letters, commentaries, editorials, book chapters, conference papers, theses and dissertations.

Population inclusion: Any adult or child stakeholders relevant to DVA/CM. This could be as a result of experience, perpetration, identified as at risk, delivering, commissioning or intending to deliver services.

Phenomenon of interest: DVA/CM

Design: Any qualitative approach to data collection and analysis (e.g. interviews, focus groups)

Evaluation: Perspectives of experienced or anticipated benefits or harms of interventions, and/or desired outcomes in general related to DVA/CM.

Searches

The following electronic databases as advised for qualitative research (Evans, 2002; McFadden et al, 2012; Booth, 2016) will be searched from October 2015 (DVA) and July 2014 (CM): ASSIA, CINAHL, GoogleScholar (first 100 hits), PsycInfo and SSCI.

This review is building on Howarth et al. (2016) and Macdonald et al. (2016) so relevant studies from these reviews (and related work such as Arai et al. (2019)) will be included. In addition, expert recommendations of relevant qualitative studies or reviews and any qualitative studies identified from the reviews of systematic reviews will be included.

The search strategy will use the same terms for DVA/CM as the review of systematic reviews, plus additional search terms to identify qualitative research. These will be adapted as required for the different databases with guidance from an expert librarian.
These reviews will be carried out separately for DVA and CM. The DVA search will be run first and any CM studies that do not mention DVA will be excluded (and vice versa) but put aside for inclusion in the relevant review. This review will not adhere to set definitions of DVA/CM, thus any study deemed by the research team to address the phenomena of interest will be included and justified in the discussion of findings.

Screening
Screening of abstracts from the searches and articles included in the full text stage will be guided by questions asked in the IMPROVE study (Howarth et al., 2016):

1) Is this qualitative research?
2) Is there sufficient verbatim text? (i.e. more than 2 lines)
3) Does the paper discuss perspectives of experienced or anticipated benefits or harms of interventions, and/or desired outcomes in general related to DVA/CM.

All articles identified by searches will be downloaded to CADIMA (Kohl et al., 2018) and de-duplicated. Screening criteria will be tested by two reviewers on 10% titles/abstracts and interrater reliability assessed. Titles/abstracts will be screened by one reviewer for inclusion in full-text review. A second reviewer will independently review 10% of title/abstracts. If there is a high level of disagreement, the second reviewer will continue reviewing titles/abstracts until agreement is reached. Full-text systematic reviews will be screened for inclusion and a second reviewer will independently review 10% of these as above. Key details (e.g. bibliographic information, study design, setting, participants etc.) about each full-text inclusion will be recorded in Access.

Strategy for data synthesis
Thematic frameworks will be developed from the IMPROVE study (Howarth et al., 2016) for DVA and the parallel CM study (MacDonald et al., 2016), and input into NVivo 11 (QSR International). The frameworks will focus on barriers and harms of interventions according to parents, children and stakeholders, based on the research questions. These will be used as the basis for a framework analysis (Ritchie & Lewis, 2003) of the studies from the review (Howarth et al., 2016; Arai et al., 2019; Macdonald et al., 2016). As per Howarth et al. (2016), participant quotations and author-identified themes will be extracted rather than line by line coding. Findings will be grouped by whose view was reported and extracts from the texts will be categorised according to this framework with the aim will be to meta-aggregate the studies' findings. Further categories will be developed where there are discrepancies or gaps in the initial framework.

The analysis and interpretation of the findings will occur at the synthesis stage in order to provide an overview of the findings, informed by the principles of meta-synthesis (c.f. Noblit & Hare, 1988), although using a lighter touch given time constraints. Two researchers will work together throughout this process to ensure consistency of categorisation and analysis. Quality will not be assessed because the aim of the review is solely to identify candidate outcomes. The ENTREQ statement (Tong et al., 2012) will be followed for the write-up.

Howarth E, *et al. BMJ Open* 2021; 11:e044431. doi: 10.1136/bmjopen-2020-044431

C – Rapid review of grey literature

Review questions:

1) How are DVA and CM defined in relevant UK service policy contexts?
2) How are outcomes defined: (i) in UK service-based evaluations of interventions? (ii) in relevant policy or commissioning frameworks?

This review will be carried out as a single process given the likelihood of crossover literature. Findings will be recorded as DVA or CM or both.

Literature inclusion:

- Any national or regional policy or practice document that reports on DVA/CM-relevant services or outcomes (e.g. measurement/theory).
- Participation in the service is determined by experience, perpetration or identified as at risk of DVA/CM. (Identification of risk is by practitioners or participants thus we do not have a definition).
- Published since 2016 to build on Howarth et al. (2016) and Macdonald et al. (2016).
- England-based only. English language only.

Exclusion: Publication in academic journals, book chapters, conference papers, theses and dissertations.

Population inclusion: children or families with children at risk of experiencing, or experiencing DVA/CM. This includes unborn children, children (aged 0 to 18 years), designated as victim or witness. For DVA any adult family members who have a caring or parenting role (Early Intervention Foundation, 2014), whether designated as perpetrator, victim, witness or household member. For CM any adult family members who have a caring role, whether designated as perpetrator, witness or household member.

Service inclusion: Any services where:

- Experience of or increased risk of experiencing DVA/CM is a criterion for being offered the service/intervention.
- Services/interventions may be delivered to any family member(s) as an individual or in a group. Any duration of service/intervention will be included. Any setting will be considered.

OR

- Any evaluative work or outcomes framework where at least one child or family-level outcome is evaluated/discussed. Family-level outcomes do not need to be explicitly labelled as 'family' level, we will make a judgement. However, they include any outcome that affects the family/household unit. For example, worklessness in study where at least some participants are reported to be parents would be included.

Exclusion: universal services/interventions that do not specifically target children and families at risk of DVA/CM; targeted services/interventions that do not measure any child or family level outcomes e.g. perpetrator programmes that focus solely on attitudinal change; DVA (only) services/interventions focused solely on elder abuse, sibling abuse or child perpetration of domestic violence, where participants have not been identified as exposed to DVA (i.e. perpetration of abuse by a child could feasibly be an outcome associated with exposure).

Howarth E, *et al. BMJ Open* 2021; 11:e044431. doi: 10.1136/bmjopen-2020-044431

Outcome inclusion: Any family or child-level outcome measured or evaluated or discussed in any way. Intermediate outcomes that could feasibly represent preconditions needed to reach distal/final outcomes (including those relating to the process of service delivery) will be included, along with final/distal outcomes.

Searches

The following databases and websites will be searched:

Grey databases: NICE Evidence Search and Open Grey

Organisation websites including but not limited to:

DVA: Women's Aid, Refuge, Respect, Safe Lives, Voices, AVA, Standing Together, Imkaan, The Stefanou Foundation, Women's Trust, Hestia, DVIP, Nia, The Haven, ManKind Initiative, Everyman Project, NCDV, Galop, LAWA, IDAS, Advance, Your Sanctuary, Advocacy After Fatal Domestic Abuse (AAFDA); Aurora New Dawn; My Sister's Place

CM: Centre of expertise on child sexual abuse, FDAC, SCIE, The Survivors' Trust

General websites: Victim Support, Barnardos, NSPCC, Early Intervention Foundation, NatCen, RCGP, RCN, RCM, NICE, BPS, IHV, WHO, UNICEF, Working together, gov.uk (incls e.g. DA bill, 'Working together'), Public Health for any UK nation, Office of the children's commissioner for any UK nation, Big Lottery, Comic Relief, The Childhood Trust, UK College of Policing, Research in Practice, 'What Works', Joseph Rowntree Foundation, What Works for Children's Social Care.

Websites will be searched manually for relevant documents. It is anticipated there will be an element of snowball searching as relevant organisations will have links to further organisations. Searches will be run simultaneously and then relevant reports assigned to DVA/CM or both. All websites searched will be recorded in Excel/Access along with relevant details about any reports captured. The expert reference group will be consulted about relevant websites to search or reports to include at multiple timepoints.

Data extraction and synthesis

As a range of types of data are anticipated, both the systematic review and the qualitative review protocols will be adapted as necessary to capture and record relevant information. It is likely that there will be non-standardised evaluation measures and interview quotations. Report identification from websites/databases will be carried out by a single researcher and the process transparently recorded. All details regarding evaluation studies and relevant outcomes will be recorded, and where necessary synthesised when the data is qualitative. Access/Excel/NVivo will be used as required to record all steps and ensure a transparent process. A second researcher will cross-check a subset of the reports and the data extracted to ensure consistency and focus on the review questions.

Howarth E, *et al. BMJ Open* 2021; 11:e044431. doi: 10.1136/bmjopen-2020-044431

Bibliography

Arai, L., Heawood, A., Feder, G., Howarth, E., MacMillan, H., Moore, T. H. M., Stanley, N., & Gregory, A. (2019). Hope, Agency, and the Lived Experience of Violence: A Qualitative Systematic Review of Children’s Perspectives on Domestic Violence and Abuse. *Trauma, Violence, & Abuse*, 152483801984958. https://doi.org/10.1177/1524838019849582

CRD. (1995). *Database of Abstracts of Reviews of Effects (DARE): Quality-assessed Reviews*. Centre for Reviews and Dissemination (UK).

Early Intervention Foundation. (2014). *Early Intervention in Domestic Violence and Abuse*.

Howarth, E., Moore, T. H., Welton, N. J., Lewis, N., Stanley, N., MacMillan, H., Shaw, A., Hester, M., Bryden, P., & Feder, G. (2016). IMPRoving Outcomes for children exposed to domestic ViolencE (IMPROVE): An evidence synthesis. *Public Health Research*, *4*(10), 1–342. https://doi.org/10.3310/phr04100

Kohl, C., McIntosh, E. J., Unger, S., Haddaway, N. R., Kecke, S., Schiemann, J., & Wilhelm, R. (2018). Online tools supporting the conduct and reporting of systematic reviews and systematic maps: A case study on CADIMA and review of existing tools. *Environmental Evidence*, *7*(1), 8. https://doi.org/10.1186/s13750-018-0115-5

Macdonald, G., Livingstone, N., Hanratty, J., McCartan, C., Cotmore, R., Cary, M., Glaser, D., Byford, S., Welton, N. J., Bosqui, T., Bowes, L., Audrey, S., Mezey, G., Fisher, H. L., Riches, W., & Churchill, R. (2016). The effectiveness, acceptability and cost-effectiveness of psychosocial interventions for maltreated children and adolescents: An evidence synthesis. *Health Technology Assessment*, *20*(69), 1–508. https://doi.org/10.3310/hta20690

Maclean, M. J., Sims, S., O’Donnell, M., & Gilbert, R. (2016). Out-of-Home Care versus In-home Care for Children Who Have Been Maltreated: A Systematic Review of Health and Wellbeing Outcomes: Systematic Review of Outcomes of Out-of-Home Care. *Child Abuse Review*, *25*(4), 251–272. https://doi.org/10.1002/car.2437

Study design search filters | BMJ Best Practice. (n.d.). Retrieved 16 April 2019, from https://bestpractice.bmj.com/info/us/toolkit/learn-ebm/study-design-search-filters/

Tong, A., Sainsbury, P., & Craig, J. (2007). Consolidated criteria for reporting qualitative research (COREQ): A 32-item checklist for interviews and focus groups. *International Journal for Quality in Health Care*, *19*(6), 349–357. https://doi.org/10.1093/intqhc/mzm042
