# Supplementary material for: Protocol for developing core outcome sets for evaluation of psychosocial interventions for children and families with experience or at risk of child maltreatment or domestic abuse
Source: BMJ Open. 2021 Aug 23;11(8):e044431. doi: 10.1136/bmjopen-2020-044431 (PMC8383853; doi:10.1136/bmjopen-2020-044431)
Supplement: Author's manuscript [file bmjopen-2020-044431.draft_revisions.pdf]

BMJ Open is committed to open peer review. As part of this commitment we make the peer review history of every article we publish publicly available.

When an article is published we post the peer reviewers' comments and the authors' responses online. We also post the versions of the paper that were used during peer review. These are the versions that the peer review comments apply to.

The versions of the paper that follow are the versions that were submitted during the peer review process. They are not the versions of record or the final published versions. They should not be cited or distributed as the published version of this manuscript.

BMJ Open is an open access journal and the full, final, typeset and author-corrected version of record of the manuscript is available on our site with no access controls, subscription charges or pay-per-view fees (http://bmjopen.bmj.com).

If you have any questions on BMJ Open's open peer review process please email info.bmjopen@bmj.com

BMJ Open

Protocol for developing Core Outcome Sets for evaluation of psychosocial interventions for children and families with experience or at risk of child maltreatment or domestic abuse

Journal:	*BMJ Open*
Manuscript ID	bmjopen-2020-044431
Article Type:	Protocol
Date Submitted by the Author:	03-Sep-2020

Complete List of Authors:	Howarth, Emma; University of East London; University College London, Institute of Child Health<br>Powell, Claire; University College London, Institute of Child Health<br>Woodman, Jenny; University College London, Institute of Education<br>Walker, Erin; University College London, UCL Partners<br>Chesters, Heather; University College London, Institute of Child Health<br>Gilbert, Ruth; UCL Institute of Child Health, Centre for Paediatric Epidemiology and Biostatistics<br>Feder, Gene; University of Bristol, Community based medicine

Keywords:	Child protection < PAEDIATRICS, Community child health < PAEDIATRICS, PUBLIC HEALTH, SOCIAL MEDICINE

SCHOLARONE™
Manuscripts

I, the Submitting Author has the right to grant and does grant on behalf of all authors of the Work (as defined in the below author licence), an exclusive licence and/or a non-exclusive licence for contributions from authors who are: i) UK Crown employees; ii) where BMJ has agreed a CC-BY licence shall apply, and/or iii) in accordance with the terms applicable for US Federal Government officers or employees acting as part of their official duties; on a worldwide, perpetual, irrevocable, royalty-free basis to BMJ Publishing Group Ltd ("BMJ") its licensees and where the relevant Journal is co-owned by BMJ to the co-owners of the Journal, to publish the Work in this journal and any other BMJ products and to exploit all rights, as set out in our licence.

The Submitting Author accepts and understands that any supply made under these terms is made by BMJ to the Submitting Author unless you are acting as an employee on behalf of your employer or a postgraduate student of an affiliated institution which is paying any applicable article publishing charge ("APC") for Open Access articles. Where the Submitting Author wishes to make the Work available on an Open Access basis (and intends to pay the relevant APC), the terms of reuse of such Open Access shall be governed by a Creative Commons licence – details of these licences and which Creative Commons licence will apply to this Work are set out in our licence referred to above.

Other than as permitted in any relevant BMJ Author's Self Archiving Policies, I confirm this Work has not been accepted for publication elsewhere, is not being considered for publication elsewhere and does not duplicate material already published. I confirm all authors consent to publication of this Work and authorise the granting of this licence.

For peer review only

TITLE **Protocol for developing Core Outcome Sets for evaluation of psychosocial interventions for children and families with experience or at risk of child maltreatment or domestic abuse**

Emma Howarth (Corresponding author)
School of Psychology, UEL
University Way, London, UK E16 2RD
Email: e.howarth@uel.ac.uk

Claire Powell
Institute of Child Health, UCL
30 Guilford St, London, UK WC1N 1EH

Jenny Woodman
Institute of Education, UCL
27/8 Woburn Square, London, UK WC1H 0AA

Erin Walker
UCL Partners
170 Tottenham Court Road, London, UK W1T 7HA

Ruth Gilbert
Institute of Child Health, UCL
30 Guilford St, London, UK WC1N 1EH
United Kingdom

Gene Feder
School of Social and Community Medicine, University of Bristol
39 Whatley Road, Bristol BS8 2PS
United Kingdom

KEY WORDS Core outcome set; intervention; child maltreatment; domestic abuse; evaluation

WORD COUNT 4717

For peer review only - http://bmjopen.bmj.com/site/about/guidelines.xhtml

ABSTRACT

Introduction

Widespread recognition that child maltreatment (CM) and domestic violence and abuse (DVA) are common and have serious and long-term adverse health consequences, has resulted in policies and programmes to ensure that services respond to and safeguard children and their families. However, high quality evidence about how services can *effectively* intervene is scant. The value of the current evidence base is limited partly because of the variety of outcomes and measures used in evaluative studies. One way of addressing this limitation is to develop a Core Outcome Set (COS) which is measured and reported as a minimum standard in the context of trials and other types of evaluative research. The study described in this protocol aims to develop two discrete core outcome sets for use in future evaluation of psycho-social interventions aimed at improving outcomes for children and families at risk or with experience of i) CM; or ii) DVA.

Methods and analysis

We will use a two-phase mixed methods study design. Phase 1 (outcome generation) will include rapid reviews of evidence (trials, qualitative, grey), stakeholder workshops and semi-structured interviews with adult survivors of CM/DVA and parents. Phase 2 (outcome identification) will include a three panel (professionals, researchers, survivors) adapted eDelphi study and consensus meeting. This study protocol adheres to reporting guidance for core outcome set protocols and has been registered on the COMET database (http://www.comet-initiative.org/Studies/Details/1576).

Ethics and dissemination

We will disseminate our findings through peer reviewed and open access publications, the COMET website, and presentations at international conferences. We will engage with research networks, journal editors and funding agencies to promote awareness of the CM- and DVA-COS. We will work with advisory and survivor and public involvement groups to co-produce a range of survivor, policy and practice facing outputs.

Ethical approval for this study has been granted by the Research Ethics Committee at University College London.

STRENGTHS AND LIMITATIONS OF THIS STUDY

- To our knowledge this is the first attempt to develop core outcome sets to address family violence and abuse.

For peer review only - http://bmjopen.bmj.com/site/about/guidelines.xhtml

- The study draws on diverse evidence sources and includes people with lived experience, practitioners and policy makers, as well as researchers.
- This study provides the opportunity to consider the overlap in outcomes sought across two different but related exposures,
- This study is limited by the lack of direct involvement of children and young people
- It is also beyond the means of the study to involve survivors and service providers from low- and middle-income countries (LMICs), although we will include research from LMICs in the evidence reviews and actively recruit researchers from or researching LMIC settings.

For peer review only

For peer review only - http://bmjopen.bmj.com/site/about/guidelines.xhtml

INTRODUCTION

Widespread recognition that child maltreatment (CM) and domestic violence and abuse (DVA) are common and have serious and long-term adverse health consequences, [1,2] has resulted in policies and programmes to ensure that services respond to and safeguard children (and their families) at high risk of, or with experience of CM and or/DVA.[3–6] However, high quality evidence about how services can *effectively* intervene is scant. [7–9]

The value of the current evidence base is limited partly because of the variety of outcomes and measures used in evaluative studies. [7,8]. This hampers the ability to aggregate evidence pertaining to one particular type of intervention, so as to build a comprehensive picture of its effectiveness when delivered to different populations or in different contexts. Similarly it is challenging to make comparisons between different types of interventions, which purport to address the same problem within the same group of individuals [10,11].

More fundamentally, outcomes measured in CM and DVA intervention studies are often a poor or partial reflection of the concepts of success held by those that use, deliver and pay for interventions [7,8,12]. The ultimate goal of intervention studies is to identify interventions that can benefit individuals, families and communities in the future. Therefore, it is crucial that they measure outcomes reflecting the priorities and expectations of these groups so the evidence they generate is relevant to consumers. Outcomes also need to resonate with the priorities of policymakers and service providers, else effective interventions may be overlooked by those responsible for funding and/or delivery decisions, and never commissioned or implemented[13,14].

Together, these issues mean it is difficult to extract the information needed to inform real world decisions about which CM/DVA interventions to commission and scale, and which to stop funding.

One way of addressing the limitations set out above is to develop a Core Outcome Set (COS), a standardised set of outcomes that researchers, providers, service users and commissioners consider critical or important outcomes in the management of a condition or in this case, a complex public health challenge[11,15]. The COS is then measured and reported, as a minimum standard in the context of trials or other types of research and evaluation [15] and sometimes practice-based monitoring. [16] The aim is to enhance the methodological standard and utility of research in the field, by increasing consistency and reducing reporting bias (where many outcomes are measured and only favourable effects reported), and to ensure that the views of important constituencies influence the selection of outcomes to be included in the COS [10].

For peer review only - http://bmjopen.bmj.com/site/about/guidelines.xhtml

The idea of the COS as a mechanism for improving evidence quality has gathered momentum over the past decade since the establishment of the COMET (Core Outcome Measures for Effectiveness Trials) initiative in 2010 (www.comet-initiative.org) [15]. Whilst the number of core outcome sets being developed has increased steadily since [16,17], it is clear that in the main, studies have focused on COS development for specific health conditions, pharmacological or surgical interventions and/or discrete interventions delivered in health care settings [16,17]. In contrast there has been relatively less focus on the development of COS in relation to public health problems that require complex multi-sectoral responses often delivered to whole families, or multiple members of the same family.

Current study

The study aims to develop two discrete core outcome sets for use in future evaluation of psycho-social interventions aimed at improving outcomes for children and families at risk of or with experience of CM or DVA. Children's experiences of CM and DVA frequently overlap [18] and experience of DVA is now often conceived of as a type of maltreatment in its own right, or a feature of emotional maltreatment. [19,20] Nevertheless, the response to these two types of trauma can be different, at least in the UK. This provides the rationale for developing separate outcome sets, however we will explore where the derived outcome sets overlap with a view to identifying outcomes that can be measured in family contexts where *both* CM and DVA occur. This is a move away from a focus on single problem areas towards recognition of the constellation of risks often experienced by children and their families.

METHODS AND ANALYSIS

This study protocol adheres to reporting guidance for core outcome set protocols [21] and has been registered on the COMET database.

Scope of outcome sets

The CM-COS and the DVA-COS will be developed with the aim of evaluating the impact of targeted child and/or family focussed psychosocial interventions or services in the context of both research (randomised and non-randomised studies) and practice (service evaluations and monitoring). The target population is children aged less than 19 years of age with experience of (current or previous) DVA or CM. Given that many interventions aiming to improve child outcomes do so via support delivered to parents or multiple family members (rather than directly to the child), [7,8,22] the target group also includes parents or families of children experiencing CM or DVA. We use a definition of Psychosocial interventions set out by the Institute of Medicine. [23]

For peer review only

For peer review only - http://bmjopen.bmj.com/site/about/guidelines.xhtml

Interventions within the scope of this study include psychotherapies (e.g. cognitive-behavioural therapy), community-based treatments, family/systemic therapy, vocational rehabilitation, peer support services, integrated care interventions, and out-of-home care (i.e. foster care or adoption). Interventions may be delivered in one or more contexts, for example clinic, school, community. Interventions may be individual, dyad or group based, or a combination, and delivered to children with or without their parents, to parents alone, to family groups, or some combination. To be in scope an intervention must implicitly or explicitly aim to improve child outcomes by one of the following mechanisms: i) reducing the risk of CM/DVA occurring/reoccurring in the family; ii) improving parental functioning; iii) limiting or preventing poor mental health, reduced wellbeing or function in children; iv) promoting children's recovery following experience of CM or DVA – in this instance we relate to the recovery model definition which emphasises perceptions of resilience, self-identity, a sense of empowerment, hope and optimism (e.g. [24]) Universal and targeted structural interventions are not in scope.

Study design

The study will be undertaken in two stages (see Figure 1). The first stage will seek to identify candidate outcome areas, domains and indicators. Multiple methods will be used to identify items for the candidate list including rapid evidence reviews, consultation with key stakeholders and qualitative interviews. Data will be synthesised to produce a taxonomy of outcomes, from which the two candidate lists will be produced.

The second stage will incorporate an adapted two round eDelphi study and consensus meeting, with the aim of building agreement between different stakeholder groups regarding important outcomes. This method has been used extensively in the context of core outcomes research [16,25,26].

We will recruit three panels to ensure that each stakeholder group is equally represented in the final consensus [27]. In a further effort to ensure the views of those with lived experience remain central during this exercise, the eDelphi method will be adapted so, in addition to feedback about their individual and own panel scores for each item, professional and researcher panels will also receive feedback about the scores of the lived experience panel. This adaptation is informed by evidence that feedback of patient scores to clinicians results in an expanded set of consensus items that better reflect the priorities of patients. [28] Additional feedback will not be given to the lived experience panel, so as to minimise the possibility of perceived power differentials influencing this panel's ratings [27]. A final face to face consensus meeting will be used to review and verify findings from the Delphi study, clarify any remaining uncertainty, and ratify the final core outcome set.

[Figure 1 about here]

For peer review only

Study Oversight

A steering committee including practitioners, policy makers and researchers representing CM and DVA will be formed and will meet formally twice a year. Three public advisory groups will also oversee and consult on the study. One group will be comprised of individuals with lived experience of DVA and one of care experienced young people. These groups will be formed in partnership with relevant survivor led organisations. A third group will be comprised of young people affiliated to the National Children's Bureau who are consulting more broadly on the work of the Children and Families Policy Research Unit. Partner organisations will be funded to organise three meetings per year and to provide appropriate remuneration to participants. Additional funds will be paid to cover scheduled review activities organised with partner agencies via email.

Participants

Workshops (Phase 1*)*: We will invite 30-40 individuals to attend each workshop, the aim of which will be to discuss definitions of CM/DVA and outcomes perceived to be important for survivors. Relevant researchers (mainly UK) and professionals from each field (e.g. support workers, primary and secondary health practitioners, education staff, local authority commissioners, local and national policy makers) will be identified from the research team's networks, authorship of key publications, and internet searches.

People with lived experience of CM/DVA will be approached via gatekeeper organisations and existing survivor/researcher networks known to the research team. Concerted effort will be made to invite individuals representing groups known to be marginalised from services or research on DVA/CM, or who receive inadequate service responses owing to discrimination or lack of service differentiation (i.e. assuming all groups require the same response). [29–32]

Semi-structured interviews (Phase 1*)*: We will recruit a sample of approximately 5 adults who identify as survivors of CM or DVA, and 5 parents of children currently aged 0-18 with lived experience of DVA/CM. In the first instance we will seek to recruit participants via gatekeeper organisations (see procedure below), although if recruitment is insufficient, we will seek approval for direct recruitment via social and print media. To take part in interviews, participants will be required to self-identify as having experienced CM/DVA, or as having a child who has experienced CM/DVA.

Adapted international eDelphi study (Phase 2): Three separate panels will be recruited to take part in the eDelphi study comprising: i) individuals with lived experience (parents and adults exposed to abuse in childhood); ii) frontline and strategic professionals involved in the delivery of CM/DVA services; iii) researchers. The first two panels will include members from the UK, with the researcher

For peer review only - http://bmjopen.bmj.com/site/about/guidelines.xhtml

panel including international researchers from high, middle- and low-income countries. We will aim to recruit 30 individuals to each panel.

Survivors and professionals taking part in the workshops and semi-structured interviews (and who give consent for further contact) will be approached for participation in the lived experience and professional panels respectively. Key researchers, with at least one peer-reviewed publication from either the CM/DVA field, will be identified through the rapid reviews, researcher networks, participation in workshops and via the expert panel.

If needed, additional participants will be recruited through key organisations working with either CM or DVA survivors and snowball sampling. For all panels, participants must be able to read and understand English in order to participate.

Consensus workshop following eDelphi study (Phase 2): A face-to-face consensus meeting, with a purposively sampled panel (n=30) representing all key stakeholder groups, will be recruited from participants taking part in earlier phases of the study. Individuals outside of the study will be approached as needed to ensure balanced representation and inclusion of individuals of strategic importance to take up and implementation of study findings.

Procedure

Phase 1

Rapid reviews

We will conduct a series of rapid reviews using systematic methods (see supplementary file for protocols and review questions). We will review experimental and quasi-experimental intervention studies (international), qualitative studies containing primary accounts of experience of relevant interventions or outcomes that are sought by families experiencing CM/DVA (international), and grey (UK) literature reporting descriptions of interventions, service evaluations or consultation regarding appropriate outcomes across the DVA and CM fields.

We will search a range of relevant databases and websites under the guidance of an expert librarian. Following rapid review techniques [33,34] we will search since 2014 for intervention studies (covering the time elapsed since previous key reviews, [8,35]) and 2014/5 for the qualitative studies to build on recent qualitative reviews [36]. The grey literature review will primarily focus on searches of relevant UK organisation websites and will include any service or intervention evaluation or any

For peer review only

For peer review only - http://bmjopen.bmj.com/site/about/guidelines.xhtml

consultation or review, to identify relevant outcomes or outcome tools for use in the context of service delivery or evaluation.

A second reviewer will screen and extract data from a minimum of 5% of titles/abstracts and articles to ensure consistency. Inter-rater reliability kappa scores will be calculated, and disagreements resolved through discussion (or a third reviewer if necessary) throughout the process. Relevant outcome indicators will be extracted, as well as their measurement instruments where possible. There will be no appraisal of study quality and outcomes will be extracted from all identified papers.

Stakeholder workshops

We will hold two invite-only workshops (one focussed on CM and one focussed on DVA) to gather stakeholder views. The purpose of these events will be to i) explore definitional issues, specifically how each phenomenon is defined by particular groups and the function that this definition plays in practice (in terms of enabling access to services/interventions and measuring change), and ii) to explore outcomes perceived to be important indicators of benefit or harm for children and families experiencing CM/DVA.

Participants will be welcomed to the event and the aims of the event and the broader project discussed; participants will be reassured that all information shared in the group setting will be kept confidential. Participants will be asked to generate ideas relating to desirable (or undesirable) outcomes, unconstrained by what they believe to be measurable or achieved via currently available interventions. This will be an attempt to ensure output is not merely reflective of current practice. Designated scribes will take notes throughout the day, which will be collated and analysed thematically [37]. Participants in the workshops will be asked for permission to contact them at a later date, for the purpose of inviting them to participate in the international eDelphi study.

Interviews with individuals with lived experience of DVA/CM as a child or as parent of a child

Participants will be identified via key gatekeeper organisations (where work with survivors of CM/DVA is core business) which are contacted for the purpose of workshop participation (see above). Participants will be approached directly by a professional from the gatekeep organisation or they will receive an open invitation circulated through the organisation's survivor network. Where participants are approached by professionals, they will be given brief information about the study and asked for permission to pass contact details to the research team. Individuals responding to an open invitation will be asked to contact a member of the research team directly. They will be assured of the anonymity in their involvement.

For peer review only - http://bmjopen.bmj.com/site/about/guidelines.xhtml

For peer review only

Basic socio-demographic information and minimal information about experiences of CM or DVA will be collected via questionnaire prior to the interview and will be used for descriptive purposes. Participants will have the opportunity to take part in the interview face-to-face, by video call or by phone, according to their personal preferences and public health guidance on social distancing. For those participants who wish to take part but are unable to speak directly to interviewers, they will be able to answer the interview questions by email. [38] Interview schedules will be used to guide interviews, which will be recorded and transcribed verbatim and analysed thematically. [37]

Outcome generation

A list of candidate outcome areas (e.g. health and wellbeing), domains (e.g. mental health) and indicators (e.g. withdrawal from friends and activities) will be generated iteratively by the research team, drawing on all information sources described above. An unedited candidate list of outcome indicators generated from stakeholder workshops will be used as a starting point. Identification of duplicate and overlapping outcome indicators from the list will be undertaken in parallel by two team members (CP, EH). Similar items will be dropped or combined to produce a reduced inventory. Disagreements between team members will be resolved through discussion. All suggestions to drop or combine items will be reviewed by two further research team members (RG, GF) and survivor involvement groups. Similar indicators (i.e., outcomes that could be compared across studies or combined in a meta-analysis [21]) will be grouped into outcome domains by two team members, and reviewed by two further members of the research team and survivor involvement groups. Simultaneously, a taxonomy to organise domains into outcome areas will be developed. Here we will draw on existing practical and theoretical frameworks to categorise health outcomes, [39] as well as the aetiology and impacts of DVA and CM [40–43] This overarching framework to describe the nested structure of outcomes identified in workshops will be reviewed and refined by all members of the research team, the expert advisory group, and survivor involvement groups.

A candidate list of outcome indicators from the rapid reviews will be generated and de-duplicated (CP, EH). Four research team members and at least two survivor representatives will, in parallel, attempt to categorise indicators using the developed taxonomy. Categorisations will be compared, disagreements discussed, and consensus reached through discussion. New domains or areas will be added where necessary. Unique indicators (not already included) will be identified from the candidate list generated from the reviews and added to the taxonomy. This iterative process will be repeated with data yielded from interviews.

The final taxonomy and labelling of terms will be reviewed by the advisory group, and all three public involvement groups. Particular attention will be given to the language used to describe

For peer review only - http://bmjopen.bmj.com/site/about/guidelines.xhtml

outcome domains, areas and specific indicators to ensure they are understandable, meaningful and acceptable to all stakeholder groups. Further refinement (including addition of areas, domains or indicators) will be undertaken following this review. The final step in the process will be to examine outcomes against a priori criteria designed to ensure the final CoS has maximum utility. These include: i) the extent to which the outcome indicator relates to children's feelings, function or survival, or the process of delivering services to survivors ii) whether the outcome is 'changeable' iii) and whether the outcome indicator could feasibly change as a result of a psychosocial intervention. Four members of the research team, at least two members of the expert advisory group, and four members of the survivor involvement groups (with equal representation of CM and DVA experience) will independently assess outcome indicators against the criteria listed above. Any indicators identified as not meet all criteria by one or more reviewers will be discussed and a majority decision taken to exclude or include it in the candidate list. Excluded outcomes will be reported in the final paper, along with reasons for exclusion. Where needed a glossary of terms and explanatory text will be developed to aid clarity for participants in the eDelphi study.

Phase 2

Adapted international e-Delphi study

A sequential two-round, three-panel eDelphi study will be conducted.

Round 1*:* A questionnaire for use in the eDelphi study will be developed using the taxonomy described above. Areas and domains will serve as headings and sub-headings to organise the survey, to encourage completion, and to allow us to explore the relative importance of indicators within the same domain. The questionnaire will be reviewed by advisory and involvement groups and refined in line with feedback. Ethical approval will be sought as an amendment to that granted for phase one of the study.

Participants will be contacted by email to remind them about the COS study and their attendance at a previous workshop (if appropriate) and to invite them to participate in the eDelphi study. A second email containing the information sheet and link to an online questionnaire will be sent 1-2 days after the initial contact. Participants will be required to indicate that they have read the information sheet and agree to take part, before proceeding to the questionnaire. The questionnaire will be administered via REDcap (https://www.**project-redcap**.org) hosted by the University College London.

Participants will be presented with a list of outcome indicators organised by area and outcome domain. They will be asked to rate each outcome presented, on a 9-point scale of importance (1=not at all important, 9=extremely important). Participants will also be given the opportunity to add any

For peer review only - http://bmjopen.bmj.com/site/about/guidelines.xhtml

additional outcomes that are missing from each domain. During this round we will also collect demographic data including ethnicity, age, gender, and profession and nationality where appropriate. The questionnaire will remain open for 14 days and reminder emails will be sent out seven and two working days before closure.

Item level descriptive statistics will be generated for each panel including: number of respondents, minimum and maximum values, measures of central tendency and dispersion. Criteria for item inclusion in round 2 will be that an item is rated 7-9 (on a 9-point Likert scale) by 50% or more participants in at least one panel and 1-3 by no more than 15% of participants in any stakeholder group. [44]This low threshold for inclusion enables us to reduce response burden in round two by dropping unimportant items (higher number of items are associated with significantly lower response rates in COS Delphi surveys [45]), while also reducing the likelihood of dropping outcomes that may have been rated more highly in subsequent rounds had participants been given feedback on them. New items will be included if two or more panellists suggested inclusion, and the research team deem it unique to existing content. [15]Panellists completing Round 1 will be invited to participate in Round 2 if they rated ≥75% of survey items. Non-completers will not be contacted for participation in round 2. We will assess attrition rates for each panel and by demographic and abuse profiles.

Round 2*:* An amendment to the existing approval will be sought for use of the shorter round 2 questionnaire. The same items will be included in questionnaires issued to each panel. Each panel member will receive a personalised questionnaire reporting panel averages and their own rating for each item. As noted above, professional and researcher panels will also receive feedback about the ratings of the survivor panel. Panellists will be asked to rerate each of the included items, and rate for the first time, any new outcomes put forward in round 1. All new outcomes suggested in round 1 (irrespective of the panel from which they derived) will be presented to each of the three panels.

As before, participants will receive two reminders to complete the questionnaire, over the course of 14 days. Following completion of the study descriptive statistics will be computed. Items will be deemed important to a particular stakeholder group if they are rated 7 -9 by ≥70% of respondents and 1 - 3 ≤15% by the panel. Conversely, items will be classified as unimportant to a group if ≥70% of respondents rate it as 1-3 and ≤15% rate it as 7-9. Any items not classified as important or unimportant will be deemed not to have reached consensus. Items will be considered 'core' and recommended for inclusion in the outcome set if they are rated as important by all three panels. We will assess the impact of attrition on consensus by comparing (within panels) the mean total item

For peer review only - http://bmjopen.bmj.com/site/about/guidelines.xhtml

scores for those completing round 1 only and those completing both rounds; we will also compare the average scores for completers vs non completers by each outcome (within panel).[15]

Consensus meeting

A face-to-face consensus meeting, with a purposively sampled panel (n=30) representing all key stakeholder groups, will be held to discuss, vote and agree on the final CM- and DVA COS. The format of the meeting will follow the process set out by the James Lind Alliance (JLA) final priority setting workshops http://www.jla.nihr.ac.uk/jla-guidebook/chapter-8/workshop-process-on-the-day.htm This method is pertinent given that JLA priority setting meetings involve multiple stakeholders, discussion of interim results derived from the ranking of evidence uncertainties, and production of a 'top ten'.

Whilst there is no recommended maximum number of outcomes that should be included in a CoS, for it to be pragmatic we aim to arrive at a maximum of 10 outcomes. [46,47] The JLA priority setting method involves a structured process including small group and whole group discussion, ranking and reranking. The method will be adapted to include a preliminary step, where participants review those outcomes identified as important to the lived experience panel, but which didn't reach consensus across all groups. Participants will be asked to identify any outcomes that should be discussed in the workshop alongside outcomes meeting the consensus definition. This initial step is an attempt to ensure appropriate weight is given to the voice of those with lived experience of DVA/CM. During discussion, workshop participants will be asked to take into consideration the extent to which identified outcomes are 'changeable', and could be feasibly impacted by psycho-social interventions. The final CoS and also a list of all items reaching consensus will be published.

ETHICS AND DISSEMINATION

Ethical approval

Ethical approval was sought from the Research Ethics committee at University College London. At all stages of the study we will obtain written consent for contact information relating to potential participants to be passed via gatekeeper organisations assisting with recruitment. We will obtain written informed consent from participants in interviews and consensus meeting. Written informed consent will be obtained from participants when they opt in to participate in the eDelphi study, before they are able to proceed.

Dissemination and implementation

For peer review only - http://bmjopen.bmj.com/site/about/guidelines.xhtml

We have registered the study on the COMET website. We will provide tailored briefings to UK policy makers, think tanks, commissioners and third sector organisations whilst the study is in progress. This will maximise interest and intention to use the core outcome sets. We will involve the leads of international scholarly networks in workshops and recruit member networks to the eDelphi study

We will disseminate our findings through peer reviewed and open access publications, the COMET website, and presentations at international conferences. We will engage with journal editors and funding agencies and the relevant Cochrane and Campbell review groups to promote awareness of the CM- and DVA-COS. We will provide briefings and links to publications to international research and policy networks, as well as those for dissemination through the networks of the VAMHN membership and CPRU collaborators, as well as the wider network of NIHR Policy Research Units, Applied Research Collaborations (ARCs) and UKRI networks. We will invite survivors who participated in workshops and in involvement groups to co-produce plain-language, service-user facing communication materials for circulation in places where survivors access support (formal or informal). We will also develop tailored briefings to enable findings to be shared with all study participants. These will be published on the CPRU website and emailed to all third sector organisations working specifically with survivors of CM and DVA, as well as local authority commissioners and CCGs.

A review of the reach and uptake of the core outcome sets will be undertaken in 2023. One of the key issues for review will be whether the core outcome set as become aligned or adopted by research and practice networks or collaborations to ensure update and sustainability.

DISCUSSION

Currently no published COS exists for evaluation of services and interventions to improve child outcomes following experience of CM or DVA. It is essential that outcomes measured in the context of trials and service monitoring reflect the benefits (and harms) prioritised by those who use, deliver and commission DVA and CM programmes, as well as those who research them. A COS that is developed with strong participation from people with lived experience of CM or DVA and those working to support them will help to ensure that relevant outcomes are measured in all evaluative studies. This in turn will enhance consistency across studies and the quality and value of research. Good awareness and uptake of this study’s outputs is critical to achieving its ultimate aim.

Limitations

The design of this study is limited by the lack of direct involvement of children and young people in either qualitative interviews or the eDelphi study. Given the study described here represents meta-

For peer review only - http://bmjopen.bmj.com/site/about/guidelines.xhtml

research, it was felt that potential risks to children could not be justified. Their voices are nonetheless to some extent reflected through the broad reviews of evidence and inclusion of parent perspectives. It is also beyond the means of the study to involve survivors and service providers from LMICs, although we will include research from LMICs in the evidence reviews and actively recruit researchers from or researching LMIC settings.

For peer review only

For peer review only - http://bmjopen.bmj.com/site/about/guidelines.xhtml

REFERENCES

1 Evans SE, Davies C, DiLillo D. Exposure to domestic violence: A meta-analysis of child and adolescent outcomes. *Aggress Violent Behav* 2008;**13**:131–40.

2 Gilbert R, Widom CS, Browne K, *et al.* Burden and consequences of child maltreatment in high-income countries. Lancet. 2009;**373**:68–81. doi:10.1016/S0140-6736(08)61706-7

3 MacMillan HL, Wathen CN, Barlow J, *et al.* Interventions to prevent child maltreatment and associated impairment. *Lancet* 2009;**373**:250–66. doi:10.1016/S0140-6736(08)61708-0

4 McTavish JR, Kimber M, Devries K, *et al.* Children's and caregivers' perspectives about mandatory reporting of child maltreatment: a meta-synthesis of qualitative studies. *BMJ Open* 2019;**9**:e025741. doi:10.1136/bmjopen-2018-025741

5 Lewis N V, Feder GS, Howarth E, *et al.* Identification and initial response to children's exposure to intimate partner violence: a qualitative synthesis of the perspectives of children, mothers and professionals. *BMJ Open* 2018;**8**:e019761. doi:10.1136/bmjopen-2017-019761

6 Home Office. Domestic Abuse Bill 2020: overarching factsheet. London: 2020. https://www.gov.uk/government/publications/domestic-abuse-bill-2020-factsheets/domestic-abuse-bill-2020-overarching-factsheet

7 Howarth E, Moore THM, Welton NJ, *et al.* Improving outcomes for children exposed to domestic violence (IMPROVE): An evidence synthesis. *Public Heal Res* 2016.

8 Macdonald G, Livingstone N, Hanratty J, *et al.* The effectiveness, acceptability and cost-effectiveness of psychosocial interventions for maltreated children and adolescents: An evidence synthesis. *Health Technol Assess (Rockv)* 2016;**20**. doi:10.3310/hta20690

9 Latzman NE, Casanueva C, Brinton J, *et al.* The promotion of well-being among children exposed to intimate partner violence: A systematic review of interventions. *Campbell Syst Rev* 2019;**15**. doi:10.1002/cl2.1049

10 Clarke M. Standardising outcomes for clinical trials and systematic reviews. *Trials* 2007;**8**:1–3. doi:10.1186/1745-6215-8-39

11 Clarke M, Williamson PR. Core outcome sets and systematic reviews. *Syst Rev* 2016;**5**:11. doi:10.1186/s13643-016-0188-6

12 Howarth E, Moore THM, Shaw ARG, *et al.* The Effectiveness of Targeted Interventions for Children Exposed to Domestic Violence: Measuring Success in Ways that Matter to Children,

For peer review only - http://bmjopen.bmj.com/site/about/guidelines.xhtml

Parents and Professionals. *Child Abus Rev* 2015;**24**:297–310. doi:10.1002/car.2408

13 Hoagwood KE, Jensen PS, Acri MC, *et al.* Outcome domains in child mental health research since 1996: have they changed and why does it matter? *J Am Acad Child Adolesc Psychiatry* 2012;**51**:1241-1260.e2. doi:10.1016/j.jaac.2012.09.004

14 Howarth E, Moore THM, Shaw ARG, *et al.* The Effectiveness of Targeted Interventions for Children Exposed to Domestic Violence: Measuring Success in Ways that Matter to Children, Parents and Professionals. *Child Abus Rev* 2015;**24**:297–310. doi:10.1002/car.2408

15 Williamson PR, Altman DG, Bagley H, *et al.* The COMET Handbook: version 1.0. *Trials* 2017;**18**:280. doi:10.1186/s13063-017-1978-4

16 Davis K, Gorst SL, Harman N, *et al.* Choosing important health outcomes for comparative effectiveness research: An updated systematic review and involvement of low and middle income countries. *PLoS One* 2018;**13**. doi:10.1371/journal.pone.0190695

17 Gorst SL, Gargon E, Clarke M, *et al.* Choosing important health outcomes for comparative effectiveness research: An updated review and user survey. *PLoS One* 2016;**11**. doi:10.1371/journal.pone.0146444

18 Hamby S, Finkelhor D, Turner H, *et al.* The overlap of witnessing partner violence with child maltreatment and other victimizations in a nationally representative survey of youth. *Child Abuse Negl* 2010;**34**:734–41. doi:http://dx.doi.org/10.1016/j.chiabu.2010.03.001

19 Henry C. Exposure to domestic violence as abuse and neglect: Constructions of child maltreatment in daily practice. *Child Abus Negl* 2018;**86**:79–88. doi:10.1016/j.chiabu.2018.08.018

20 Lawson J. Domestic violence as child maltreatment: Differential risks and outcomes among cases referred to child welfare agencies for domestic violence exposure. *Child Youth Serv Rev* 2019;**98**:32–41. doi:10.1016/j.childyouth.2018.12.017

21 Kirkham JJ, Gorst S, Altman DG, *et al.* Core Outcome Set-STAndardised Protocol Items: the COS-STAP Statement. *Trials* 2019;**20**:116. doi:10.1186/s13063-019-3230-x

22 Woodman J, Simon A, Hauari H, *et al.* A scoping review of 'think-family' approaches in healthcare settings. *J Public Health (Bangkok)* Published Online First: 22 January 2019. doi:10.1093/pubmed/fdy210

23 England MJ, Butler AS, Gonzalez ML, *et al.* *Psychosocial interventions for mental and*

For peer review only

For peer review only - http://bmjopen.bmj.com/site/about/guidelines.xhtml

substance use disorders : a framework for establishing evidence-based standards.

24 Leamy M, Bird V, Le Boutillier C, *et al.* Conceptual framework for personal recovery in mental health: Systematic review and narrative synthesis. Br. J. Psychiatry. 2011;**199**:445–52. doi:10.1192/bjp.bp.110.083733

25 Gargon E, Gurung B, Medley N, *et al.* Choosing important health outcomes for comparative effectiveness research: A systematic review. *PLoS One* 2014;**9**. doi:10.1371/journal.pone.0099111

26 Sinha I, Jones L, Smyth RL, *et al.* A systematic review of studies that aim to determine which outcomes to measure in clinical trials in children. *PLoS Med* 2008;**5**:0569–78. doi:10.1371/journal.pmed.0050096

27 Sinha IP, Smyth RL, Williamson PR. Using the Delphi technique to determine which outcomes to measure in clinical trials: recommendations for the future based on a systematic review of existing studies. *PLoS Med* 2011;**8**:e1000393. doi:10.1371/journal.pmed.1000393

28 Macefield R, Blencowe N, Brookes S, *et al.* Core outcome set development: the effect of Delphi panel composition and feedback on prioritisation of outcomes. *Trials* 2013;**14**:P77. doi:10.1186/1745-6215-14-S1-P77

29 Thiara RK, Hague G, Mullender A. Losing out on both counts: Disabled women and domestic violence. *Disabil Soc* 2011;**26**:757–71. doi:10.1080/09687599.2011.602867

30 Anitha S. No Recourse, No Support: State Policy and Practice Towards South Asian Women Facing Domestic Violence in the UK. 2010. doi:bcn160

31 Douglas H, Walsh T. Mandatory Reporting of Child Abuse and Marginalised Families. Springer, Dordrecht 2015. 491–509. doi:10.1007/978-94-017-9685-9_23

32 Brophy J. Child maltreatment in diverse households: Challenges to law, theory, and practice. *J Law Soc* 2008;**35**:75–94. doi:10.1111/j.1467-6478.2008.00415.x

33 Ganann R, Ciliska D, Thomas H. Expediting systematic reviews: Methods and implications of rapid reviews. *Implement Sci* 2010;**5**:56. doi:10.1186/1748-5908-5-56

34 Schünemann HJ, Moja L. Reviews: Rapid! Rapid! Rapid!.and systematic. Syst. Rev. 2015;**4**:4. doi:10.1186/2046-4053-4-4

35 Howarth E, Moore THM, Welton NJ, *et al.* Improving outcomes for children exposed to domestic violence (IMPROVE): An evidence synthesis. *Public Heal Res* 2016;**4**.

For peer review only

doi:10.3310/phr04100

36 Arai L, Heawood A, Feder G, *et al.* Hope, Agency, and the Lived Experience of Violence: A Qualitative Systematic Review of Children's Perspectives on Domestic Violence and Abuse. *Trauma, Violence, Abus* 2019;:152483801984958. doi:10.1177/1524838019849582

37 Braun V, Clarke V. Using thematic analysis in psychology. *Qual Res Psychol* 2006;**3**:77–101.

38 Braun V, Clarke V, Boulton E, *et al.* The online survey as a *qualitative* research tool. *Int J Soc Res Methodol* 2020;:1–14. doi:10.1080/13645579.2020.1805550

39 Dodd S, Clarke M, Becker L, *et al.* A taxonomy has been developed for outcomes in medical research to help improve knowledge discovery. *J Clin Epidemiol* 2018;**96**:84–92. doi:10.1016/J.JCLINEPI.2017.12.020

40 Heise LL. What works to prevent partner violence. *Dfid* 2011;:1–110. doi:978 0 902657 85 2

41 Heise LL. Violence against women: An integrated, ecological framework. *Violence Against Women* 1998;**4**:262–90. doi:10.1177/1077801298004003002

42 Belsky J. Etiology of child maltreatment: A developmental-ecological analysis. *Psychol Bull* 1993;**114**:413–34. doi:10.1037/0033-2909.114.3.413

43 van IJzendoorn MH, Bakermans-Kranenburg MJ, Coughlan B, *et al.* Annual Research Review: Umbrella synthesis of meta-analyses on child maltreatment antecedents and interventions: differential susceptibility perspective on risk and resilience. J. Child Psychol. Psychiatry Allied Discip. 2020;**61**:272–90. doi:10.1111/jcpp.13147

44 Blazeby JM, Macefield R, Blencowe NS, *et al.* Core information set for oesophageal cancer surgery. *Br J Surg* 2015;**102**:936–43. doi:10.1002/bjs.9840

45 Gargon E, Crew R, Burnside G, *et al.* Higher number of items associated with significantly lower response rates in COS Delphi surveys. *J Clin Epidemiol* 2019;**108**:110–20. doi:10.1016/j.jclinepi.2018.12.010

46 Sherratt FC, Eaton S, Walker E, *et al.* Development of a core outcome set to determine the overall treatment success of acute uncomplicated appendicitis in children: A study protocol. *BMJ Paediatr Open* 2017;**1**:e000151. doi:10.1136/bmjpo-2017-000151

47 Knaapen M, Hall NJ, Van Der Lee JH, *et al.* Establishing a core outcome set for treatment of uncomplicated appendicitis in children: Study protocol for an international Delphi survey. *BMJ Open* 2019;**9**:e028861. doi:10.1136/bmjopen-2018-028861

For peer review only

LEGEND

Figure 1: Study design

ACKNOWLEDGEMENTS

First and foremost we thank the survivors and other members of the public who contributed to this study. Survivor involvement is facilitated by VOICES, a Survivor led charity for women who have experienced domestic abuse. We also extend our thanks to members of our advisory groups which have informed the development of the study design and have commented on drafts of this manuscript. Members of the professional advisory group are as follows: Elaine Fulton, Dr Deborah Hodes, Dr Carol Rivas, Professor Sally Kendall, David and Elisabeth Carney Haworth and Hannah Edwards.

AUTHOR CONTRIBUTIONS

EH conceived of the original study design, which was refined and developed by all authors. CP developed protocols for rapid reviews, which were reviewed and refined by all authors. All study authors contributed to the writing and review of the protocol paper.

FUNDING STATEMENT

This study is funded by the National Institute for Health Research (NIHR) Policy Research Programme. Grant title: NIHR Children and Families Policy Research Unit. NIHR funder reference: PR-PRU-1217-21301. The views expressed are those of the author(s) and not necessarily those of the NIHR or the Department of Health and Social Care.

COMPETING INTERESTS

We have read and understood BMJ policy on declaration of interests and declare that we have no competing interests.

DATA SHARING STATEMENT

Please contact the corresponding author or unit manager cpru.data@ucl.ac.uk with enquiries about the data used in this study.

SUPPLEMENTARY FILE

Rapid review protocols

For peer review only

Figure 1: Study design

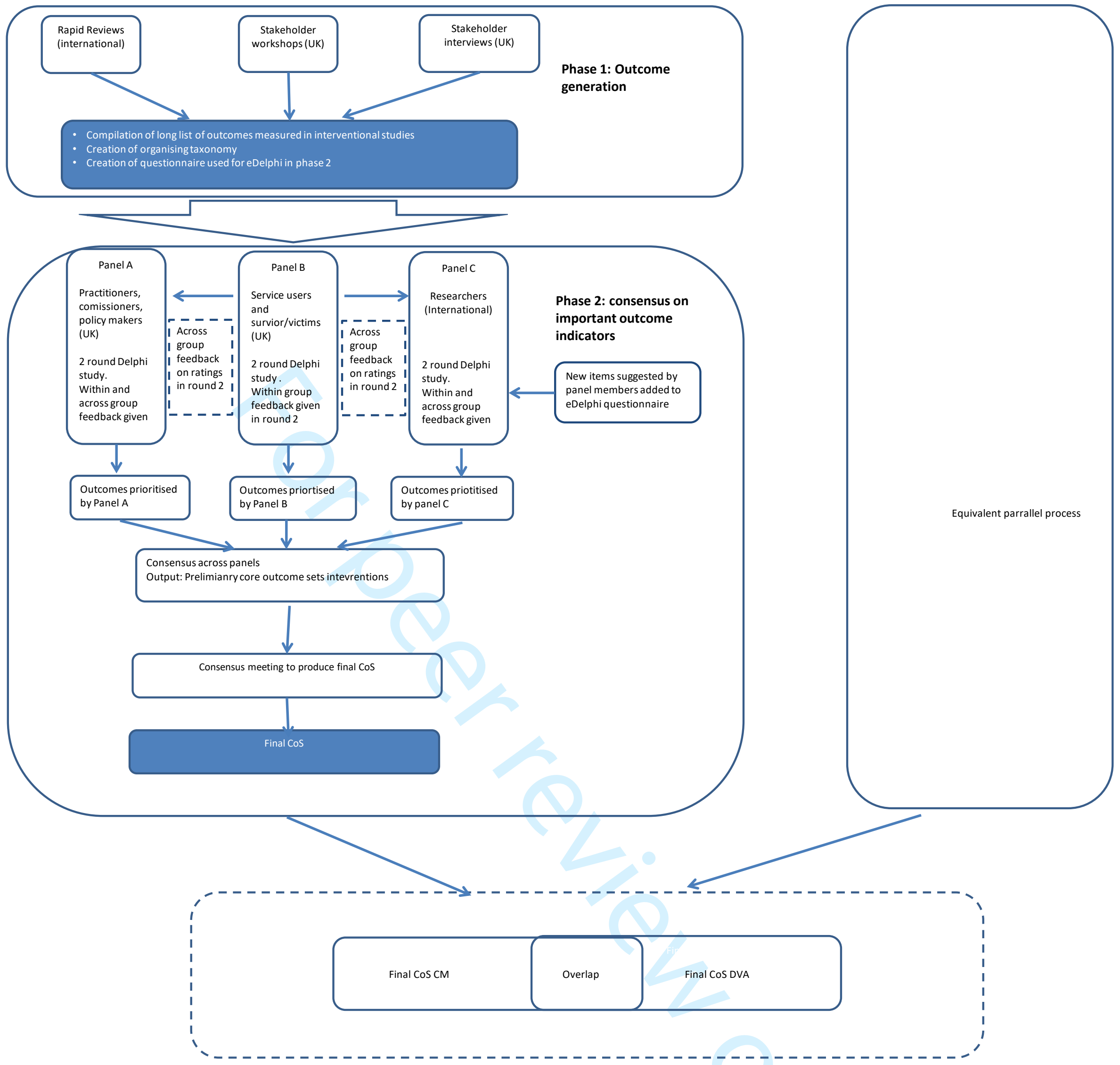

Supplementary Appendices – Rapid Review Protocols

A - Rapid review of systematic reviews of intervention studies

Review question: How are outcomes defined and measured in controlled trials of interventions aiming to improve outcomes of children and families with children exposed to DVA/CM and those aiming to reduce subsequent abusive behaviour by perpetrators of DVA/CM?

a. This includes the definition and measurement of DVA/CM.

This rapid review will be carried out in two steps: firstly searches for systematic reviews (SR) will be carried out, then these reviews will be used to extract individual studies which will be screened for relevance. This process will be carried out in parallel for the DVA and CM literature.

Study inclusion: Peer-reviewed systematic reviews of controlled or quasi experimental comparator intervention studies: with or without randomisation.

The DARE criteria for SRs are at least 4 of the following: reporting of inclusion/exclusion criteria; adequate search; synthesis of included studies; quality assessment of studies; sufficient detail presented (CRD, 1995). For the purposes of this review, SRs will be included if they use an electronic database and have a structured search strategy.

- Published since 2014.
- No restrictions by country. English language only.

- Individual studies must include DVA/CM in one of the following ways:
    - Entry to the intervention is determined by experience, perpetration or identified as at risk of DVA/CM. (Identification of risk is by researchers, practitioners or participants thus we do not have a definition)
    - Subgroup analysis is carried out of participants who have experienced (or are considered to be at risk of) DVA/CM
    - DVA/CM is measured as an exposure (this could be retro or prospectively reported)

Exclusion: Non peer-reviewed studies, qualitative studies, general literature reviews, protocols, case reports, cross-sectional studies, general discussion papers, letters, commentaries, book chapters, conference papers, theses and dissertations.

Population inclusion: children or families with children at risk of experiencing, or experiencing DVA/CM'. This includes unborn children, children (aged 0 to 18 years), designated as victim or witness. For DVA any adult family members who have a parenting role (Early Intervention Foundation, 2014), whether designated as perpetrator, victim, witness or household member. For CM any adult family members who have a caring role, whether designated as maltreating parent, witness or household member. These adults and children could either be the primary study population of interest or form a subgroup in a wider study population.

Intervention inclusion: Any interventions or services where:

For peer review only - http://bmjopen.bmj.com/site/about/guidelines.xhtml

- Experience of or increased risk of experiencing DVA/CM is a criterion for being offered the service
  OR
- DVA/CM is measured as an exposure or outcome of interest
  AND
- At least one child or family-level outcome is measured. Family-level outcomes do not need to be explicitly labelled as 'family' level, we will make a judgement. However, they include any outcome that affects the family/household unit. For example, worklessness in study where at least some participants are reported to be parents would be included.

- Studies must include evaluation of a defined activity/programme and evaluation of a hypothesised effect
- Interventions may be delivered to any family member(s) as an individual or in a group. Any duration of intervention will be included. Any setting will be considered.

Exclusion: universal interventions that do not specifically target children and families at risk of DVA/CM; targeted interventions that do not measure any child or family level outcomes e.g. perpetrator programmes that focus solely on attitudinal change; DVA (only) interventions focused solely on elder abuse, sibling abuse or child perpetration of domestic violence where participants have not been identified as exposed to DVA.

Comparator inclusion: Any control or comparison group/period with participants receiving no care, treatment as usual or any other treatment.

Outcome inclusion:
- Any child outcome related to i) the child's experience of adversity ii) child functioning, including risky behaviours (see (Maclean et al., 2016) for full list of health and wellbeing outcomes).
- Any outcomes related to the quality of the caregiving environment (e.g. parenting, maternal depression, stressful life events, maternal psychological distress, parental substance misuse).
- Any outcomes related to material deprivation e.g. low income, economic hardship or stress (including perceived), social capital, hunger, food poverty, housing instability.
- Any other outcome judged to relate to children or families by the research team.
- Outcomes can be reported by professionals, child, parent or other family member and they can be retrospective or prospective.
- Outcomes can be end points, surrogate markers for end points or intermediate outcomes.
- No minimum or maximum follow-up is required.

Context inclusion: Studies from any country in any setting.

Searches
The following electronic databases will be searched from 2014: Medline, Embase, PsycInfo, Cochrane and Web of Science. Searching will include expert recommendations of relevant broader studies, including relevant parenting programmes.

The search strategy will include MeSH terms relating to DVA/CM and the BMJ systematic review strategy ((*Study Design Search Filters | BMJ Best Practice*, n.d.)). Key word terms for DVA/CM, abuse,

For peer review only - http://bmjopen.bmj.com/site/about/guidelines.xhtml

violence, family members and systematic reviews will be used. These have been developed from the two main NIHR-funded studies in the area ((Howarth et al., 2016) and (Macdonald et al., 2016)) and adapted as required for the different databases with guidance from an expert librarian.

These reviews will be carried out separately for DVA and CM. The DVA search will be run first and any CM studies that do not mention DVA will be excluded (and vice versa). As part of the review involves collecting definitions of DVA/CM, any study deemed to fit within the umbrella by the research team will be included.

Data extraction (selection and coding)
All systematic reviews identified by database searches will be downloaded to CADIMA (Kohl et al., 2018) and de-duplicated. Screening criteria will be tested by two reviewers on 200 titles/abstracts and interrater reliability assessed. Titles/abstracts will be screened by one reviewer for inclusion in full-text review. A second reviewer will independently review 10% of title/abstracts. If there is a high level of disagreement, the second reviewer will continue reviewing titles/abstracts until agreement is reached. Full-text systematic reviews will be screened for inclusion and a second reviewer will independently review 10% of these as above. Key data from the systematic reviews (e.g. definition of DVA/CM) will be extracted into CADIMA by one reviewer.

Individual studies will be extracted from the included full-text systematic reviews. These studies will be downloaded to Zotero and de-duplicated. The remaining studies will then be screened for inclusion in full-text review and data extraction. Data will be extracted into Access using a standardised form and a second researcher will review extraction from the first 5 papers. The following data will be extracted: bibliographic information, study design, setting, sample characteristics, definitions of DVA/CM, intervention details, primary and secondary outcomes (applicable for children and families) and their measures, descriptions of mechanisms. (Where DVA/CM is not measured as an outcome, nor is there a subgroup analysis, only exposure definition will be extracted.) Quality control/risk of bias will not be assessed because the aim of the review is solely to collect outcomes.

Strategy for data synthesis
Narrative synthesis and tabulation of outcomes extracted.

B - Rapid review of qualitative studies

Review questions:

1) What outcomes (benefits or harms) are sought or experienced by actual or potential recipients of interventions/services aiming to prevent or reduce the risk of harm associated with DVA/CM?

2) What outcomes (benefits or harms) are sought by stakeholders* involved in developing and/or delivering interventions to children/families experiencing DVA/CM?

**'stakeholder' is defined as in the IMPROVE study i.e. young people with experience of DVA/CM services, parents/caregivers with experience of using DVA/CM services or professionals involved in commissioning and delivering services to families affected by DVA/CM.*

For peer review only - http://bmjopen.bmj.com/site/about/guidelines.xhtml

This review will be carried out in parallel for DVA and CM.

Study inclusion:

- Primary qualitative (i.e. analysis of interviews, focus groups or other verbal analysis which is not quantified) intervention studies either as a standalone study or a discrete component of mixed-method studies.
- Direct and sufficient verbatim text from participants for analysis (i.e. more than two lines) c.f. Arai et al. (2019).
- Published since October 2015 (DVA) and July 2014 (CM) to build on Howarth et al. (2016) and Macdonald et al. (2016).
- No restrictions by country. English language only.

- Individual studies must include DVA/CM in one of the following ways:
    - Participation in the study is determined by experience, perpetration or specifically identified as at risk of DVA/CM. Participants may have received an intervention or may be discussing the impact of DVA/CM and their desired outcomes for the future. (To ensure we are not limited by outcomes defined by current interventions).

    OR
    - Stakeholders involved in developing and/or delivering interventions to children/families experiencing DVA/CM (c.f. Howarth et al, 2016, p.52), or stakeholder discussion of outcomes that are sought either in relation to an intervention or the future in general.

Exclusion: Non peer-reviewed studies, surveys or quantitative studies with descriptive free-text only, general literature reviews, case reports, general discussion papers, letters, commentaries, editorials, book chapters, conference papers, theses and dissertations.

Population inclusion: Any adult or child stakeholders relevant to DVA/CM. This could be as a result of experience, perpetration, identified as at risk, delivering, commissioning or intending to deliver services.

Phenomenon of interest: DVA/CM

Design: Any qualitative approach to data collection and analysis (e.g. interviews, focus groups)

Evaluation: Perspectives of experienced or anticipated benefits or harms of interventions, and/or desired outcomes in general related to DVA/CM.

Searches

The following electronic databases as advised for qualitative research (Evans, 2002; McFadden et al, 2012; Booth, 2016) will be searched from October 2015 (DVA) and July 2014 (CM): ASSIA, CINAHL, GoogleScholar (first 100 hits), PsycInfo and SSCI.

This review is building on Howarth et al. (2016) and Macdonald et al. (2016) so relevant studies from these reviews (and related work such as Arai et al. (2019)) will be included. In addition, expert recommendations of relevant qualitative studies or reviews and any qualitative studies identified from the reviews of systematic reviews will be included.

For peer review only - http://bmjopen.bmj.com/site/about/guidelines.xhtml

The search strategy will use the same terms for DVA/CM as the review of systematic reviews, plus additional search terms to identify qualitative research. These will be adapted as required for the different databases with guidance from an expert librarian.
These reviews will be carried out separately for DVA and CM. The DVA search will be run first and any CM studies that do not mention DVA will be excluded (and vice versa) but put aside for inclusion in the relevant review. This review will not adhere to set definitions of DVA/CM, thus any study deemed by the research team to address the phenomena of interest will be included and justified in the discussion of findings.

Screening
Screening of abstracts from the searches and articles included in the full text stage will be guided by questions asked in the IMPROVE study (Howarth et al., 2016):

1) Is this qualitative research?
2) Is there sufficient verbatim text? (i.e. more than 2 lines)
3) Does the paper discuss perspectives of experienced or anticipated benefits or harms of interventions, and/or desired outcomes in general related to DVA/CM.

All articles identified by searches will be downloaded to CADIMA (Kohl et al., 2018) and de-duplicated. Screening criteria will be tested by two reviewers on 10% titles/abstracts and interrater reliability assessed. Titles/abstracts will be screened by one reviewer for inclusion in full-text review. A second reviewer will independently review 10% of title/abstracts. If there is a high level of disagreement, the second reviewer will continue reviewing titles/abstracts until agreement is reached. Full-text systematic reviews will be screened for inclusion and a second reviewer will independently review 10% of these as above. Key details (e.g. bibliographic information, study design, setting, participants etc.) about each full-text inclusion will be recorded in Access.

Strategy for data synthesis
Thematic frameworks will be developed from the IMPROVE study (Howarth et al., 2016) for DVA and the parallel CM study (MacDonald et al., 2016), and input into NVivo 11 (QSR International). The frameworks will focus on barriers and harms of interventions according to parents, children and stakeholders, based on the research questions. These will be used as the basis for a framework analysis (Ritchie & Lewis, 2003) of the studies from the review (Howarth et al., 2016; Arai et al., 2019; Macdonald et al., 2016). As per Howarth et al. (2016), participant quotations and author-identified themes will be extracted rather than line by line coding. Findings will be grouped by whose view was reported and extracts from the texts will be categorised according to this framework with the aim will be to meta-aggregate the studies' findings. Further categories will be developed where there are discrepancies or gaps in the initial framework.

The analysis and interpretation of the findings will occur at the synthesis stage in order to provide an overview of the findings, informed by the principles of meta-synthesis (c.f. Noblit & Hare, 1988), although using a lighter touch given time constraints. Two researchers will work together throughout this process to ensure consistency of categorisation and analysis. Quality will not be assessed because the aim of the review is solely to identify candidate outcomes. The ENTREQ statement (Tong et al., 2012) will be followed for the write-up.

For peer review only - http://bmjopen.bmj.com/site/about/guidelines.xhtml

C – Rapid review of grey literature

Review questions:

1) How are DVA and CM defined in relevant UK service policy contexts?
2) How are outcomes defined: (i) in UK service-based evaluations of interventions? (ii) in relevant policy or commissioning frameworks?

This review will be carried out as a single process given the likelihood of crossover literature. Findings will be recorded as DVA or CM or both.

Literature inclusion:

- Any national or regional policy or practice document that reports on DVA/CM-relevant services or outcomes (e.g. measurement/theory).
- Participation in the service is determined by experience, perpetration or identified as at risk of DVA/CM. (Identification of risk is by practitioners or participants thus we do not have a definition).
- Published since 2016 to build on Howarth et al. (2016) and Macdonald et al. (2016).
- England-based only. English language only.

Exclusion: Publication in academic journals, book chapters, conference papers, theses and dissertations.

Population inclusion: children or families with children at risk of experiencing, or experiencing DVA/CM. This includes unborn children, children (aged 0 to 18 years), designated as victim or witness. For DVA any adult family members who have a caring or parenting role (Early Intervention Foundation, 2014), whether designated as perpetrator, victim, witness or household member. For CM any adult family members who have a caring role, whether designated as perpetrator, witness or household member.

Service inclusion: Any services where:

- Experience of or increased risk of experiencing DVA/CM is a criterion for being offered the service/intervention.
- Services/interventions may be delivered to any family member(s) as an individual or in a group. Any duration of service/intervention will be included. Any setting will be considered.

OR

- Any evaluative work or outcomes framework where at least one child or family-level outcome is evaluated/discussed. Family-level outcomes do not need to be explicitly labelled as 'family' level, we will make a judgement. However, they include any outcome that affects the family/household unit. For example, worklessness in study where at least some participants are reported to be parents would be included.

Exclusion: universal services/interventions that do not specifically target children and families at risk of DVA/CM; targeted services/interventions that do not measure any child or family level outcomes e.g. perpetrator programmes that focus solely on attitudinal change; DVA (only) services/interventions focused solely on elder abuse, sibling abuse or child perpetration of domestic violence, where participants have not been identified as exposed to DVA (i.e. perpetration of abuse by a child could feasibly be an outcome associated with exposure).

For peer review only - http://bmjopen.bmj.com/site/about/guidelines.xhtml

Outcome inclusion: Any family or child-level outcome measured or evaluated or discussed in any way. Intermediate outcomes that could feasibly represent preconditions needed to reach distal/final outcomes (including those relating to the process of service delivery) will be included, along with final/distal outcomes.

Searches

The following databases and websites will be searched:

Grey databases: NICE Evidence Search and Open Grey

Organisation websites including but not limited to:

DVA: Women's Aid, Refuge, Respect, Safe Lives, Voices, AVA, Standing Together, Imkaan, The Stefanou Foundation, Women's Trust, Hestia, DVIP, Nia, The Haven, ManKind Initiative, Everyman Project, NCDV, Galop, LAWA, IDAS, Advance, Your Sanctuary, Advocacy After Fatal Domestic Abuse (AAFDA); Aurora New Dawn; My Sister's Place

CM: Centre of expertise on child sexual abuse, FDAC, SCIE, The Survivors' Trust

General websites: Victim Support, Barnardos, NSPCC, Early Intervention Foundation, NatCen, RCGP, RCN, RCM, NICE, BPS, IHV, WHO, UNICEF, Working together, gov.uk (incls e.g. DA bill, 'Working together'), Public Health for any UK nation, Office of the children's commissioner for any UK nation, Big Lottery, Comic Relief, The Childhood Trust, UK College of Policing, Research in Practice, 'What Works', Joseph Rowntree Foundation, What Works for Children's Social Care.

Websites will be searched manually for relevant documents. It is anticipated there will be an element of snowball searching as relevant organisations will have links to further organisations. Searches will be run simultaneously and then relevant reports assigned to DVA/CM or both. All websites searched will be recorded in Excel/Access along with relevant details about any reports captured. The expert reference group will be consulted about relevant websites to search or reports to include at multiple timepoints.

Data extraction and synthesis

As a range of types of data are anticipated, both the systematic review and the qualitative review protocols will be adapted as necessary to capture and record relevant information. It is likely that there will be non-standardised evaluation measures and interview quotations. Report identification from websites/databases will be carried out by a single researcher and the process transparently recorded. All details regarding evaluation studies and relevant outcomes will be recorded, and where necessary synthesised when the data is qualitative. Access/Excel/NVivo will be used as required to record all steps and ensure a transparent process. A second researcher will cross-check a subset of the reports and the data extracted to ensure consistency and focus on the review questions.

For peer review only - http://bmjopen.bmj.com/site/about/guidelines.xhtml

Bibliography

Arai, L., Heawood, A., Feder, G., Howarth, E., MacMillan, H., Moore, T. H. M., Stanley, N., & Gregory, A. (2019). Hope, Agency, and the Lived Experience of Violence: A Qualitative Systematic Review of Children's Perspectives on Domestic Violence and Abuse. *Trauma, Violence, & Abuse*, 152483801984958. https://doi.org/10.1177/1524838019849582

CRD. (1995). *Database of Abstracts of Reviews of Effects (DARE): Quality-assessed Reviews*. Centre for Reviews and Dissemination (UK).

Early Intervention Foundation. (2014). *Early Intervention in Domestic Violence and Abuse*.

Howarth, E., Moore, T. H., Welton, N. J., Lewis, N., Stanley, N., MacMillan, H., Shaw, A., Hester, M., Bryden, P., & Feder, G. (2016). IMPRoving Outcomes for children exposed to domestic ViolencE (IMPROVE): An evidence synthesis. *Public Health Research*, *4*(10), 1–342. https://doi.org/10.3310/phr04100

Kohl, C., McIntosh, E. J., Unger, S., Haddaway, N. R., Kecke, S., Schiemann, J., & Wilhelm, R. (2018). Online tools supporting the conduct and reporting of systematic reviews and systematic maps: A case study on CADIMA and review of existing tools. *Environmental Evidence*, *7*(1), 8. https://doi.org/10.1186/s13750-018-0115-5

Macdonald, G., Livingstone, N., Hanratty, J., McCartan, C., Cotmore, R., Cary, M., Glaser, D., Byford, S., Welton, N. J., Bosqui, T., Bowes, L., Audrey, S., Mezey, G., Fisher, H. L., Riches, W., & Churchill, R. (2016). The effectiveness, acceptability and cost-effectiveness of psychosocial interventions for maltreated children and adolescents: An evidence synthesis. *Health Technology Assessment*, *20*(69), 1–508. https://doi.org/10.3310/hta20690

Maclean, M. J., Sims, S., O'Donnell, M., & Gilbert, R. (2016). Out-of-Home Care versus In-home Care for Children Who Have Been Maltreated: A Systematic Review of Health and Wellbeing Outcomes: Systematic Review of Outcomes of Out-of-Home Care. *Child Abuse Review*, *25*(4), 251–272. https://doi.org/10.1002/car.2437

Study design search filters | BMJ Best Practice. (n.d.). Retrieved 16 April 2019, from https://bestpractice.bmj.com/info/us/toolkit/learn-ebm/study-design-search-filters/

Tong, A., Sainsbury, P., & Craig, J. (2007). Consolidated criteria for reporting qualitative research (COREQ): A 32-item checklist for interviews and focus groups. *International Journal for Quality in Health Care*, *19*(6), 349–357. https://doi.org/10.1093/intqhc/mzm042

For peer review only

Protocol for developing Core Outcome Sets for evaluation of psychosocial interventions for children and families with experience or at risk of child maltreatment or domestic abuse

Journal:	*BMJ Open*
Manuscript ID	bmjopen-2020-044431.R1
Article Type:	Protocol
Date Submitted by the Author:	28-Feb-2021
Complete List of Authors:	Howarth, Emma; University of East London; University College London, Institute of Child Health<br>Powell, Claire; University College London, Institute of Child Health<br>Woodman, Jenny; University College London, Institute of Education<br>Walker, Erin; University College London, UCL Partners<br>Chesters, Heather; University College London, Institute of Child Health<br>Gilbert, Ruth; UCL Institute of Child Health, Centre for Paediatric Epidemiology and Biostatistics<br>Feder, Gene; University of Bristol, Community based medicine
**Primary Subject Heading**:	Public health
Secondary Subject Heading:	Research methods
Keywords:	Child protection < PAEDIATRICS, Community child health < PAEDIATRICS, PUBLIC HEALTH, SOCIAL MEDICINE

I, the Submitting Author has the right to grant and does grant on behalf of all authors of the Work (as defined in the below author licence), an exclusive licence and/or a non-exclusive licence for contributions from authors who are: i) UK Crown employees; ii) where BMJ has agreed a CC-BY licence shall apply, and/or iii) in accordance with the terms applicable for US Federal Government officers or employees acting as part of their official duties; on a worldwide, perpetual, irrevocable, royalty-free basis to BMJ Publishing Group Ltd ("BMJ") its licensees and where the relevant Journal is co-owned by BMJ to the co-owners of the Journal, to publish the Work in this journal and any other BMJ products and to exploit all rights, as set out in our licence.

The Submitting Author accepts and understands that any supply made under these terms is made by BMJ to the Submitting Author unless you are acting as an employee on behalf of your employer or a postgraduate student of an affiliated institution which is paying any applicable article publishing charge ("APC") for Open Access articles. Where the Submitting Author wishes to make the Work available on an Open Access basis (and intends to pay the relevant APC), the terms of reuse of such Open Access shall be governed by a Creative Commons licence – details of these licences and which Creative Commons licence will apply to this Work are set out in our licence referred to above.

Other than as permitted in any relevant BMJ Author's Self Archiving Policies, I confirm this Work has not been accepted for publication elsewhere, is not being considered for publication elsewhere and does not duplicate material already published. I confirm all authors consent to publication of this Work and authorise the granting of this licence.

For peer review only

TITLE **Protocol for developing Core Outcome Sets for evaluation of psycho-social interventions for children and families with experience or at risk of child maltreatment or domestic abuse**

Emma Howarth (Corresponding author)
School of Psychology, UEL
University Way, London, UK E16 2RD
Email: e.howarth@uel.ac.uk

Claire Powell
Institute of Child Health, UCL
30 Guilford St, London, UK WC1N 1EH

Jenny Woodman
Institute of Education, UCL
27/8 Woburn Square, London, UK WC1H 0AA

Erin Walker
UCL Partners
170 Tottenham Court Road, London, UK W1T 7HA

Heather Chesters
Institute of Child Health, UCL
30 Guilford St, London, UK WC1N 1EH
United Kingdom

Ruth Gilbert
Institute of Child Health, UCL
30 Guilford St, London, UK WC1N 1EH
United Kingdom

Gene Feder
School of Social and Community Medicine, University of Bristol
39 Whatley Road, Bristol BS8 2PS
United Kingdom

KEY WORDS Core outcome set; intervention; child maltreatment; domestic abuse; evaluation

WORD COUNT 5119

For peer review only

ABSTRACT

Introduction

Recognition that child maltreatment (CM) and domestic violence and abuse (DVA) are common and have serious and long-term adverse health consequences, has resulted in policies and programmes to ensure that services respond to and safeguard children and their families. However, high quality evidence about how services can effectively intervene is scant. The value of the current evidence base is limited partly because of the variety of outcomes and measures used in evaluative studies. One way of addressing this limitation is to develop a Core Outcome Set (COS) which is measured and reported as a minimum standard in the context of trials and other types of evaluative research. The study described in this protocol aims to develop two discrete core outcome sets for use in future evaluation of psycho-social interventions aimed at improving outcomes for children and families at risk or with experience of i) CM or ii) DVA.

Methods and analysis

A two-phase mixed methods design: 1) rapid reviews of evidence, stakeholder workshops and semi-structured interviews with adult survivors of CM/DVA and parents of children who have experienced CM/DVA; 2) a three panel adapted E-Delphi study and consensus meeting. This study protocol adheres to reporting guidance for COS protocols and has been registered on the COMET database.

Ethics and dissemination

We will disseminate our findings through peer reviewed and open access publications, the COMET website, and presentations at international conferences. We will engage with research networks, journal editors and funding agencies to promote awareness of the CM- and DVA-COS. We will work with advisory and survivor and public involvement groups to co-produce a range of survivor, policy and practice facing outputs.

Approval for this study has been granted by the Research Ethics Committee at University College London.

STRENGTHS AND LIMITATIONS OF THIS STUDY

- To our knowledge this is the first attempt to develop core outcome sets to address family violence and abuse.

For peer review only - http://bmjopen.bmj.com/site/about/guidelines.xhtml

For peer review only

- The study draws on diverse evidence sources and includes people with lived experience, practitioners and policy makers, as well as researchers.
- This study provides the opportunity to consider the overlap in outcomes sought across two different but related exposures.
- This study is limited by the lack of direct involvement of children and young people.
- It is beyond the means of the study to involve survivors and service providers from low- and middle-income countries (LMICs), although we will include research from LMICs in the evidence reviews and actively recruit researchers from or researching LMIC settings.

For peer review only

For peer review only - http://bmjopen.bmj.com/site/about/guidelines.xhtml

INTRODUCTION

Widespread recognition that child maltreatment (CM) and domestic violence and abuse (DVA) are common and have serious and long-term adverse health consequences, [1,2] has resulted in policies and programmes to ensure that services respond to and safeguard children (and their families) at high risk of, or with experience of CM and or/DVA.[3–6] However, high quality evidence about how services can *effectively* intervene is scant. [7–9]

The value of the current evidence base is limited partly because of the variety of outcomes and measures used in evaluative studies. [7,8]. This hampers the ability to aggregate evidence pertaining to one particular type of intervention, so as to build a comprehensive picture of its effectiveness when delivered to different populations or in different contexts. Similarly it is challenging to make comparisons between different types of interventions, which purport to address the same problem within the same group of individuals. [10,11]

More fundamentally, outcomes measured in CM and DVA intervention studies are often a poor or partial reflection of the concepts of success held by those that use, deliver and pay for interventions. [7,8,12] The ultimate goal of intervention studies is to identify interventions that can benefit individuals, families and communities in the future. Therefore, it is crucial that they measure outcomes reflecting the priorities and expectations of these groups so the evidence they generate is relevant to consumers. Outcomes also need to resonate with the priorities of policymakers and service providers, else effective interventions may be overlooked by those responsible for funding and/or delivery decisions, and never commissioned or implemented.[13,14]

Together, these issues mean it is difficult to extract the information needed to inform real world decisions about which CM/DVA interventions to commission and scale, and which to stop funding.

One way of addressing the limitations set out above is to develop a Core Outcome Set (COS), a standardised set of outcomes that researchers, providers, service users, and commissioners consider critical or important outcomes in the management of a condition or in this case, a complex public health challenge. [11,15] The COS is then measured and reported, as a minimum standard in the context of trials or other types of research and evaluation, [15] and sometimes practice-based monitoring. [16] The aim is to enhance the methodological standard and utility of research in the field, by increasing consistency and reducing reporting bias (where many outcomes are measured and only favourable effects reported), and ensuring the views of important constituencies influence the selection of outcomes to be included in the COS. [10]

For peer review only - http://bmjopen.bmj.com/site/about/guidelines.xhtml

The idea of the COS as a mechanism for improving evidence quality has gathered momentum over the past decade since the establishment of the COMET (Core Outcome Measures for Effectiveness Trials) initiative in 2010 (www.comet-initiative.org). [15] Whilst the number of core outcome sets being developed has increased steadily since, [16,17] it is clear that in the main, studies have focused on COS development for specific health conditions, pharmacological or surgical interventions and/or discrete interventions delivered in health care settings. [16,17] In contrast there has been relatively less focus on the development of COSs in relation to public health problems that require complex multi-sectoral responses, often delivered to whole families or multiple members of the same family.

Current study

The study sets out to develop two discrete core outcome sets for use in future evaluation of psycho-social interventions, which aim to improve outcomes for children and families at risk of or with experience of CM or DVA. We use the term 'at risk' so as not to limit the scope of this work to those interventions delivered to families following substantiated experience of CM or DVA or where children and families define their experiences as such; but to include interventions offered to families where it is suspected that an exposure may have taken place, or where children's experiences are thought to be on a trajectory towards this.

Children's experiences of CM and DVA frequently overlap [18] and experience of DVA is often conceived of as a type of maltreatment in its own right, or a feature of emotional maltreatment. [19,20] Nevertheless, the conceptualisation and response to these two types of trauma can be different, despite similar consequences. For example, there is variation as to whether exposure to DVA is considered as a form of child maltreatment. Where it is, evidence suggests there may be different levels of state intervention where the primary concern is exposure to DVA versus experience of CM. [19,20]. This provides the rationale for developing separate outcome sets, however we will explore where the derived outcome sets overlap with a view to identifying outcomes that can be measured in family contexts where *both* CM and DVA occur. This is a move away from a focus on single problem areas towards recognition of the constellation of risks often experienced by children and their families.

METHODS AND ANALYSIS

This study protocol adheres to reporting guidance for core outcome set protocols [21] and has been registered on the COMET database.

Scope of outcome sets

For peer review only - http://bmjopen.bmj.com/site/about/guidelines.xhtml

The CM-COS and the DVA-COS will be developed to support evaluation of the impact of targeted child and/or family focussed psychosocial interventions or services, in the context of both research (randomised and non-randomised studies) and practice (service evaluations and monitoring). The target population for interventions is children aged less than 19 years of age with experience of (current or previous) DVA or CM. Given that many interventions aiming to improve child outcomes do so via support delivered to parents or multiple family members (rather than directly to the child),[7,8,22] the target group also includes parents or families of children experiencing CM or DVA. We use a definition of Psychosocial Interventions set out by the Institute of Medicine. [23] Interventions within the scope of this study include psychotherapies (e.g. cognitive-behavioural therapy), community-based treatments, family/systemic therapy, vocational rehabilitation, peer support services, integrated care interventions, and out-of-home care (i.e. foster care or adoption). Interventions may be delivered in one or more contexts (e.g. clinic, school, community). Interventions may be individual, dyad or group based, or a combination, and delivered to children with or without their parents, to parents alone, to family groups, or some combination. To be in scope an intervention must implicitly or explicitly aim to improve child outcomes by one or more of the following mechanisms: i) reducing the risk of CM/DVA occurring/reoccurring in the family; ii) improving parental (non-harming and/or harming) functioning as an indirect route to improving child outcomes; iii) limiting or preventing poor mental health, reduced wellbeing or function in children following exposure; iv) promoting children's recovery following experience of CM or DVA – here we relate to the recovery model definition which emphasises perceptions of resilience, self-identity, a sense of empowerment, hope and optimism. (e.g. [24]) Universal and targeted structural interventions are not in scope.

Study design

The study is being undertaken in two stages (see Figure 1). The first stage is underway and seeks to identify candidate outcome areas, domains and indicators. Multiple methods are being used to identify items for the candidate list including rapid evidence reviews, consultation with key stakeholders and qualitative interviews. Data will be synthesised to produce a taxonomy of outcomes, from which the two candidate lists of indicators (structured by area and domain) will be produced.

The second stage, due to begin in April 2021, will incorporate an adapted two round E-Delphi study and consensus meeting, with the aim of building agreement between different stakeholder groups regarding important outcomes. The E-Delphi technique is an iterative, multistage, online process designed to seek opinion from and develop consensus among a defined group of individuals (panel).

For peer review only - http://bmjopen.bmj.com/site/about/guidelines.xhtml

The method is frequently used when evidence in an area is known to be limited or contradictory and is widely used in health and social care research. Key features include (1) an anonymous survey process, whereby a panel (or multiple panels) of experts (by profession and/or experience) use a questionnaire to rate a series of statements over a number of rounds; (2) the provision of structured feedback to panel members between rounds with the ability to adjust ratings in light of knowledge about the group opinion and (3) anonymity for panel members during the process.[25]These features can facilitate the convergence of opinion across rounds, helping to build consensus while at the same time highlighting areas of continuing disagreement. This method has been used extensively in the context of core outcomes research [16,26,27].

We will recruit three panels for participation in the E-Delphi study to ensure that each stakeholder group is equally represented in the final consensus. [28] In a further effort to ensure the views of those with lived experience remain a central focus during this exercise, the E-Delphi method will be adapted so that in addition to feedback about their individual and own panel scores for each item, professional and researcher panels will also receive feedback about the scores of the lived experience panel. This adaptation is informed by evidence that feedback of patient scores to clinicians results in an expanded set of consensus items that better reflect the priorities of patients. [29] Additional feedback will not be given to the lived experience panel, so as to minimise the possibility of perceived power differentials influencing this panel's ratings. [28] A final face to face consensus meeting will be used to review and verify findings from the E-Delphi study, clarify any remaining uncertainty, and ratify the final core outcome set.

[Figure 1 about here]

Study Oversight

A steering committee including practitioners, policy makers and researchers representing CM and DVA fields has been formed and will meet formally twice a year.

Patient and Public Involvement

Three public advisory groups are also overseeing and consulting on the study. One group is comprised of individuals with lived experience of DVA and one of care experienced young people. These groups have been formed in partnership with relevant survivor led organisations. A third group is comprised of young people affiliated to the National Children's Bureau who are consulting more broadly on the work of the Children and Families Policy Research Unit. Partner organisations are funded to organise three meetings per year and to provide appropriate remuneration to participants. Additional funds will be paid to cover scheduled review activities organised with

For peer review only

For peer review only - http://bmjopen.bmj.com/site/about/guidelines.xhtml

partner agencies via email. Members of advisory groups will be involved in all aspects of the study including the development of the outcomes taxonomy, development of the list of candidate indicators, preparation of materials for the E-delphi and dissemination of results.

Participants

Workshops (Phase 1*)*: We will invite 30-40 individuals to attend each workshop, the aim of which will be to discuss definitions of CM/DVA and outcomes perceived to be important for survivors. Relevant researchers (mainly UK) and professionals from each field (e.g. support workers, primary and secondary health practitioners, education staff, local authority commissioners, local and national policy makers) will be identified from the research team's networks, authorship of key publications, and internet searches.

People with lived experience of CM/DVA will be approached via gatekeeper organisations and existing survivor/researcher networks known to the research team. Concerted effort will be made to invite individuals representing groups known to be marginalised from services or research on DVA/CM, or who receive inadequate service responses owing to discrimination or lack of service differentiation (i.e. assuming all groups require the same response). [30–33]

Semi-structured interviews (Phase 1*)*: We will recruit a sample of approximately 5 adults who identify as survivors of CM or exposure to DVA during childhood, and 5 parents of children currently aged 0-18 with lived experience of DVA/CM. In the first instance we will seek to recruit participants via gatekeeper organisations (see procedure below), although if recruitment is insufficient, we will seek approval for direct recruitment via social and print media. To take part in interviews, participants will be required to self-identify as having experienced CM/DVA, or as having a child who has experienced CM/DVA.

Adapted international E-Delphi study (Phase 2): Three separate panels will be recruited to take part in the consensus study comprising: i) individuals with lived experience (parents of children with experience of CM/DVA and adults experiencing abuse in childhood); ii) frontline and strategic professionals involved in the delivery and commissioning of CM/DVA services and related policy; iii) researchers. The first two panels will include members from the UK, with the researcher panel including international researchers from high, middle- and low-income countries. We will aim to recruit 30 individuals to each panel.

Survivors and professionals taking part in the workshops and semi-structured interviews described above (and who give consent for further contact) will be approached for participation in the lived experience and professional panels respectively. If needed, additional participants will be recruited

For peer review only - http://bmjopen.bmj.com/site/about/guidelines.xhtml

through key organisations working with either CM or DVA survivors and snowball sampling. Key researchers, with at least one peer-reviewed publication from either the CM/DVA field, will be identified through the rapid reviews, researcher networks, participation in workshops, and via the expert panel. For all panels, participants must be able to read and understand English in order to participate.

Consensus workshop following E-Delphi study (Phase 2): A face-to-face consensus meeting, with a purposively sampled panel (n=30) representing all key stakeholder groups, will be recruited from participants taking part in earlier phases of the study. Individuals outside of the study will be approached as needed to ensure balanced representation and inclusion of individuals of strategic importance to take up and implementation of study findings. Appropriate amendments to ethical approvals will be sought to accommodate this.

Procedure

Phase 1

Rapid reviews

We will conduct a series of rapid reviews using systematic methods (see supplementary appendices for protocols and review questions). We will review experimental and quasi-experimental intervention studies (international), qualitative studies containing primary accounts of experience of relevant interventions or outcomes that are sought by families and children experiencing CM/DVA (international), and grey (UK) literature reporting descriptions of interventions, service evaluations or consultation regarding appropriate outcomes across the DVA and CM fields.

We will search a range of relevant databases and websites under the guidance of an expert librarian. Following rapid review techniques [34,35] we will search since 2014 for intervention studies (covering the time elapsed since previous key reviews, [8,36]) and 2014/5 for the qualitative studies to build on recent qualitative reviews. [37] The grey literature review will primarily focus on searches of relevant UK organisation websites and will include any service or intervention evaluation or any consultation or review, to identify relevant candidate outcomes or outcome tools for use in the context of service delivery or evaluation.

A second reviewer will screen and extract data from a minimum of 5% of titles/abstracts and articles to ensure consistency. Inter-rater reliability kappa scores will be calculated, and disagreements resolved through discussion (or a third reviewer if necessary) throughout the process. Relevant

For peer review only - http://bmjopen.bmj.com/site/about/guidelines.xhtml

outcome indicators will be extracted, as well as their measurement instruments where possible. There will be no appraisal of study quality and outcomes will be extracted from all identified papers.

Stakeholder workshops

We will hold two invite-only workshops (one focussed on CM and one focussed on DVA) to gather stakeholder views. The purpose of these events will be to i) explore definitional issues, specifically how each phenomenon is defined by particular groups and the function that this definition plays in practice (in terms of enabling access to services/interventions and measuring change), and ii) to explore outcomes perceived to be important indicators of benefit or harm for children and families experiencing CM/DVA.

Participants will be seated on tables of 6-8. Each table will include at least two individuals with lived experience and one facilitator. Guided by facilitators, participants will be asked to generate ideas relating to desirable (or undesirable) outcomes, unconstrained by what they believe to be measurable or achieved via currently available interventions. This will be an attempt to ensure output is not merely reflective of current practice or discourse. Designated scribes will take notes throughout the day, which will be collated and analysed thematically. [38] Participants in the workshops will be asked for permission to contact them at a later date for the purpose of inviting them to participate in the international E-Delphi study.

Interviews with individuals with lived experience of DVA/CM as a child or as parent of a child

Participants will be identified via key gatekeeper organisations (where work with survivors of CM/DVA is core business) contacted for the purpose of workshop participation (see above). Participants will be approached directly by a professional from the gatekeeper organisation or they will receive an open invitation circulated through the organisation’s survivor network. Where participants are approached by professionals, they will be given brief information about the study and asked for permission to pass contact details to the research team. Individuals responding to an open invitation will be asked to contact a member of the research team directly. They will be assured of the anonymity of their involvement.

Basic socio-demographic information and minimal information about experiences of CM or DVA will be collected via questionnaire prior to the interview and will be used for sample description. Participants will have the opportunity to take part in the interview face-to-face, by video call or by phone, according to their personal preferences and public health guidance on social distancing. For those participants who wish to take part but are unable to speak directly to interviewers, they will

For peer review only - http://bmjopen.bmj.com/site/about/guidelines.xhtml

be able to answer the interview questions by email. [39] Interview schedules will be used to guide interviews, which will be recorded and transcribed verbatim and analysed thematically. [38]

Outcome generation

A list of candidate outcome areas (e.g. health and wellbeing), domains (e.g. mental health) and specific indicators (e.g. withdrawal from friends and activities) will be generated iteratively by the research team, drawing on all information sources described above. An unedited candidate list of outcome indicators generated from stakeholder workshops will be used as a starting point. Identification of duplicate and overlapping outcome indicators from the list will be undertaken in parallel by two team members (CP, EH). Similar items will be dropped or combined to produce a reduced inventory. Disagreements between team members will be resolved through discussion. All suggestions to drop or combine items will be reviewed by two further research team members (RG, GF) and survivor involvement groups. Similar indicators (i.e., outcomes that could be compared across studies or combined in a meta-analysis [21]) will be grouped into outcome domains by two team members, and reviewed by two further members of the research team and survivor involvement groups. Simultaneously, a taxonomy to organise domains into broader outcome areas will be developed. Here we will draw on existing practical and theoretical frameworks to categorise health outcomes, [40] as well as the aetiology and impacts of DVA and CM. [41–44] This overarching framework to describe the hierarchical structure of outcomes identified in workshops will be reviewed and refined by all members of the research team, the expert advisory group, and survivor involvement groups.

A candidate list of outcome indicators from the rapid reviews will be generated and de-duplicated (CP, EH). Four research team members and at least two survivor representatives will, in parallel, attempt to categorise indicators using the developed taxonomy. Categorisations will be compared, disagreements discussed, and consensus reached through discussion. New domains or areas will be added where necessary. Unique indicators (not already included) will be identified from the candidate list generated from the reviews and added to the taxonomy. This iterative process will be repeated with data yielded from interviews.

The final taxonomy and labelling of terms will be reviewed by the advisory group, and all three public involvement groups. Particular attention will be given to the language used to describe outcome areas, domains, and specific indicators to ensure they are understandable, meaningful and acceptable to all stakeholder groups. Further refinement (including addition of areas, domains or indicators) will be undertaken following this review. The final step in the process will be to examine outcomes against a priori criteria designed to ensure the final COS has maximum utility. These

For peer review only - http://bmjopen.bmj.com/site/about/guidelines.xhtml

include: i) the extent to which the outcome indicator relates to children's feelings, function or survival, or the process of delivering services to survivors ii) whether the outcome is 'changeable' iii) and whether the outcome indicator could feasibly change as a result of a psychosocial intervention – here we will draw on literature elucidating mechanisms through which exposure to violence and abuse may be communicated to child outcomes. (e.g. [45]) Four members of the research team, at least two members of the expert advisory group, and four members of the survivor involvement groups (with equal representation of CM and DVA experience) will independently assess outcome indicators against the criteria listed above. Any indicators identified as not meeting all criteria by one or more reviewers will be discussed and a majority decision taken to exclude or include it in the candidate list. Excluded outcomes will be reported in the final paper, along with reasons for exclusion. Where needed a glossary of terms and explanatory text will be developed to aid clarity for participants in the E-Delphi study.

Phase 2

Adapted international -Delphi study

A sequential two-round, three-panel E-Delphi study will be conducted.

Round 1*:* A questionnaire for use in the E- Delphi study will be developed using the taxonomy described above. Areas and domains will serve as headings and sub-headings by which to organise the survey, so as to encourage completion and to allow us to explore the relative importance of indicators within the same domain. The questionnaire will be reviewed by advisory and involvement groups and refined in line with feedback. Ethical approval will be sought as an amendment to that granted for phase one of the study.

Participants will be contacted by email to remind them about the COS study and their attendance at a previous workshop (if appropriate) and to invite them to participate in the E-Delphi study. A second email containing the information sheet and link to an online questionnaire will be sent one-two days after the initial contact. Participants will be required to indicate that they have read the information sheet and agree to take part, before proceeding to the questionnaire. The questionnaire will be administered via Qualtrics (https://www.qualtrics.com/uk/)hosted by the University College London.

Participants will be presented with a list of outcome indicators organised by area and outcome domain. They will be asked to rate each outcome presented, on a nice-point scale of importance (1=not at all important, 9=extremely important). Participants will also be given the opportunity to add any additional outcomes that are missing from each domain using a free text comments box.

For peer review only

For peer review only - http://bmjopen.bmj.com/site/about/guidelines.xhtml

During this round we will also collect demographic data including ethnicity, age, gender, profession and country of professional operation. The questionnaire will remain open for 14 days and reminder emails will be sent out seven and two working days before closure.

Item level descriptive statistics will be generated for each panel and item including: number of respondents, minimum and maximum values, measures of central tendency and dispersion. Criteria for item inclusion in round two will be an item is rated seven-nine(on a 9-point Likert scale) by 50% or more participants in at least one panel and one-three by no more than 15% of participants in any stakeholder group. [46]This low threshold for inclusion enables us to reduce response burden in round two by dropping unimportant items given higher number of items are associated with significantly lower response rates in COS Delphi surveys, [47] while also reducing the likelihood of dropping outcomes that may have been rated more highly in subsequent rounds had participants been given feedback on them. New items will be included if two or more panellists suggest inclusion, and the research team deem it unique to existing content. [15] Panellists completing Round one will be invited to participate in Round two if they rated ≥50% of survey items. Non-completers will not be contacted for participation in round two. We will assess attrition rates for each panel and by demographic profiles.

Round two*:* An amendment to the existing approval will be sought for use of the shorter round two questionnaire. The same items will be included in questionnaires issued to each panel. Each panel member will receive a personalised questionnaire reporting panel averages and their own rating for each item. As noted above, professional and researcher panels will also receive feedback about the ratings of the survivor panel. Panellists will be asked to re-rate each of the included items, and rate for the first time, any new outcomes put forward in round one. All new outcomes suggested in round one (irrespective of the panel from which they derived) will be presented to each of the three panels.

As before, participants will receive two reminders to complete the questionnaire, over the course of 14 days. Following completion of the study, descriptive statistics will be computed. Items will be deemed important to a particular panel if they are rated seven -nine by ≥70% of respondents and one - three ≤15% by the panel. Conversely, items will be classified as unimportant to a group if ≥70% of respondents rate it as one-three and ≤15% rate it as seven-nine. Any items not classified as important or unimportant will be deemed not to have reached consensus. Items will be considered ‘core’ and recommended for inclusion in the COS if they are rated as important by all three panels. We will assess the impact of attrition on consensus by comparing (within panels) the mean total

For peer review only

For peer review only - http://bmjopen.bmj.com/site/about/guidelines.xhtml

item scores for those completing round 1 only and those completing both rounds; we will also compare the average scores for completers vs non completers by each item (within panel).[15]

Consensus meeting

A face-to-face consensus meeting, with a purposively sampled panel (n=30) representing all key stakeholder groups, will be held to discuss, vote and agree on the final CM- and DVA COS. The format of the meeting will follow the process set out by the James Lind Alliance (JLA) final priority setting workshops http://www.jla.nihr.ac.uk/jla-guidebook/chapter-8/workshop-process-on-the-day.htm This method is pertinent given that JLA priority setting meetings involve multiple stakeholders, discussion of interim results derived from the ranking of evidence uncertainties, and production of a 'top ten'.

Whilst there is no recommended maximum number of outcomes that should be included in a COS, for it to be pragmatic we aim to arrive at a maximum of 10 outcomes. [48,49] The JLA priority setting method involves a structured process including small group and whole group discussion, ranking and reranking. The method will be adapted to include a preliminary step, where participants review those outcomes identified as important to the lived experience panel, but which didn't reach consensus across all groups. Participants will be asked to identify any outcomes that should be discussed in the workshop, alongside outcomes meeting the consensus definition. This initial step is an attempt to ensure appropriate weight is given to the voice of those with lived experience of DVA/CM. During discussion, workshop participants will be asked to take into consideration the extent to which identified outcomes are 'changeable', and could be feasibly impacted by psycho-social interventions. The final COS and also a list of all items reaching consensus will be published.

ETHICS AND DISSEMINATION

Ethical approval

Ethical approval was sought from the Research Ethics committee at University College London. At all stages of the study we will obtain written consent for contact information relating to potential participants to be passed via gatekeeper organisations assisting with recruitment. We will obtain written informed consent from participants in interviews and the consensus meeting. Online consent will be obtained from participants when they opt in to participate in the E-Delphi study, before they are able to proceed.

Dissemination and implementation

For peer review only - http://bmjopen.bmj.com/site/about/guidelines.xhtml

We have registered the study on the COMET website. We will provide tailored briefings to UK policy makers, think tanks, commissioners and third sector organisations whilst the study is in progress as well as completed. This will maximise interest and intention to use the core outcome sets. We also intend to use these briefings as a vehicle for recruitment to the E-Delphi study. We will involve the leads of international scholarly networks in workshops and recruit member networks to the E-Delphi study

We will disseminate our findings through peer reviewed and open access publications, the COMET website, and presentations at international conferences. We will engage with journal editors and funding agencies and the relevant Cochrane and Campbell review groups to promote awareness of the CM- and DVA-COS. We will provide briefings and links to publications to international research and policy networks, for dissemination through the networks of the VAMHN membership and CPRU collaborators, as well as the wider network of NIHR Policy Research Units, Applied Research Collaborations (ARCs) and UKRI networks. We will invite survivors who participated in workshops and in involvement groups to co-produce plain-language, service-user facing communication materials for circulation in places where survivors access support (formal or informal). We will also develop tailored briefings to enable findings to be shared with all study participants; participation in this type of study is known to be a key facilitator of implementation.[15] Briefings will be published on the CPRU website and emailed to all third sector organisations working specifically with survivors of CM and DVA, as well as Local Authority commissioners and CCGs.

A high level review of the reach and uptake of the core outcome sets will be undertaken in 2023. One of the key issues for review will be whether the core outcome set has become aligned or adopted by research and practice networks or collaborations ,and recognised by funders (e.g. NIHR) and bodies co-ordinating health and social care intervention research and systematic reviews (e.g. Cochrane and Campbell Collaborations)

DISCUSSION

Currently no published COS exists for evaluation of services and interventions to improve child outcomes following experience of CM or DVA. It is essential that outcomes measured in the context of trials and practice based research reflect the benefits (and harms) sought and prioritised by those who use, deliver and commission DVA and CM programmes, as well as those who research them. A COS that is developed with strong participation from people with lived experience of CM or DVA and those working to support them will help to ensure that relevant outcomes are measured in all evaluative studies. This in turn will enhance consistency across studies and the quality and value of

For peer review only - http://bmjopen.bmj.com/site/about/guidelines.xhtml

research. High levels of awareness and uptake of this study's outputs is critical to achieving its ultimate aim.

Limitations

The design of this study is limited by the lack of direct involvement of children and young people in either qualitative interviews or the E-Delphi study. Given the study described here represents meta-research, it was felt that potential risks to children could not be justified. Their voices are nonetheless to some extent reflected through the broad reviews of evidence and inclusion of parent perspectives. It is also beyond the means of the study to involve survivors and service providers from LMICs, although we will include research from LMICs in the evidence reviews and actively recruit researchers from or researching LMIC settings.

For peer review only

REFERENCES

1 Evans SE, Davies C, DiLillo D. Exposure to domestic violence: A meta-analysis of child and adolescent outcomes. *Aggress Violent Behav* 2008;**13**:131–40.

2 Gilbert R, Widom CS, Browne K, *et al.* Burden and consequences of child maltreatment in high-income countries. Lancet. 2009;**373**:68–81. doi:10.1016/S0140-6736(08)61706-7

3 MacMillan HL, Wathen CN, Barlow J, *et al.* Interventions to prevent child maltreatment and associated impairment. *Lancet* 2009;**373**:250–66. doi:10.1016/S0140-6736(08)61708-0

4 McTavish JR, Kimber M, Devries K, *et al.* Children's and caregivers' perspectives about mandatory reporting of child maltreatment: a meta-synthesis of qualitative studies. *BMJ Open* 2019;**9**:e025741. doi:10.1136/bmjopen-2018-025741

5 Lewis N V, Feder GS, Howarth E, *et al.* Identification and initial response to children's exposure to intimate partner violence: a qualitative synthesis of the perspectives of children, mothers and professionals. *BMJ Open* 2018;**8**:e019761. doi:10.1136/bmjopen-2017-019761

6 Home Office. Domestic Abuse Bill 2020: overarching factsheet. London: 2020. https://www.gov.uk/government/publications/domestic-abuse-bill-2020-factsheets/domestic-abuse-bill-2020-overarching-factsheet

7 Howarth E, Moore THM, Welton NJ, *et al.* Improving outcomes for children exposed to domestic violence (IMPROVE): An evidence synthesis. *Public Heal Res* 2016.

8 Macdonald G, Livingstone N, Hanratty J, *et al.* The effectiveness, acceptability and cost-effectiveness of psychosocial interventions for maltreated children and adolescents: An evidence synthesis. *Health Technol Assess (Rockv)* 2016;**20**. doi:10.3310/hta20690

9 Latzman NE, Casanueva C, Brinton J, *et al.* The promotion of well-being among children exposed to intimate partner violence: A systematic review of interventions. *Campbell Syst Rev* 2019;**15**. doi:10.1002/cl2.1049

10 Clarke M. Standardising outcomes for clinical trials and systematic reviews. *Trials* 2007;**8**:1–3.

For peer review only - http://bmjopen.bmj.com/site/about/guidelines.xhtml

doi:10.1186/1745-6215-8-39

11 Clarke M, Williamson PR. Core outcome sets and systematic reviews. *Syst Rev* 2016;**5**:11. doi:10.1186/s13643-016-0188-6

12 Howarth E, Moore THM, Shaw ARG, *et al.* The Effectiveness of Targeted Interventions for Children Exposed to Domestic Violence: Measuring Success in Ways that Matter to Children, Parents and Professionals. *Child Abus Rev* 2015;**24**:297–310. doi:10.1002/car.2408

13 Hoagwood KE, Jensen PS, Acri MC, *et al.* Outcome domains in child mental health research since 1996: have they changed and why does it matter? *J Am Acad Child Adolesc Psychiatry* 2012;**51**:1241-1260.e2. doi:10.1016/j.jaac.2012.09.004

14 Howarth E, Moore THM, Shaw ARG, *et al.* The Effectiveness of Targeted Interventions for Children Exposed to Domestic Violence: Measuring Success in Ways that Matter to Children, Parents and Professionals. *Child Abus Rev* 2015;**24**:297–310. doi:10.1002/car.2408

15 Williamson PR, Altman DG, Bagley H, *et al.* The COMET Handbook: version 1.0. *Trials* 2017;**18**:280. doi:10.1186/s13063-017-1978-4

16 Davis K, Gorst SL, Harman N, *et al.* Choosing important health outcomes for comparative effectiveness research: An updated systematic review and involvement of low and middle income countries. *PLoS One* 2018;**13**. doi:10.1371/journal.pone.0190695

17 Gorst SL, Gargon E, Clarke M, *et al.* Choosing important health outcomes for comparative effectiveness research: An updated review and user survey. *PLoS One* 2016;**11**. doi:10.1371/journal.pone.0146444

18 Hamby S, Finkelhor D, Turner H, *et al.* The overlap of witnessing partner violence with child maltreatment and other victimizations in a nationally representative survey of youth. *Child Abuse Negl* 2010;**34**:734–41. doi:http://dx.doi.org/10.1016/j.chiabu.2010.03.001

19 Henry C. Exposure to domestic violence as abuse and neglect: Constructions of child maltreatment in daily practice. *Child Abus Negl* 2018;**86**:79–88. doi:10.1016/j.chiabu.2018.08.018

20 Lawson J. Domestic violence as child maltreatment: Differential risks and outcomes among cases referred to child welfare agencies for domestic violence exposure. *Child Youth Serv Rev* 2019;**98**:32–41. doi:10.1016/j.childyouth.2018.12.017

21 Kirkham JJ, Gorst S, Altman DG, *et al.* Core Outcome Set-STAndardised Protocol Items: the

For peer review only

For peer review only - http://bmjopen.bmj.com/site/about/guidelines.xhtml

COS-STAP Statement. *Trials* 2019;**20**:116. doi:10.1186/s13063-019-3230-x

22 Woodman J, Simon A, Hauari H, *et al.* A scoping review of ‘think-family’ approaches in healthcare settings. *J Public Health (Bangkok)* Published Online First: 22 January 2019. doi:10.1093/pubmed/fdy210

23 England MJ, Butler AS, Gonzalez ML, *et al. Psychosocial interventions for mental and substance use disorders : a framework for establishing evidence-based standards*.

24 Leamy M, Bird V, Le Boutillier C, *et al.* Conceptual framework for personal recovery in mental health: Systematic review and narrative synthesis. Br. J. Psychiatry. 2011;**199**:445–52. doi:10.1192/bjp.bp.110.083733

25 Jorm AF. Using the Delphi expert consensus method in mental health research. *Aust New Zeal J Psychiatry* 2015;**49**:887–97. doi:10.1177/0004867415600891

26 Gargon E, Gurung B, Medley N, *et al.* Choosing important health outcomes for comparative effectiveness research: A systematic review. *PLoS One* 2014;**9**. doi:10.1371/journal.pone.0099111

27 Sinha I, Jones L, Smyth RL, *et al.* A systematic review of studies that aim to determine which outcomes to measure in clinical trials in children. *PLoS Med* 2008;**5**:0569–78. doi:10.1371/journal.pmed.0050096

28 Sinha IP, Smyth RL, Williamson PR. Using the Delphi technique to determine which outcomes to measure in clinical trials: recommendations for the future based on a systematic review of existing studies. *PLoS Med* 2011;**8**:e1000393. doi:10.1371/journal.pmed.1000393

29 Macefield R, Blencowe N, Brookes S, *et al.* Core outcome set development: the effect of Delphi panel composition and feedback on prioritisation of outcomes. *Trials* 2013;**14**:P77. doi:10.1186/1745-6215-14-S1-P77

30 Thiara RK, Hague G, Mullender A. Losing out on both counts: Disabled women and domestic violence. *Disabil Soc* 2011;**26**:757–71. doi:10.1080/09687599.2011.602867

31 Anitha S. No Recourse, No Support: State Policy and Practice Towards South Asian Women Facing Domestic Violence in the UK. 2010. doi:bcn160

32 Douglas H, Walsh T. Mandatory Reporting of Child Abuse and Marginalised Families. Springer, Dordrecht 2015. 491–509. doi:10.1007/978-94-017-9685-9_23

33 Brophy J. Child maltreatment in diverse households: Challenges to law, theory, and practice. *J*

For peer review only

For peer review only - http://bmjopen.bmj.com/site/about/guidelines.xhtml

Law Soc 2008;**35**:75–94. doi:10.1111/j.1467-6478.2008.00415.x

34 Ganann R, Ciliska D, Thomas H. Expediting systematic reviews: Methods and implications of rapid reviews. *Implement Sci* 2010;**5**:56. doi:10.1186/1748-5908-5-56

35 Schünemann HJ, Moja L. Reviews: Rapid! Rapid! Rapid!.and systematic. Syst. Rev. 2015;**4**:4. doi:10.1186/2046-4053-4-4

36 Howarth E, Moore THM, Welton NJ, *et al.* Improving outcomes for children exposed to domestic violence (IMPROVE): An evidence synthesis. *Public Heal Res* 2016;**4**. doi:10.3310/phr04100

37 Arai L, Heawood A, Feder G, *et al.* Hope, Agency, and the Lived Experience of Violence: A Qualitative Systematic Review of Children's Perspectives on Domestic Violence and Abuse. *Trauma, Violence, Abus* 2019;:152483801984958. doi:10.1177/1524838019849582

38 Braun V, Clarke V. Using thematic analysis in psychology. *Qual Res Psychol* 2006;**3**:77–101.

39 Braun V, Clarke V, Boulton E, *et al.* The online survey as a *qualitative* research tool. *Int J Soc Res Methodol* 2020;:1–14. doi:10.1080/13645579.2020.1805550

40 Dodd S, Clarke M, Becker L, *et al.* A taxonomy has been developed for outcomes in medical research to help improve knowledge discovery. *J Clin Epidemiol* 2018;**96**:84–92. doi:10.1016/J.JCLINEPI.2017.12.020

41 Heise LL. What works to prevent partner violence. *Dfid* 2011;:1–110. doi:978 0 902657 85 2

42 Heise LL. Violence against women: An integrated, ecological framework. *Violence Against Women* 1998;**4**:262–90. doi:10.1177/1077801298004003002

43 Belsky J. Etiology of child maltreatment: A developmental-ecological analysis. *Psychol Bull* 1993;**114**:413–34. doi:10.1037/0033-2909.114.3.413

44 van IJzendoorn MH, Bakermans-Kranenburg MJ, Coughlan B, *et al.* Annual Research Review: Umbrella synthesis of meta-analyses on child maltreatment antecedents and interventions: differential susceptibility perspective on risk and resilience. J. Child Psychol. Psychiatry Allied Discip. 2020;**61**:272–90. doi:10.1111/jcpp.13147

45 Cameranesi M, Piotrowski CC. Critical review of theoretical frameworks elucidating the mechanisms accounting for the adverse developmental outcomes observed in children following exposure to intimate partner violence. Aggress. Violent Behav. 2020;**55**:101455. doi:10.1016/j.avb.2020.101455

For peer review only

For peer review only - http://bmjopen.bmj.com/site/about/guidelines.xhtml

46 Blazeby JM, Macefield R, Blencowe NS, *et al.* Core information set for oesophageal cancer surgery. *Br J Surg* 2015;**102**:936–43. doi:10.1002/bjs.9840

47 Gargon E, Crew R, Burnside G, *et al.* Higher number of items associated with significantly lower response rates in COS Delphi surveys. *J Clin Epidemiol* 2019;**108**:110–20. doi:10.1016/j.jclinepi.2018.12.010

48 Sherratt FC, Eaton S, Walker E, *et al.* Development of a core outcome set to determine the overall treatment success of acute uncomplicated appendicitis in children: A study protocol. *BMJ Paediatr Open* 2017;**1**:e000151. doi:10.1136/bmjpo-2017-000151

49 Knaapen M, Hall NJ, Van Der Lee JH, *et al.* Establishing a core outcome set for treatment of uncomplicated appendicitis in children: Study protocol for an international Delphi survey. *BMJ Open* 2019;**9**:e028861. doi:10.1136/bmjopen-2018-028861

For peer review only

LEGEND

Figure 1: Study design

ACKNOWLEDGEMENTS

First and foremost we thank the survivors and other members of the public who contributed to this study. Survivor involvement is facilitated by VOICES, a Survivor led charity for women who have experienced domestic abuse. We also extend our thanks to members of our advisory groups which have informed the development of the study design and have commented on drafts of this manuscript. Members of the professional advisory group are as follows: Elaine Fulton, Dr Deborah Hodes, Dr Carol Rivas, Professor Sally Kendall, David and Elisabeth Carney Haworth and Hannah Edwards.

AUTHOR CONTRIBUTIONS

EH conceived of the original study design, which was refined and developed by EH, CP, RG, and GF, JW and EW. CP and EW led the development of the public patient involvement strategy. CP and HC developed protocols for rapid reviews, which were reviewed and refined by CP, EH, RG, JW and GF. CP undertook all searches; CP and EH performed data extraction for reviews. EH, CP, RG, JW, EW, and GF contributed to the writing and review of the protocol paper.

FUNDING STATEMENT

This study is funded by the National Institute for Health Research (NIHR) Policy Research Programme. Funder reference: PR-PRU-1217-21301; UCL award code: 177763 The views expressed

are those of the author(s) and not necessarily those of the NIHR or the Department of Health and Social Care.

COMPETING INTERESTS

We have read and understood BMJ policy on declaration of interests and declare that we have no competing interests.

DATA SHARING STATEMENT

Please contact the corresponding author or unit manager cpru.data@ucl.ac.uk with enquiries about the data used in this study.

SUPPLEMENTARY MATERIAL

Rapid review protocols

For peer review only

Figure 1: Study design

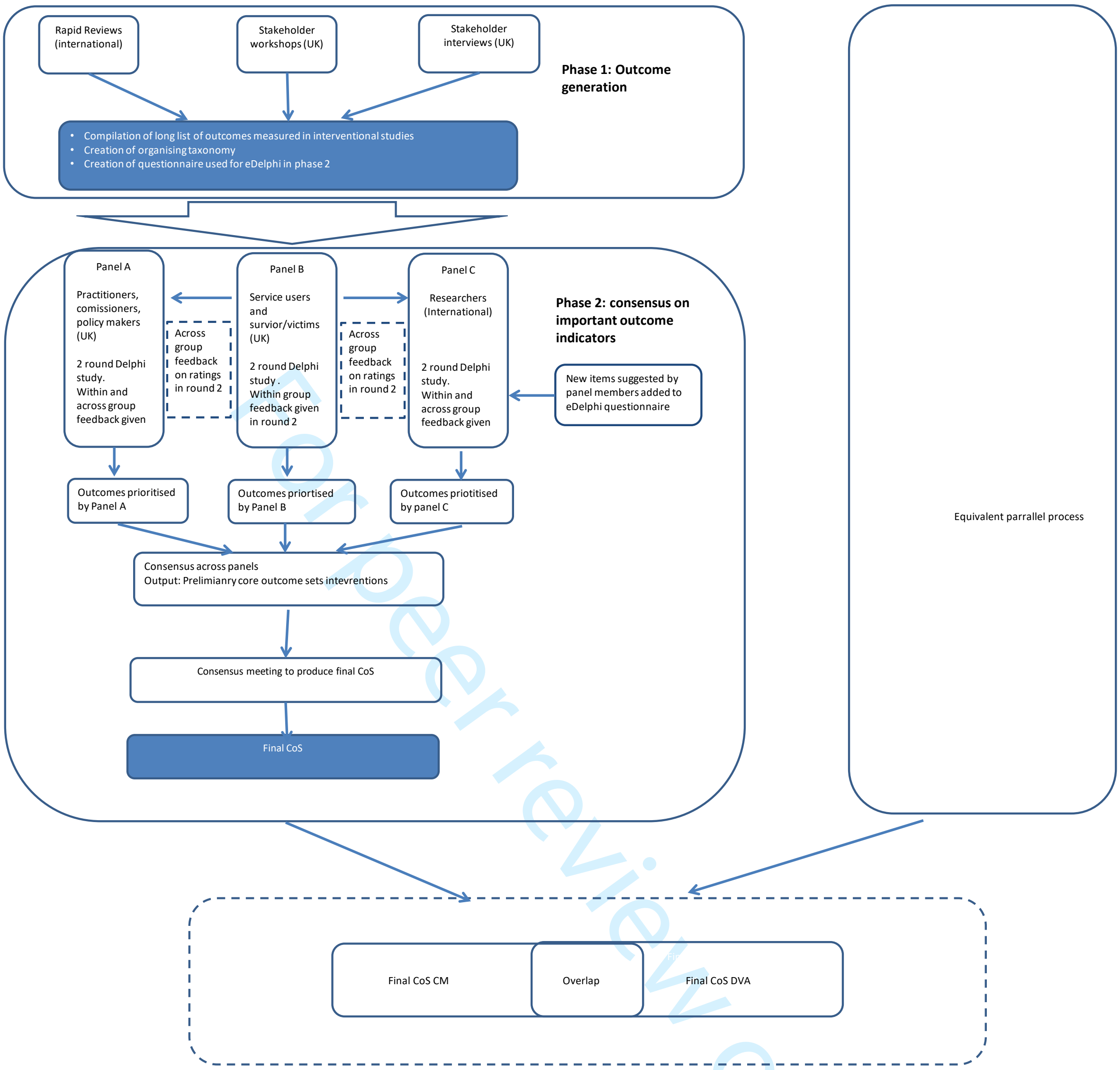

For peer review only

Supplementary Appendices – Rapid Review Protocols

A - Rapid review of systematic reviews of intervention studies

Review question: How are outcomes defined and measured in controlled trials of interventions aiming to improve outcomes of children and families with children exposed to DVA/CM and those aiming to reduce subsequent abusive behaviour by perpetrators of DVA/CM?

a. This includes the definition and measurement of DVA/CM.

This rapid review will be carried out in two steps: firstly searches for systematic reviews (SR) will be carried out, then these reviews will be used to extract individual studies which will be screened for relevance. This process will be carried out in parallel for the DVA and CM literature.

Study inclusion: Peer-reviewed systematic reviews of controlled or quasi experimental comparator intervention studies: with or without randomisation.

The DARE criteria for SRs are at least 4 of the following: reporting of inclusion/exclusion criteria; adequate search; synthesis of included studies; quality assessment of studies; sufficient detail presented (CRD, 1995). For the purposes of this review, SRs will be included if they use an electronic database and have a structured search strategy.

- Published since 2014.
- No restrictions by country. English language only.

- Individual studies must include DVA/CM in one of the following ways:
  - Entry to the intervention is determined by experience, perpetration or identified as at risk of DVA/CM. (Identification of risk is by researchers, practitioners or participants thus we do not have a definition)
  - Subgroup analysis is carried out of participants who have experienced (or are considered to be at risk of) DVA/CM
  - DVA/CM is measured as an exposure (this could be retro or prospectively reported)

Exclusion: Non peer-reviewed studies, qualitative studies, general literature reviews, protocols, case reports, cross-sectional studies, general discussion papers, letters, commentaries, book chapters, conference papers, theses and dissertations.

Population inclusion: children or families with children at risk of experiencing, or experiencing DVA/CM'. This includes unborn children, children (aged 0 to 18 years), designated as victim or witness. For DVA any adult family members who have a parenting role (Early Intervention Foundation, 2014), whether designated as perpetrator, victim, witness or household member. For CM any adult family members who have a caring role, whether designated as maltreating parent, witness or household member. These adults and children could either be the primary study population of interest or form a subgroup in a wider study population.

Intervention inclusion: Any interventions or services where:

For peer review only - http://bmjopen.bmj.com/site/about/guidelines.xhtml

- Experience of or increased risk of experiencing DVA/CM is a criterion for being offered the service
  OR
- DVA/CM is measured as an exposure or outcome of interest
  AND
- At least one child or family-level outcome is measured. Family-level outcomes do not need to be explicitly labelled as 'family' level, we will make a judgement. However, they include any outcome that affects the family/household unit. For example, worklessness in study where at least some participants are reported to be parents would be included.

- Studies must include evaluation of a defined activity/programme and evaluation of a hypothesised effect
- Interventions may be delivered to any family member(s) as an individual or in a group. Any duration of intervention will be included. Any setting will be considered.

Exclusion: universal interventions that do not specifically target children and families at risk of DVA/CM; targeted interventions that do not measure any child or family level outcomes e.g. perpetrator programmes that focus solely on attitudinal change; DVA (only) interventions focused solely on elder abuse, sibling abuse or child perpetration of domestic violence where participants have not been identified as exposed to DVA.

Comparator inclusion: Any control or comparison group/period with participants receiving no care, treatment as usual or any other treatment.

Outcome inclusion:
- Any child outcome related to i) the child's experience of adversity ii) child functioning, including risky behaviours (see (Maclean et al., 2016) for full list of health and wellbeing outcomes).
- Any outcomes related to the quality of the caregiving environment (e.g. parenting, maternal depression, stressful life events, maternal psychological distress, parental substance misuse).
- Any outcomes related to material deprivation e.g. low income, economic hardship or stress (including perceived), social capital, hunger, food poverty, housing instability.
- Any other outcome judged to relate to children or families by the research team.
- Outcomes can be reported by professionals, child, parent or other family member and they can be retrospective or prospective.
- Outcomes can be end points, surrogate markers for end points or intermediate outcomes.
- No minimum or maximum follow-up is required.

Context inclusion: Studies from any country in any setting.

Searches
The following electronic databases will be searched from 2014: Medline, Embase, PsycInfo, Cochrane and Web of Science. Searching will include expert recommendations of relevant broader studies, including relevant parenting programmes.

The search strategy will include MeSH terms relating to DVA/CM and the BMJ systematic review strategy ((*Study Design Search Filters | BMJ Best Practice*, n.d.)). Key word terms for DVA/CM, abuse,

For peer review only - http://bmjopen.bmj.com/site/about/guidelines.xhtml

violence, family members and systematic reviews will be used. These have been developed from the two main NIHR-funded studies in the area ((Howarth et al., 2016) and (Macdonald et al., 2016)) and adapted as required for the different databases with guidance from an expert librarian.

These reviews will be carried out separately for DVA and CM. The DVA search will be run first and any CM studies that do not mention DVA will be excluded (and vice versa). As part of the review involves collecting definitions of DVA/CM, any study deemed to fit within the umbrella by the research team will be included.

Data extraction (selection and coding)
All systematic reviews identified by database searches will be downloaded to CADIMA (Kohl et al., 2018) and de-duplicated. Screening criteria will be tested by two reviewers on 200 titles/abstracts and interrater reliability assessed. Titles/abstracts will be screened by one reviewer for inclusion in full-text review. A second reviewer will independently review 10% of title/abstracts. If there is a high level of disagreement, the second reviewer will continue reviewing titles/abstracts until agreement is reached. Full-text systematic reviews will be screened for inclusion and a second reviewer will independently review 10% of these as above. Key data from the systematic reviews (e.g. definition of DVA/CM) will be extracted into CADIMA by one reviewer.

Individual studies will be extracted from the included full-text systematic reviews. These studies will be downloaded to Zotero and de-duplicated. The remaining studies will then be screened for inclusion in full-text review and data extraction. Data will be extracted into Access using a standardised form and a second researcher will review extraction from the first 5 papers. The following data will be extracted: bibliographic information, study design, setting, sample characteristics, definitions of DVA/CM, intervention details, primary and secondary outcomes (applicable for children and families) and their measures, descriptions of mechanisms. (Where DVA/CM is not measured as an outcome, nor is there a subgroup analysis, only exposure definition will be extracted.) Quality control/risk of bias will not be assessed because the aim of the review is solely to collect outcomes.

Strategy for data synthesis
Narrative synthesis and tabulation of outcomes extracted.

B - Rapid review of qualitative studies

Review questions:

1) What outcomes (benefits or harms) are sought or experienced by actual or potential recipients of interventions/services aiming to prevent or reduce the risk of harm associated with DVA/CM?

2) What outcomes (benefits or harms) are sought by stakeholders* involved in developing and/or delivering interventions to children/families experiencing DVA/CM?

**'stakeholder' is defined as in the IMPROVE study i.e. young people with experience of DVA/CM services, parents/caregivers with experience of using DVA/CM services or professionals involved in commissioning and delivering services to families affected by DVA/CM.*

For peer review only - http://bmjopen.bmj.com/site/about/guidelines.xhtml

This review will be carried out in parallel for DVA and CM.

Study inclusion:

- Primary qualitative (i.e. analysis of interviews, focus groups or other verbal analysis which is not quantified) intervention studies either as a standalone study or a discrete component of mixed-method studies.
- Direct and sufficient verbatim text from participants for analysis (i.e. more than two lines) c.f. Arai et al. (2019).
- Published since October 2015 (DVA) and July 2014 (CM) to build on Howarth et al. (2016) and Macdonald et al. (2016).
- No restrictions by country. English language only.

- Individual studies must include DVA/CM in one of the following ways:
    - Participation in the study is determined by experience, perpetration or specifically identified as at risk of DVA/CM. Participants may have received an intervention or may be discussing the impact of DVA/CM and their desired outcomes for the future. (To ensure we are not limited by outcomes defined by current interventions).

    OR
    - Stakeholders involved in developing and/or delivering interventions to children/families experiencing DVA/CM (c.f. Howarth et al, 2016, p.52), or stakeholder discussion of outcomes that are sought either in relation to an intervention or the future in general.

Exclusion: Non peer-reviewed studies, surveys or quantitative studies with descriptive free-text only, general literature reviews, case reports, general discussion papers, letters, commentaries, editorials, book chapters, conference papers, theses and dissertations.

Population inclusion: Any adult or child stakeholders relevant to DVA/CM. This could be as a result of experience, perpetration, identified as at risk, delivering, commissioning or intending to deliver services.

Phenomenon of interest: DVA/CM

Design: Any qualitative approach to data collection and analysis (e.g. interviews, focus groups)

Evaluation: Perspectives of experienced or anticipated benefits or harms of interventions, and/or desired outcomes in general related to DVA/CM.

Searches

The following electronic databases as advised for qualitative research (Evans, 2002; McFadden et al, 2012; Booth, 2016) will be searched from October 2015 (DVA) and July 2014 (CM): ASSIA, CINAHL, GoogleScholar (first 100 hits), PsycInfo and SSCI.

This review is building on Howarth et al. (2016) and Macdonald et al. (2016) so relevant studies from these reviews (and related work such as Arai et al. (2019)) will be included. In addition, expert recommendations of relevant qualitative studies or reviews and any qualitative studies identified from the reviews of systematic reviews will be included.

For peer review only - http://bmjopen.bmj.com/site/about/guidelines.xhtml

The search strategy will use the same terms for DVA/CM as the review of systematic reviews, plus additional search terms to identify qualitative research. These will be adapted as required for the different databases with guidance from an expert librarian.
These reviews will be carried out separately for DVA and CM. The DVA search will be run first and any CM studies that do not mention DVA will be excluded (and vice versa) but put aside for inclusion in the relevant review. This review will not adhere to set definitions of DVA/CM, thus any study deemed by the research team to address the phenomena of interest will be included and justified in the discussion of findings.

Screening
Screening of abstracts from the searches and articles included in the full text stage will be guided by questions asked in the IMPROVE study (Howarth et al., 2016):

1) Is this qualitative research?
2) Is there sufficient verbatim text? (i.e. more than 2 lines)
3) Does the paper discuss perspectives of experienced or anticipated benefits or harms of interventions, and/or desired outcomes in general related to DVA/CM.

All articles identified by searches will be downloaded to CADIMA (Kohl et al., 2018) and de-duplicated. Screening criteria will be tested by two reviewers on 10% titles/abstracts and interrater reliability assessed. Titles/abstracts will be screened by one reviewer for inclusion in full-text review. A second reviewer will independently review 10% of title/abstracts. If there is a high level of disagreement, the second reviewer will continue reviewing titles/abstracts until agreement is reached. Full-text systematic reviews will be screened for inclusion and a second reviewer will independently review 10% of these as above. Key details (e.g. bibliographic information, study design, setting, participants etc.) about each full-text inclusion will be recorded in Access.

Strategy for data synthesis
Thematic frameworks will be developed from the IMPROVE study (Howarth et al., 2016) for DVA and the parallel CM study (MacDonald et al., 2016), and input into NVivo 11 (QSR International). The frameworks will focus on barriers and harms of interventions according to parents, children and stakeholders, based on the research questions. These will be used as the basis for a framework analysis (Ritchie & Lewis, 2003) of the studies from the review (Howarth et al., 2016; Arai et al., 2019; Macdonald et al., 2016). As per Howarth et al. (2016), participant quotations and author-identified themes will be extracted rather than line by line coding. Findings will be grouped by whose view was reported and extracts from the texts will be categorised according to this framework with the aim will be to meta-aggregate the studies' findings. Further categories will be developed where there are discrepancies or gaps in the initial framework.

The analysis and interpretation of the findings will occur at the synthesis stage in order to provide an overview of the findings, informed by the principles of meta-synthesis (c.f. Noblit & Hare, 1988), although using a lighter touch given time constraints. Two researchers will work together throughout this process to ensure consistency of categorisation and analysis. Quality will not be assessed because the aim of the review is solely to identify candidate outcomes. The ENTREQ statement (Tong et al., 2012) will be followed for the write-up.

For peer review only - http://bmjopen.bmj.com/site/about/guidelines.xhtml

C – Rapid review of grey literature

Review questions:

1) How are DVA and CM defined in relevant UK service policy contexts?
2) How are outcomes defined: (i) in UK service-based evaluations of interventions? (ii) in relevant policy or commissioning frameworks?

This review will be carried out as a single process given the likelihood of crossover literature. Findings will be recorded as DVA or CM or both.

Literature inclusion:

- Any national or regional policy or practice document that reports on DVA/CM-relevant services or outcomes (e.g. measurement/theory).
- Participation in the service is determined by experience, perpetration or identified as at risk of DVA/CM. (Identification of risk is by practitioners or participants thus we do not have a definition).
- Published since 2016 to build on Howarth et al. (2016) and Macdonald et al. (2016).
- England-based only. English language only.

Exclusion: Publication in academic journals, book chapters, conference papers, theses and dissertations.

Population inclusion: children or families with children at risk of experiencing, or experiencing DVA/CM. This includes unborn children, children (aged 0 to 18 years), designated as victim or witness. For DVA any adult family members who have a caring or parenting role (Early Intervention Foundation, 2014), whether designated as perpetrator, victim, witness or household member. For CM any adult family members who have a caring role, whether designated as perpetrator, witness or household member.

Service inclusion: Any services where:

- Experience of or increased risk of experiencing DVA/CM is a criterion for being offered the service/intervention.
- Services/interventions may be delivered to any family member(s) as an individual or in a group. Any duration of service/intervention will be included. Any setting will be considered.

OR

- Any evaluative work or outcomes framework where at least one child or family-level outcome is evaluated/discussed. Family-level outcomes do not need to be explicitly labelled as 'family' level, we will make a judgement. However, they include any outcome that affects the family/household unit. For example, worklessness in study where at least some participants are reported to be parents would be included.

Exclusion: universal services/interventions that do not specifically target children and families at risk of DVA/CM; targeted services/interventions that do not measure any child or family level outcomes e.g. perpetrator programmes that focus solely on attitudinal change; DVA (only) services/interventions focused solely on elder abuse, sibling abuse or child perpetration of domestic violence, where participants have not been identified as exposed to DVA (i.e. perpetration of abuse by a child could feasibly be an outcome associated with exposure).

For peer review only - http://bmjopen.bmj.com/site/about/guidelines.xhtml

For peer review only

Outcome inclusion: Any family or child-level outcome measured or evaluated or discussed in any way. Intermediate outcomes that could feasibly represent preconditions needed to reach distal/final outcomes (including those relating to the process of service delivery) will be included, along with final/distal outcomes.

Searches

The following databases and websites will be searched:

Grey databases: NICE Evidence Search and Open Grey

Organisation websites including but not limited to:

DVA: Women's Aid, Refuge, Respect, Safe Lives, Voices, AVA, Standing Together, Imkaan, The Stefanou Foundation, Women's Trust, Hestia, DVIP, Nia, The Haven, ManKind Initiative, Everyman Project, NCDV, Galop, LAWA, IDAS, Advance, Your Sanctuary, Advocacy After Fatal Domestic Abuse (AAFDA); Aurora New Dawn; My Sister's Place

CM: Centre of expertise on child sexual abuse, FDAC, SCIE, The Survivors' Trust

General websites: Victim Support, Barnardos, NSPCC, Early Intervention Foundation, NatCen, RCGP, RCN, RCM, NICE, BPS, IHV, WHO, UNICEF, Working together, gov.uk (incls e.g. DA bill, 'Working together'), Public Health for any UK nation, Office of the children's commissioner for any UK nation, Big Lottery, Comic Relief, The Childhood Trust, UK College of Policing, Research in Practice, 'What Works', Joseph Rowntree Foundation, What Works for Children's Social Care.

Websites will be searched manually for relevant documents. It is anticipated there will be an element of snowball searching as relevant organisations will have links to further organisations. Searches will be run simultaneously and then relevant reports assigned to DVA/CM or both. All websites searched will be recorded in Excel/Access along with relevant details about any reports captured. The expert reference group will be consulted about relevant websites to search or reports to include at multiple timepoints.

Data extraction and synthesis

As a range of types of data are anticipated, both the systematic review and the qualitative review protocols will be adapted as necessary to capture and record relevant information. It is likely that there will be non-standardised evaluation measures and interview quotations. Report identification from websites/databases will be carried out by a single researcher and the process transparently recorded. All details regarding evaluation studies and relevant outcomes will be recorded, and where necessary synthesised when the data is qualitative. Access/Excel/NVivo will be used as required to record all steps and ensure a transparent process. A second researcher will cross-check a subset of the reports and the data extracted to ensure consistency and focus on the review questions.

For peer review only - http://bmjopen.bmj.com/site/about/guidelines.xhtml

Bibliography

Arai, L., Heawood, A., Feder, G., Howarth, E., MacMillan, H., Moore, T. H. M., Stanley, N., & Gregory, A. (2019). Hope, Agency, and the Lived Experience of Violence: A Qualitative Systematic Review of Children's Perspectives on Domestic Violence and Abuse. *Trauma, Violence, & Abuse*, 152483801984958. https://doi.org/10.1177/1524838019849582

CRD. (1995). *Database of Abstracts of Reviews of Effects (DARE): Quality-assessed Reviews*. Centre for Reviews and Dissemination (UK).

Early Intervention Foundation. (2014). *Early Intervention in Domestic Violence and Abuse*.

Howarth, E., Moore, T. H., Welton, N. J., Lewis, N., Stanley, N., MacMillan, H., Shaw, A., Hester, M., Bryden, P., & Feder, G. (2016). IMPRoving Outcomes for children exposed to domestic ViolencE (IMPROVE): An evidence synthesis. *Public Health Research*, *4*(10), 1–342. https://doi.org/10.3310/phr04100

Kohl, C., McIntosh, E. J., Unger, S., Haddaway, N. R., Kecke, S., Schiemann, J., & Wilhelm, R. (2018). Online tools supporting the conduct and reporting of systematic reviews and systematic maps: A case study on CADIMA and review of existing tools. *Environmental Evidence*, *7*(1), 8. https://doi.org/10.1186/s13750-018-0115-5

Macdonald, G., Livingstone, N., Hanratty, J., McCartan, C., Cotmore, R., Cary, M., Glaser, D., Byford, S., Welton, N. J., Bosqui, T., Bowes, L., Audrey, S., Mezey, G., Fisher, H. L., Riches, W., & Churchill, R. (2016). The effectiveness, acceptability and cost-effectiveness of psychosocial interventions for maltreated children and adolescents: An evidence synthesis. *Health Technology Assessment*, *20*(69), 1–508. https://doi.org/10.3310/hta20690

Maclean, M. J., Sims, S., O'Donnell, M., & Gilbert, R. (2016). Out-of-Home Care versus In-home Care for Children Who Have Been Maltreated: A Systematic Review of Health and Wellbeing Outcomes: Systematic Review of Outcomes of Out-of-Home Care. *Child Abuse Review*, *25*(4), 251–272. https://doi.org/10.1002/car.2437

Study design search filters | BMJ Best Practice. (n.d.). Retrieved 16 April 2019, from https://bestpractice.bmj.com/info/us/toolkit/learn-ebm/study-design-search-filters/

Tong, A., Sainsbury, P., & Craig, J. (2007). Consolidated criteria for reporting qualitative research (COREQ): A 32-item checklist for interviews and focus groups. *International Journal for Quality in Health Care*, *19*(6), 349–357. https://doi.org/10.1093/intqhc/mzm042

For peer review only

BMJ Open

Protocol for developing Core Outcome Sets for evaluation of psycho-social interventions for children and families with experience or at risk of child maltreatment or domestic abuse

Journal:	*BMJ Open*
Manuscript ID	bmjopen-2020-044431.R2
Article Type:	Protocol
Date Submitted by the Author:	24-Jun-2021

Complete List of Authors:	Howarth, Emma; University of East London; University College London, Institute of Child Health<br>Powell, Claire; University College London, Institute of Child Health<br>Woodman, Jenny; University College London, Institute of Education<br>Walker, Erin; University College London, UCL Partners<br>Chesters, Heather; University College London, Institute of Child Health<br>Szilassy, Eszter; University of Bristol Medical School, Centre for Academic Primary Care, Population Health Sciences, Bristol Medical School<br>Gilbert, Ruth; University College London Institute of Child Health, Centre for Paediatric Epidemiology and Biostatistics<br>Feder, Gene; University of Bristol Medical School, Community based medicine

**Primary Subject Heading**:	Public health
Secondary Subject Heading:	Research methods
Keywords:	Child protection < PAEDIATRICS, Community child health < PAEDIATRICS, PUBLIC HEALTH, SOCIAL MEDICINE

SCHOLARONE™
Manuscripts

BMJ

I, the Submitting Author has the right to grant and does grant on behalf of all authors of the Work (as defined in the below author licence), an exclusive licence and/or a non-exclusive licence for contributions from authors who are: i) UK Crown employees; ii) where BMJ has agreed a CC-BY licence shall apply, and/or iii) in accordance with the terms applicable for US Federal Government officers or employees acting as part of their official duties; on a worldwide, perpetual, irrevocable, royalty-free basis to BMJ Publishing Group Ltd ("BMJ") its licensees and where the relevant Journal is co-owned by BMJ to the co-owners of the Journal, to publish the Work in this journal and any other BMJ products and to exploit all rights, as set out in our licence.

The Submitting Author accepts and understands that any supply made under these terms is made by BMJ to the Submitting Author unless you are acting as an employee on behalf of your employer or a postgraduate student of an affiliated institution which is paying any applicable article publishing charge ("APC") for Open Access articles. Where the Submitting Author wishes to make the Work available on an Open Access basis (and intends to pay the relevant APC), the terms of reuse of such Open Access shall be governed by a Creative Commons licence – details of these licences and which Creative Commons licence will apply to this Work are set out in our licence referred to above.

Other than as permitted in any relevant BMJ Author's Self Archiving Policies, I confirm this Work has not been accepted for publication elsewhere, is not being considered for publication elsewhere and does not duplicate material already published. I confirm all authors consent to publication of this Work and authorise the granting of this licence.

For peer review only

TITLE	Protocol for developing Core Outcome Sets for evaluation of psycho-social interventions for children and families with experience or at risk of child maltreatment or domestic abuse
Emma Howarth	School of Psychology, UEL<br>University Way, London, UK E16 2RD
Claire Powell<br>(Corresponding author)	Institute of Child Health, UCL<br>30 Guilford St, London, UK WC1N 1EH<br>Email: c.powell@ucl.ac.uk
Jenny Woodman	Institute of Education, UCL<br>27/8 Woburn Square, London, UK WC1H 0AA
Erin Walker	UCL Partners<br>170 Tottenham Court Road, London, UK W1T 7HA
Heather Chesters	Institute of Child Health, UCL<br>30 Guilford St, London, UK WC1N 1EH<br>United Kingdom
Eszter Szilassy	School of Social and Community Medicine, University of Bristol<br>39 Whatley Road, Bristol BS8 2PS<br>United Kingdom
Ruth Gilbert	Institute of Child Health, UCL<br>30 Guilford St, London, UK WC1N 1EH<br>United Kingdom
Gene Feder	School of Social and Community Medicine, University of Bristol<br>39 Whatley Road, Bristol BS8 2PS<br>United Kingdom
**KEY WORDS**	Core outcome set; intervention; child maltreatment; domestic abuse; evaluation
**WORD COUNT**	5119

For peer review only

For peer review only - http://bmjopen.bmj.com/site/about/guidelines.xhtml

ABSTRACT

Introduction

Recognition that child maltreatment (CM) and domestic violence and abuse (DVA) are common and have serious and long-term adverse health consequences, has resulted in policies and programmes to ensure that services respond to and safeguard children and their families. However, high quality evidence about how services can effectively intervene is scant. The value of the current evidence base is limited partly because of the variety of outcomes and measures used in evaluative studies. One way of addressing this limitation is to develop a Core Outcome Set (COS) which is measured and reported as a minimum standard in the context of trials and other types of evaluative research. The study described in this protocol aims to develop two discrete core outcome sets for use in future evaluation of psycho-social interventions aimed at improving outcomes for children and families at risk or with experience of i) CM or ii) DVA.

Methods and analysis

A two-phase mixed methods design: 1) rapid reviews of evidence, stakeholder workshops and semi-structured interviews with adult survivors of CM/DVA and parents of children who have experienced CM/DVA; 2) a three panel adapted E-Delphi study and consensus meeting. This study protocol adheres to reporting guidance for COS protocols and has been registered on the COMET database.

Ethics and dissemination

We will disseminate our findings through peer reviewed and open access publications, the COMET website, and presentations at international conferences. We will engage with research networks, journal editors and funding agencies to promote awareness of the CM- and DVA-COS. We will work with advisory and survivor and public involvement groups to co-produce a range of survivor, policy and practice facing outputs.

Approval for this study has been granted by the Research Ethics Committee at University College London.

STRENGTHS AND LIMITATIONS OF THIS STUDY

For peer review only - http://bmjopen.bmj.com/site/about/guidelines.xhtml

- To our knowledge this is the first attempt to develop core outcome sets to address family violence and abuse.
- The study draws on diverse evidence sources and includes people with lived experience, practitioners and policy makers, as well as researchers.
- This study provides the opportunity to consider the overlap in outcomes sought across two different but related exposures.
- This study is limited by the lack of direct involvement of children and young people.
- It is beyond the means of the study to involve survivors and service providers from low- and middle-income countries (LMICs), although we will include research from LMICs in the evidence reviews and actively recruit researchers from or researching LMIC settings.

For peer review only

For peer review only - http://bmjopen.bmj.com/site/about/guidelines.xhtml

INTRODUCTION

Widespread recognition that child maltreatment (CM) and domestic violence and abuse (DVA) are common and have serious and long-term adverse health consequences, [1,2] has resulted in policies and programmes to ensure that services respond to and safeguard children (and their families) at high risk of, or with experience of CM and or/DVA.[3–6] However, high quality evidence about how services can *effectively* intervene is scant. [7–9]

The value of the current evidence base is limited partly because of the variety of outcomes and measures used in evaluative studies. [7,8]. This hampers the ability to aggregate evidence pertaining to one particular type of intervention, so as to build a comprehensive picture of its effectiveness when delivered to different populations or in different contexts. Similarly it is challenging to make comparisons between different types of interventions, which purport to address the same problem within the same group of individuals. [10,11]

More fundamentally, outcomes measured in CM and DVA intervention studies are often a poor or partial reflection of the concepts of success held by those that use, deliver and pay for interventions. [7,8,12] The ultimate goal of intervention studies is to identify interventions that can benefit individuals, families and communities in the future. Therefore, it is crucial that they measure outcomes reflecting the priorities and expectations of these groups so the evidence they generate is relevant to consumers. Outcomes also need to resonate with the priorities of policymakers and service providers, else effective interventions may be overlooked by those responsible for funding and/or delivery decisions, and never commissioned or implemented.[13,14]

Together, these issues mean it is difficult to extract the information needed to inform real world decisions about which CM/DVA interventions to commission and scale, and which to stop funding.

One way of addressing the limitations set out above is to develop a Core Outcome Set (COS), a standardised set of outcomes that researchers, providers, service users, and commissioners consider critical or important outcomes in the management of a condition or in this case, a complex public health challenge. [11,15] The COS is then measured and reported, as a minimum standard in the context of trials or other types of research and evaluation, [15] and sometimes practice-based monitoring. [16] The aim is to enhance the methodological standard and utility of research in the field, by increasing consistency and reducing reporting bias (where many outcomes are measured and only favourable effects reported), and ensuring the views of important constituencies influence the selection of outcomes to be included in the COS. [10]

For peer review only - http://bmjopen.bmj.com/site/about/guidelines.xhtml

The idea of the COS as a mechanism for improving evidence quality has gathered momentum over the past decade since the establishment of the COMET (Core Outcome Measures for Effectiveness Trials) initiative in 2010 (www.comet-initiative.org). [15] Whilst the number of core outcome sets being developed has increased steadily since, [16,17] it is clear that in the main, studies have focused on COS development for specific health conditions, pharmacological or surgical interventions and/or discrete interventions delivered in health care settings. [16,17] In contrast there has been relatively less focus on the development of COSs in relation to public health problems that require complex multi-sectoral responses, often delivered to whole families or multiple members of the same family.

Current study

The study sets out to develop two discrete core outcome sets for use in future evaluation of psycho-social interventions, which aim to improve outcomes for children and families at risk of or with experience of CM or DVA. We use the term 'at risk' so as not to limit the scope of this work to those interventions delivered to families following substantiated experience of CM or DVA or where children and families define their experiences as such; but to include interventions offered to families where it is suspected that an exposure may have taken place, or where children's experiences are thought to be on a trajectory towards this.

Children's experiences of CM and DVA frequently overlap [18] and experience of DVA is often conceived of as a type of maltreatment in its own right, or a feature of emotional maltreatment. [19,20] Nevertheless, the conceptualisation and response to these two types of trauma can be different, despite similar consequences. For example, there is variation as to whether exposure to DVA is considered as a form of child maltreatment. Where it is, evidence suggests there may be different levels of state intervention where the primary concern is exposure to DVA versus experience of CM. [19,20]. This provides the rationale for developing separate outcome sets, however we will explore where the derived outcome sets overlap with a view to identifying outcomes that can be measured in family contexts where *both* CM and DVA occur. This is a move away from a focus on single problem areas towards recognition of the constellation of risks often experienced by children and their families.

METHODS AND ANALYSIS

This study protocol adheres to reporting guidance for core outcome set protocols [21] and has been registered on the COMET database.

Scope of outcome sets

For peer review only - http://bmjopen.bmj.com/site/about/guidelines.xhtml

The CM-COS and the DVA-COS will be developed to support evaluation of the impact of targeted child and/or family focussed psychosocial interventions or services, in the context of both research (randomised and non-randomised studies) and practice (service evaluations and monitoring). The target population for interventions is children aged less than 19 years of age with experience of (current or previous) DVA or CM. Given that many interventions aiming to improve child outcomes do so via support delivered to parents or multiple family members (rather than directly to the child), [7,8,22] the target group also includes parents or families of children experiencing CM or DVA. We use a definition of Psychosocial Interventions set out by the Institute of Medicine. [23] Interventions within the scope of this study include psychotherapies (e.g. cognitive-behavioural therapy), community-based treatments, family/systemic therapy, vocational rehabilitation, peer support services, integrated care interventions, and out-of-home care (i.e. foster care or adoption). Interventions may be delivered in one or more contexts (e.g. clinic, school, community). Interventions may be individual, dyad or group based, or a combination, and delivered to children with or without their parents, to parents alone, to family groups, or some combination. To be in scope an intervention must implicitly or explicitly aim to improve child outcomes by one or more of the following mechanisms: i) reducing the risk of CM/DVA occurring/reoccurring in the family; ii) improving parental (non-harming and/or harming) functioning as an indirect route to improving child outcomes; iii) limiting or preventing poor mental health, reduced wellbeing or function in children following exposure; iv) promoting children's recovery following experience of CM or DVA – here we relate to the recovery model definition which emphasises perceptions of resilience, self-identity, a sense of empowerment, hope and optimism. (e.g. [24]) Universal and targeted structural interventions are not in scope.

Study design

The study is being undertaken in two stages (see Figure 1). The first stage is underway and seeks to identify candidate outcome areas, domains and indicators. Multiple methods are being used to identify items for the candidate list including rapid evidence reviews, consultation with key stakeholders and qualitative interviews. Data will be synthesised to produce a taxonomy of outcomes, from which the two candidate lists of indicators (structured by area and domain) will be produced.

The second stage, due to begin in April 2021, will incorporate an adapted two round E-Delphi study and consensus meeting, with the aim of building agreement between different stakeholder groups regarding important outcomes. The E-Delphi technique is an iterative, multistage, online process designed to seek opinion from and develop consensus among a defined group of individuals (panel).

For peer review only - http://bmjopen.bmj.com/site/about/guidelines.xhtml

The method is frequently used when evidence in an area is known to be limited or contradictory and is widely used in health and social care research. Key features include (1) an anonymous survey process, whereby a panel (or multiple panels) of experts (by profession and/or experience) use a questionnaire to rate a series of statements over a number of rounds; (2) the provision of structured feedback to panel members between rounds with the ability to adjust ratings in light of knowledge about the group opinion and (3) anonymity for panel members during the process.[25]These features can facilitate the convergence of opinion across rounds, helping to build consensus while at the same time highlighting areas of continuing disagreement. This method has been used extensively in the context of core outcomes research [16,26,27].

We will recruit three panels for participation in the E-Delphi study to ensure that each stakeholder group is equally represented in the final consensus. [28] In a further effort to ensure the views of those with lived experience remain a central focus during this exercise, the E-Delphi method will be adapted so that in addition to feedback about their individual and own panel scores for each item, professional and researcher panels will also receive feedback about the scores of the lived experience panel. This adaptation is informed by evidence that feedback of patient scores to clinicians results in an expanded set of consensus items that better reflect the priorities of patients. [29] Additional feedback will not be given to the lived experience panel, so as to minimise the possibility of perceived power differentials influencing this panel's ratings. [28] A final face to face consensus meeting will be used to review and verify findings from the E-Delphi study, clarify any remaining uncertainty, and ratify the final core outcome set.

[Figure 1 about here]

Study Oversight

A steering committee including practitioners, policy makers and researchers representing CM and DVA fields has been formed and will meet formally twice a year.

Patient and Public Involvement

Three public advisory groups are also overseeing and consulting on the study. One group is comprised of individuals with lived experience of DVA and one of care experienced young people. These groups have been formed in partnership with relevant survivor led organisations. A third group is comprised of young people affiliated to the National Children's Bureau who are consulting more broadly on the work of the Children and Families Policy Research Unit. Partner organisations are funded to organise three meetings per year and to provide appropriate remuneration to participants. Additional funds will be paid to cover scheduled review activities organised with

For peer review only - http://bmjopen.bmj.com/site/about/guidelines.xhtml

partner agencies via email. Members of advisory groups will be involved in all aspects of the study including the development of the outcomes taxonomy, development of the list of candidate indicators, preparation of materials for the E-delphi and dissemination of results.

Participants

Workshops (Phase 1*)*: We will invite 30-40 individuals to attend each workshop, the aim of which will be to discuss definitions of CM/DVA and outcomes perceived to be important for survivors. Relevant researchers (mainly UK) and professionals from each field (e.g. support workers, primary and secondary health practitioners, education staff, local authority commissioners, local and national policy makers) will be identified from the research team's networks, authorship of key publications, and internet searches.

People with lived experience of CM/DVA will be approached via gatekeeper organisations and existing survivor/researcher networks known to the research team. Concerted effort will be made to invite individuals representing groups known to be marginalised from services or research on DVA/CM, or who receive inadequate service responses owing to discrimination or lack of service differentiation (i.e. assuming all groups require the same response). [30–33]

Semi-structured interviews (Phase 1*)*: We will recruit a sample of approximately 5 adults who identify as survivors of CM or exposure to DVA during childhood, and 5 parents of children currently aged 0-18 with lived experience of DVA/CM. In the first instance we will seek to recruit participants via gatekeeper organisations (see procedure below), although if recruitment is insufficient, we will seek approval for direct recruitment via social and print media. To take part in interviews, participants will be required to self-identify as having experienced CM/DVA, or as having a child who has experienced CM/DVA.

Adapted international E-Delphi study (Phase 2): Three separate panels will be recruited to take part in the consensus study comprising: i) individuals with lived experience (parents of children with experience of CM/DVA and adults experiencing abuse in childhood); ii) frontline and strategic professionals involved in the delivery and commissioning of CM/DVA services and related policy; iii) researchers. The first two panels will include members from the UK, with the researcher panel including international researchers from high, middle- and low-income countries. We will aim to recruit 30 individuals to each panel.

Survivors and professionals taking part in the workshops and semi-structured interviews described above (and who give consent for further contact) will be approached for participation in the lived experience and professional panels respectively. If needed, additional participants will be recruited

For peer review only - http://bmjopen.bmj.com/site/about/guidelines.xhtml

through key organisations working with either CM or DVA survivors and snowball sampling. Key researchers, with at least one peer-reviewed publication from either the CM/DVA field, will be identified through the rapid reviews, researcher networks, participation in workshops, and via the expert panel. For all panels, participants must be able to read and understand English in order to participate.

Consensus workshop following E-Delphi study (Phase 2): A face-to-face consensus meeting, with a purposively sampled panel (n=30) representing all key stakeholder groups, will be recruited from participants taking part in earlier phases of the study. Individuals outside of the study will be approached as needed to ensure balanced representation and inclusion of individuals of strategic importance to take up and implementation of study findings. Appropriate amendments to ethical approvals will be sought to accommodate this.

It is important to note that although the focus of this work is on child and family targeted interventions, this study does not directly involve children and young people aged <18 years with experience of child maltreatment and/or domestic violence and abuse. We initially explored this possibility with third sector organisations and professionals and clinicians comprising our expert advisory group. However, it was concluded that the nature of this research was not sufficient to justify the potential harm and safeguarding issues that may have been raised by approaching children and young people with recent experience of violence and abuse, particularly as they may not be engaged with supportive services. Instead the voices of children and young people have been included indirectly via i) inclusion of outcomes extracted from qualitative studies reporting children and young people’s experiences, ii) recruitment of adult survivors of CM and childhood exposure to DVA as well as parents of children with recent experience, iii) and via consultation with care experienced young people who are advising on the conduct of the study, including review of outcomes identified in the first phase of this work. Nevertheless, the lack of children and young people’s direct participation is a limitation to this work, that will be transparently addressed at all stages of reporting.

Procedure

Phase 1

Rapid reviews

We will conduct a series of rapid reviews using systematic methods (see supplementary appendices for protocols and review questions). We will review experimental and quasi-experimental

For peer review only

For peer review only - http://bmjopen.bmj.com/site/about/guidelines.xhtml

intervention studies (international), qualitative studies containing primary accounts of experience of relevant interventions or outcomes that are sought by families and children experiencing CM/DVA (international), and grey (UK) literature reporting descriptions of interventions, service evaluations or consultation regarding appropriate outcomes across the DVA and CM fields.

We will search a range of relevant databases and websites under the guidance of an expert librarian. Following rapid review techniques [34,35] we will search since 2014 for intervention studies (covering the time elapsed since previous key reviews, [8,36]) and 2014/5 for the qualitative studies to build on recent qualitative reviews. [37] The grey literature review will primarily focus on searches of relevant UK organisation websites and will include any service or intervention evaluation or any consultation or review, to identify relevant candidate outcomes or outcome tools for use in the context of service delivery or evaluation.

A second reviewer will screen and extract data from a minimum of 5% of titles/abstracts and articles to ensure consistency. Inter-rater reliability kappa scores will be calculated, and disagreements resolved through discussion (or a third reviewer if necessary) throughout the process. Relevant outcome indicators will be extracted, as well as their measurement instruments where possible. There will be no appraisal of study quality and outcomes will be extracted from all identified papers.

Stakeholder workshops

We will hold two invite-only workshops (one focussed on CM and one focussed on DVA) to gather stakeholder views. The purpose of these events will be to i) explore definitional issues, specifically how each phenomenon is defined by particular groups and the function that this definition plays in practice (in terms of enabling access to services/interventions and measuring change), and ii) to explore outcomes perceived to be important indicators of benefit or harm for children and families experiencing CM/DVA.

Participants will be seated on tables of 6-8. Each table will include at least two individuals with lived experience and one facilitator. Guided by facilitators, participants will be asked to generate ideas relating to desirable (or undesirable) outcomes, unconstrained by what they believe to be measurable or achieved via currently available interventions. This will be an attempt to ensure output is not merely reflective of current practice or discourse. Designated scribes will take notes throughout the day, which will be collated and analysed thematically. [38] Participants in the workshops will be asked for permission to contact them at a later date for the purpose of inviting them to participate in the international E-Delphi study.

Interviews with individuals with lived experience of DVA/CM as a child or as parent of a child

For peer review only

For peer review only - http://bmjopen.bmj.com/site/about/guidelines.xhtml

Participants will be identified via key gatekeeper organisations (where work with survivors of CM/DVA is core business) contacted for the purpose of workshop participation (see above). Participants will be approached directly by a professional from the gatekeeper organisation or they will receive an open invitation circulated through the organisation's survivor network. Where participants are approached by professionals, they will be given brief information about the study and asked for permission to pass contact details to the research team. Individuals responding to an open invitation will be asked to contact a member of the research team directly. They will be assured of the anonymity of their involvement.

Basic socio-demographic information and minimal information about experiences of CM or DVA will be collected via questionnaire prior to the interview and will be used for sample description. Participants will have the opportunity to take part in the interview face-to-face, by video call or by phone, according to their personal preferences and public health guidance on social distancing. For those participants who wish to take part but are unable to speak directly to interviewers, they will be able to answer the interview questions by email. [39] Interview schedules will be used to guide interviews, which will be recorded and transcribed verbatim and analysed thematically. [38]

Outcome generation

A list of candidate outcome areas (e.g. health and wellbeing), domains (e.g. mental health) and specific indicators (e.g. withdrawal from friends and activities) will be generated iteratively by the research team, drawing on all information sources described above. An unedited candidate list of outcome indicators generated from stakeholder workshops will be used as a starting point. Identification of duplicate and overlapping outcome indicators from the list will be undertaken in parallel by two team members (CP, EH). Similar items will be dropped or combined to produce a reduced inventory. Disagreements between team members will be resolved through discussion. All suggestions to drop or combine items will be reviewed by two further research team members (RG, GF) and survivor involvement groups. Similar indicators (i.e., outcomes that could be compared across studies or combined in a meta-analysis [21]) will be grouped into outcome domains by two team members, and reviewed by two further members of the research team and survivor involvement groups. Simultaneously, a taxonomy to organise domains into broader outcome areas will be developed. Here we will draw on existing practical and theoretical frameworks to categorise health outcomes, [40] as well as the aetiology and impacts of DVA and CM. [41–44] This overarching framework to describe the hierarchical structure of outcomes identified in workshops will be reviewed and refined by all members of the research team, the expert advisory group, and survivor involvement groups.

For peer review only - http://bmjopen.bmj.com/site/about/guidelines.xhtml

A candidate list of outcome indicators from the rapid reviews will be generated and de-duplicated (CP, EH). Four research team members and at least two survivor representatives will, in parallel, attempt to categorise indicators using the developed taxonomy. Categorisations will be compared, disagreements discussed, and consensus reached through discussion. New domains or areas will be added where necessary. Unique indicators (not already included) will be identified from the candidate list generated from the reviews and added to the taxonomy. This iterative process will be repeated with data yielded from interviews.

The final taxonomy and labelling of terms will be reviewed by the advisory group, and all three public involvement groups. Particular attention will be given to the language used to describe outcome areas, domains, and specific indicators to ensure they are understandable, meaningful and acceptable to all stakeholder groups. Further refinement (including addition of areas, domains or indicators) will be undertaken following this review. The final step in the process will be to examine outcomes against a priori criteria designed to ensure the final COS has maximum utility. These include: i) the extent to which the outcome indicator relates to children's feelings, function or survival, or the process of delivering services to survivors ii) whether the outcome is 'changeable' iii) and whether the outcome indicator could feasibly change as a result of a psychosocial intervention – here we will draw on literature elucidating mechanisms through which exposure to violence and abuse may be communicated to child outcomes. (e.g. [45]) Four members of the research team, at least two members of the expert advisory group, and four members of the survivor involvement groups (with equal representation of CM and DVA experience) will independently assess outcome indicators against the criteria listed above. Any indicators identified as not meeting all criteria by one or more reviewers will be discussed and a majority decision taken to exclude or include it in the candidate list. Excluded outcomes will be reported in the final paper, along with reasons for exclusion. Where needed a glossary of terms and explanatory text will be developed to aid clarity for participants in the E-Delphi study.

Phase 2

Adapted international -Delphi study

A sequential two-round, three-panel E-Delphi study will be conducted.

Round 1*:* A questionnaire for use in the E- Delphi study will be developed using the taxonomy described above. Areas and domains will serve as headings and sub-headings by which to organise the survey, so as to encourage completion and to allow us to explore the relative importance of indicators within the same domain. The questionnaire will be reviewed by advisory and involvement

For peer review only - http://bmjopen.bmj.com/site/about/guidelines.xhtml

groups and refined in line with feedback. Ethical approval will be sought as an amendment to that granted for phase one of the study.

Participants will be contacted by email to remind them about the COS study and their attendance at a previous workshop (if appropriate) and to invite them to participate in the E-Delphi study. A second email containing the information sheet and link to an online questionnaire will be sent one-two days after the initial contact. Participants will be required to indicate that they have read the information sheet and agree to take part, before proceeding to the questionnaire. The questionnaire will be administered via Qualtrics (https://www.qualtrics.com/uk/)hosted by the University College London.

Participants will be presented with a list of outcome indicators organised by area and outcome domain. They will be asked to rate each outcome presented, on a nice-point scale of importance (1=not at all important, 9=extremely important). Participants will also be given the opportunity to add any additional outcomes that are missing from each domain using a free text comments box. During this round we will also collect demographic data including ethnicity, age, gender, profession and country of professional operation. The questionnaire will remain open for 14 days and reminder emails will be sent out seven and two working days before closure.

Item level descriptive statistics will be generated for each panel and item including: number of respondents, minimum and maximum values, measures of central tendency and dispersion. Criteria for item inclusion in round two will be an item is rated seven-nine(on a 9-point Likert scale) by 50% or more participants in at least one panel and one-three by no more than 15% of participants in any stakeholder group. [46]This low threshold for inclusion enables us to reduce response burden in round two by dropping unimportant items given higher number of items are associated with significantly lower response rates in COS Delphi surveys, [47] while also reducing the likelihood of dropping outcomes that may have been rated more highly in subsequent rounds had participants been given feedback on them. New items will be included if two or more panellists suggest inclusion, and the research team deem it unique to existing content. [15] Panellists completing Round one will be invited to participate in Round two if they rated ≥50% of survey items. Non-completers will not be contacted for participation in round two. We will assess attrition rates for each panel and by demographic profiles.

Round two*:* An amendment to the existing approval will be sought for use of the shorter round two questionnaire. The same items will be included in questionnaires issued to each panel. Each panel member will receive a personalised questionnaire reporting panel averages and their own rating for each item. As noted above, professional and researcher panels will also receive feedback about the

For peer review only

ratings of the survivor panel. Panellists will be asked to re-rate each of the included items, and rate for the first time, any new outcomes put forward in round one. All new outcomes suggested in round one (irrespective of the panel from which they derived) will be presented to each of the three panels.

As before, participants will receive two reminders to complete the questionnaire, over the course of 14 days. Following completion of the study, descriptive statistics will be computed. Items will be deemed important to a particular panel if they are rated seven -nine by ≥70% of respondents and one - three ≤15% by the panel. Conversely, items will be classified as unimportant to a group if ≥70% of respondents rate it as one-three and ≤15% rate it as seven-nine. Any items not classified as important or unimportant will be deemed not to have reached consensus. Items will be considered 'core' and recommended for inclusion in the COS if they are rated as important by all three panels. We will assess the impact of attrition on consensus by comparing (within panels) the mean total item scores for those completing round 1 only and those completing both rounds; we will also compare the average scores for completers vs non completers by each item (within panel).[15]

Consensus meeting

A face-to-face consensus meeting, with a purposively sampled panel (n=30) representing all key stakeholder groups, will be held to discuss, vote and agree on the final CM- and DVA COS. The format of the meeting will follow the process set out by the James Lind Alliance (JLA) final priority setting workshops http://www.jla.nihr.ac.uk/jla-guidebook/chapter-8/workshop-process-on-the-day.htm This method is pertinent given that JLA priority setting meetings involve multiple stakeholders, discussion of interim results derived from the ranking of evidence uncertainties, and production of a 'top ten'.

Whilst there is no recommended maximum number of outcomes that should be included in a COS, for it to be pragmatic we aim to arrive at a maximum of 10 outcomes. [48,49] The JLA priority setting method involves a structured process including small group and whole group discussion, ranking and reranking. The method will be adapted to include a preliminary step, where participants review those outcomes identified as important to the lived experience panel, but which didn't reach consensus across all groups. Participants will be asked to identify any outcomes that should be discussed in the workshop, alongside outcomes meeting the consensus definition. This initial step is an attempt to ensure appropriate weight is given to the voice of those with lived experience of DVA/CM. During discussion, workshop participants will be asked to take into consideration the

For peer review only

extent to which identified outcomes are ‘changeable’, and could be feasibly impacted by psycho-social interventions. The final COS and also a list of all items reaching consensus will be published.

ETHICS AND DISSEMINATION

Ethical approval

Ethical approval was sought from the Research Ethics committee at University College London. At all stages of the study we will obtain written consent for contact information relating to potential participants to be passed via gatekeeper organisations assisting with recruitment. We will obtain written informed consent from participants in interviews and the consensus meeting. Online consent will be obtained from participants when they opt in to participate in the E-Delphi study, before they are able to proceed.

Dissemination and implementation

We have registered the study on the COMET website. We will provide tailored briefings to UK policy makers, think tanks, commissioners and third sector organisations whilst the study is in progress as well as completed. This will maximise interest and intention to use the core outcome sets. We also intend to use these briefings as a vehicle for recruitment to the E-Delphi study. We will involve the leads of international scholarly networks in workshops and recruit member networks to the E-Delphi study

We will disseminate our findings through peer reviewed and open access publications, the COMET website, and presentations at international conferences. We will engage with journal editors and funding agencies and the relevant Cochrane and Campbell review groups to promote awareness of the CM- and DVA-COS. We will provide briefings and links to publications to international research and policy networks, for dissemination through the networks of the VAMHN membership and CPRU collaborators, as well as the wider network of NIHR Policy Research Units, Applied Research Collaborations (ARCs) and UKRI networks. We will invite survivors who participated in workshops and in involvement groups to co-produce plain-language, service-user facing communication materials for circulation in places where survivors access support (formal or informal). We will also develop tailored briefings to enable findings to be shared with all study participants; participation in this type of study is known to be a key facilitator of implementation.[15] Briefings will be published on the CPRU website and emailed to all third sector organisations working specifically with survivors of CM and DVA, as well as Local Authority commissioners and CCGs.

For peer review only

For peer review only - http://bmjopen.bmj.com/site/about/guidelines.xhtml

A high level review of the reach and uptake of the core outcome sets will be undertaken in 2023. One of the key issues for review will be whether the core outcome set has become aligned or adopted by research and practice networks or collaborations ,and recognised by funders (e.g. NIHR) and bodies co-ordinating health and social care intervention research and systematic reviews (e.g. Cochrane and Campbell Collaborations)

DISCUSSION

Currently no published COS exists for evaluation of services and interventions to improve child outcomes following experience of CM or DVA. It is essential that outcomes measured in the context of trials and practice based research reflect the benefits (and harms) sought and prioritised by those who use, deliver and commission DVA and CM programmes, as well as those who research them. A COS that is developed with strong participation from people with lived experience of CM or DVA and those working to support them will help to ensure that relevant outcomes are measured in all evaluative studies. This in turn will enhance consistency across studies and the quality and value of research. High levels of awareness and uptake of this study's outputs is critical to achieving its ultimate aim.

Limitations

The design of this study is limited by the lack of direct involvement of children and young people in either qualitative interviews or the E-Delphi study. Given the study described here represents meta-research, it was felt that potential risks to children could not be justified. Their voices are nonetheless to some extent reflected through the broad reviews of evidence and inclusion of parent perspectives. It is also beyond the means of the study to involve survivors and service providers from LMICs, although we will include research from LMICs in the evidence reviews and actively recruit researchers from or researching LMIC settings.

For peer review only - http://bmjopen.bmj.com/site/about/guidelines.xhtml

REFERENCES

1 Evans SE, Davies C, DiLillo D. Exposure to domestic violence: A meta-analysis of child and adolescent outcomes. *Aggress Violent Behav* 2008;**13**:131–40.

2 Gilbert R, Widom CS, Browne K, *et al.* Burden and consequences of child maltreatment in high-income countries. Lancet. 2009;**373**:68–81. doi:10.1016/S0140-6736(08)61706-7

3 MacMillan HL, Wathen CN, Barlow J, *et al.* Interventions to prevent child maltreatment and associated impairment. *Lancet* 2009;**373**:250–66. doi:10.1016/S0140-6736(08)61708-0

4 McTavish JR, Kimber M, Devries K, *et al.* Children's and caregivers' perspectives about mandatory reporting of child maltreatment: a meta-synthesis of qualitative studies. *BMJ Open* 2019;**9**:e025741. doi:10.1136/bmjopen-2018-025741

5 Lewis N V, Feder GS, Howarth E, *et al.* Identification and initial response to children's exposure to intimate partner violence: a qualitative synthesis of the perspectives of children, mothers and professionals. *BMJ Open* 2018;**8**:e019761. doi:10.1136/bmjopen-2017-019761

For peer review only - http://bmjopen.bmj.com/site/about/guidelines.xhtml

6 Home Office. Domestic Abuse Bill 2020: overarching factsheet. London: 2020. https://www.gov.uk/government/publications/domestic-abuse-bill-2020-factsheets/domestic-abuse-bill-2020-overarching-factsheet

7 Howarth E, Moore THM, Welton NJ, *et al.* Improving outcomes for children exposed to domestic violence (IMPROVE): An evidence synthesis. *Public Heal Res* 2016.

8 Macdonald G, Livingstone N, Hanratty J, *et al.* The effectiveness, acceptability and cost-effectiveness of psychosocial interventions for maltreated children and adolescents: An evidence synthesis. *Health Technol Assess (Rockv)* 2016;**20**. doi:10.3310/hta20690

9 Latzman NE, Casanueva C, Brinton J, *et al.* The promotion of well-being among children exposed to intimate partner violence: A systematic review of interventions. *Campbell Syst Rev* 2019;**15**. doi:10.1002/cl2.1049

10 Clarke M. Standardising outcomes for clinical trials and systematic reviews. *Trials* 2007;**8**:1–3. doi:10.1186/1745-6215-8-39

11 Clarke M, Williamson PR. Core outcome sets and systematic reviews. *Syst Rev* 2016;**5**:11. doi:10.1186/s13643-016-0188-6

12 Howarth E, Moore THM, Shaw ARG, *et al.* The Effectiveness of Targeted Interventions for Children Exposed to Domestic Violence: Measuring Success in Ways that Matter to Children, Parents and Professionals. *Child Abus Rev* 2015;**24**:297–310. doi:10.1002/car.2408

13 Hoagwood KE, Jensen PS, Acri MC, *et al.* Outcome domains in child mental health research since 1996: have they changed and why does it matter? *J Am Acad Child Adolesc Psychiatry* 2012;**51**:1241-1260.e2. doi:10.1016/j.jaac.2012.09.004

14 Howarth E, Moore THM, Shaw ARG, *et al.* The Effectiveness of Targeted Interventions for Children Exposed to Domestic Violence: Measuring Success in Ways that Matter to Children, Parents and Professionals. *Child Abus Rev* 2015;**24**:297–310. doi:10.1002/car.2408

15 Williamson PR, Altman DG, Bagley H, *et al.* The COMET Handbook: version 1.0. *Trials* 2017;**18**:280. doi:10.1186/s13063-017-1978-4

16 Davis K, Gorst SL, Harman N, *et al.* Choosing important health outcomes for comparative effectiveness research: An updated systematic review and involvement of low and middle income countries. *PLoS One* 2018;**13**. doi:10.1371/journal.pone.0190695

17 Gorst SL, Gargon E, Clarke M, *et al.* Choosing important health outcomes for comparative

For peer review only

For peer review only - http://bmjopen.bmj.com/site/about/guidelines.xhtml

effectiveness research: An updated review and user survey. *PLoS One* 2016;**11**. doi:10.1371/journal.pone.0146444

18 Hamby S, Finkelhor D, Turner H, *et al.* The overlap of witnessing partner violence with child maltreatment and other victimizations in a nationally representative survey of youth. *Child Abuse Negl* 2010;**34**:734–41. doi:http://dx.doi.org/10.1016/j.chiabu.2010.03.001

19 Henry C. Exposure to domestic violence as abuse and neglect: Constructions of child maltreatment in daily practice. *Child Abus Negl* 2018;**86**:79–88. doi:10.1016/j.chiabu.2018.08.018

20 Lawson J. Domestic violence as child maltreatment: Differential risks and outcomes among cases referred to child welfare agencies for domestic violence exposure. *Child Youth Serv Rev* 2019;**98**:32–41. doi:10.1016/j.childyouth.2018.12.017

21 Kirkham JJ, Gorst S, Altman DG, *et al.* Core Outcome Set-STAndardised Protocol Items: the COS-STAP Statement. *Trials* 2019;**20**:116. doi:10.1186/s13063-019-3230-x

22 Woodman J, Simon A, Hauari H, *et al.* A scoping review of 'think-family' approaches in healthcare settings. *J Public Health (Bangkok)* Published Online First: 22 January 2019. doi:10.1093/pubmed/fdy210

23 England MJ, Butler AS, Gonzalez ML, *et al. Psychosocial interventions for mental and substance use disorders : a framework for establishing evidence-based standards*.

24 Leamy M, Bird V, Le Boutillier C, *et al.* Conceptual framework for personal recovery in mental health: Systematic review and narrative synthesis. Br. J. Psychiatry. 2011;**199**:445–52. doi:10.1192/bjp.bp.110.083733

25 Jorm AF. Using the Delphi expert consensus method in mental health research. *Aust New Zeal J Psychiatry* 2015;**49**:887–97. doi:10.1177/0004867415600891

26 Gargon E, Gurung B, Medley N, *et al.* Choosing important health outcomes for comparative effectiveness research: A systematic review. *PLoS One* 2014;**9**. doi:10.1371/journal.pone.0099111

27 Sinha I, Jones L, Smyth RL, *et al.* A systematic review of studies that aim to determine which outcomes to measure in clinical trials in children. *PLoS Med* 2008;**5**:0569–78. doi:10.1371/journal.pmed.0050096

28 Sinha IP, Smyth RL, Williamson PR. Using the Delphi technique to determine which outcomes

For peer review only

For peer review only - http://bmjopen.bmj.com/site/about/guidelines.xhtml

to measure in clinical trials: recommendations for the future based on a systematic review of existing studies. *PLoS Med* 2011;**8**:e1000393. doi:10.1371/journal.pmed.1000393

29 Macefield R, Blencowe N, Brookes S, *et al.* Core outcome set development: the effect of Delphi panel composition and feedback on prioritisation of outcomes. *Trials* 2013;**14**:P77. doi:10.1186/1745-6215-14-S1-P77

30 Thiara RK, Hague G, Mullender A. Losing out on both counts: Disabled women and domestic violence. *Disabil Soc* 2011;**26**:757–71. doi:10.1080/09687599.2011.602867

31 Anitha S. No Recourse, No Support: State Policy and Practice Towards South Asian Women Facing Domestic Violence in the UK. 2010. doi:bcn160

32 Douglas H, Walsh T. Mandatory Reporting of Child Abuse and Marginalised Families. Springer, Dordrecht 2015. 491–509. doi:10.1007/978-94-017-9685-9_23

33 Brophy J. Child maltreatment in diverse households: Challenges to law, theory, and practice. *J Law Soc* 2008;**35**:75–94. doi:10.1111/j.1467-6478.2008.00415.x

34 Ganann R, Ciliska D, Thomas H. Expediting systematic reviews: Methods and implications of rapid reviews. *Implement Sci* 2010;**5**:56. doi:10.1186/1748-5908-5-56

35 Schünemann HJ, Moja L. Reviews: Rapid! Rapid! Rapid!.and systematic. Syst. Rev. 2015;**4**:4. doi:10.1186/2046-4053-4-4

36 Howarth E, Moore THM, Welton NJ, *et al.* Improving outcomes for children exposed to domestic violence (IMPROVE): An evidence synthesis. *Public Heal Res* 2016;**4**. doi:10.3310/phr04100

37 Arai L, Heawood A, Feder G, *et al.* Hope, Agency, and the Lived Experience of Violence: A Qualitative Systematic Review of Children’s Perspectives on Domestic Violence and Abuse. *Trauma, Violence, Abus* 2019;:152483801984958. doi:10.1177/1524838019849582

38 Braun V, Clarke V. Using thematic analysis in psychology. *Qual Res Psychol* 2006;**3**:77–101.

39 Braun V, Clarke V, Boulton E, *et al.* The online survey as a *qualitative* research tool. *Int J Soc Res Methodol* 2020;:1–14. doi:10.1080/13645579.2020.1805550

40 Dodd S, Clarke M, Becker L, *et al.* A taxonomy has been developed for outcomes in medical research to help improve knowledge discovery. *J Clin Epidemiol* 2018;**96**:84–92. doi:10.1016/J.JCLINEPI.2017.12.020

For peer review only

For peer review only - http://bmjopen.bmj.com/site/about/guidelines.xhtml

41 Heise LL. What works to prevent partner violence. *Dfid* 2011;:1–110. doi:978 0 902657 85 2

42 Heise LL. Violence against women: An integrated, ecological framework. *Violence Against Women* 1998;**4**:262–90. doi:10.1177/1077801298004003002

43 Belsky J. Etiology of child maltreatment: A developmental-ecological analysis. *Psychol Bull* 1993;**114**:413–34. doi:10.1037/0033-2909.114.3.413

44 van IJzendoorn MH, Bakermans-Kranenburg MJ, Coughlan B, *et al.* Annual Research Review: Umbrella synthesis of meta-analyses on child maltreatment antecedents and interventions: differential susceptibility perspective on risk and resilience. J. Child Psychol. Psychiatry Allied Discip. 2020;**61**:272–90. doi:10.1111/jcpp.13147

45 Cameranesi M, Piotrowski CC. Critical review of theoretical frameworks elucidating the mechanisms accounting for the adverse developmental outcomes observed in children following exposure to intimate partner violence. Aggress. Violent Behav. 2020;**55**:101455. doi:10.1016/j.avb.2020.101455

46 Blazeby JM, Macefield R, Blencowe NS, *et al.* Core information set for oesophageal cancer surgery. *Br J Surg* 2015;**102**:936–43. doi:10.1002/bjs.9840

47 Gargon E, Crew R, Burnside G, *et al.* Higher number of items associated with significantly lower response rates in COS Delphi surveys. *J Clin Epidemiol* 2019;**108**:110–20. doi:10.1016/j.jclinepi.2018.12.010

48 Sherratt FC, Eaton S, Walker E, *et al.* Development of a core outcome set to determine the overall treatment success of acute uncomplicated appendicitis in children: A study protocol. *BMJ Paediatr Open* 2017;**1**:e000151. doi:10.1136/bmjpo-2017-000151

49 Knaapen M, Hall NJ, Van Der Lee JH, *et al.* Establishing a core outcome set for treatment of uncomplicated appendicitis in children: Study protocol for an international Delphi survey. *BMJ Open* 2019;**9**:e028861. doi:10.1136/bmjopen-2018-028861

LEGEND

Figure 1: Study design

ACKNOWLEDGEMENTS

First and foremost we thank the survivors and other members of the public who contributed to this study. Survivor involvement is facilitated by VOICES, a Survivor led charity for women who have experienced domestic abuse. We also extend our thanks to members of our advisory groups which

For peer review only

have informed the development of the study design and have commented on drafts of this manuscript. Members of the professional advisory group are as follows: Elaine Fulton, Dr Deborah Hodes, Dr Carol Rivas, Professor Sally Kendall, David and Elisabeth Carney Haworth and Hannah Edwards.

AUTHOR CONTRIBUTIONS

EH conceived of the original study design, which was refined and developed by EH, CP, RG, and GF, JW, ES and EW. CP and EW led the development of the public patient involvement strategy. CP and HC developed protocols for rapid reviews, which were reviewed and refined by CP, EH, RG, JW and GF. CP undertook all searches; CP and EH performed data extraction for reviews. EH, CP, RG, JW, EW, ES, and GF contributed to the writing and review of the protocol paper.

FUNDING STATEMENT

This study is funded by the National Institute for Health Research (NIHR) Policy Research Programme. Funder reference: PR-PRU-1217-21301; UCL award code: 177763 The views expressed are those of the author(s) and not necessarily those of the NIHR or the Department of Health and Social Care.

COMPETING INTERESTS

We have read and understood BMJ policy on declaration of interests and declare that we have no competing interests.

DATA SHARING STATEMENT

Please contact the corresponding author or unit manager cpru.data@ucl.ac.uk with enquiries about the data used in this study.

SUPPLEMENTARY MATERIAL

Rapid review protocols

For peer review only

For peer review only - http://bmjopen.bmj.com/site/about/guidelines.xhtml

Figure 1: Study design

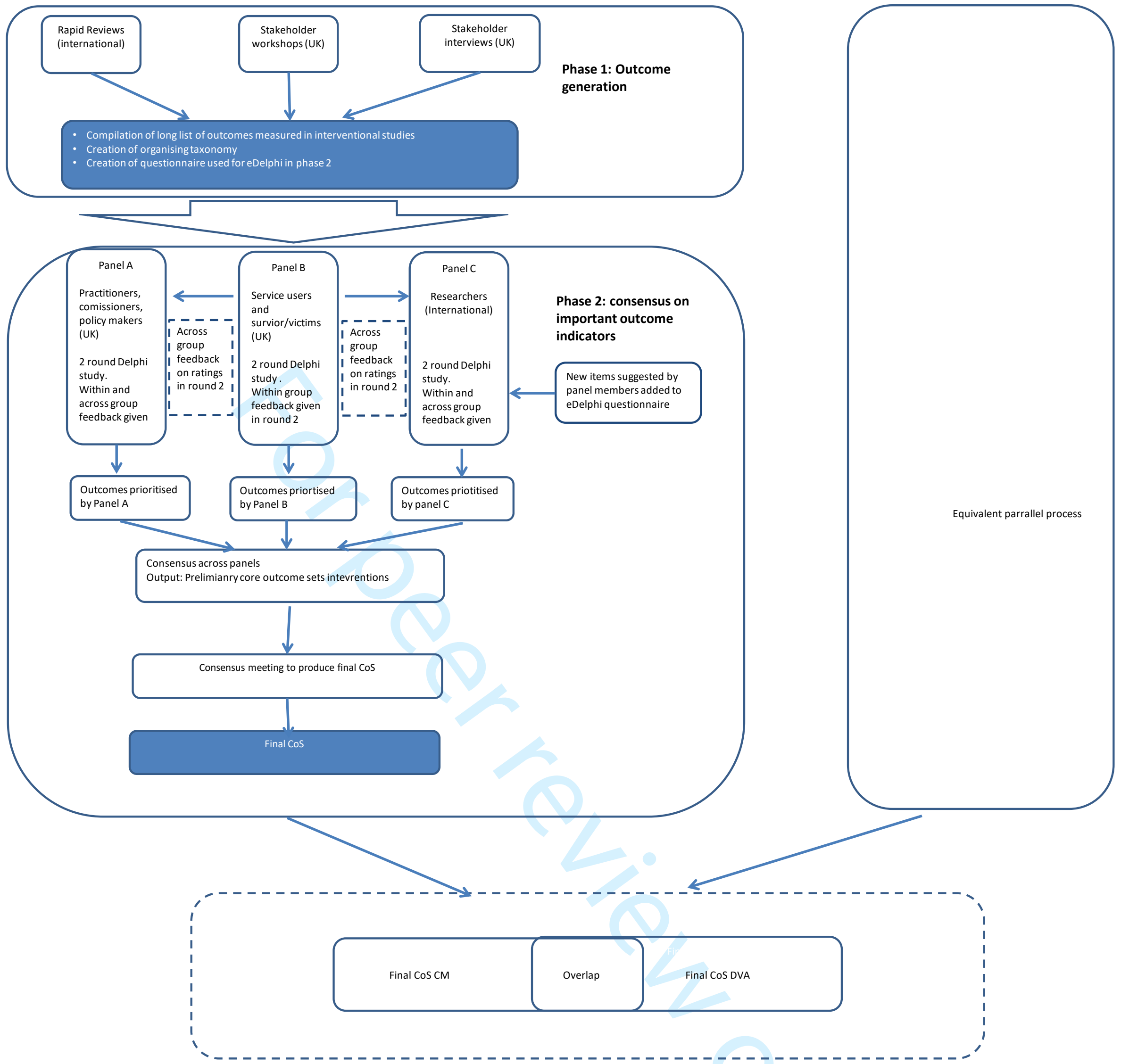

Supplementary Appendices – Rapid Review Protocols

A - Rapid review of systematic reviews of intervention studies

Review question: How are outcomes defined and measured in controlled trials of interventions aiming to improve outcomes of children and families with children exposed to DVA/CM and those aiming to reduce subsequent abusive behaviour by perpetrators of DVA/CM?

a. This includes the definition and measurement of DVA/CM.

This rapid review will be carried out in two steps: firstly searches for systematic reviews (SR) will be carried out, then these reviews will be used to extract individual studies which will be screened for relevance. This process will be carried out in parallel for the DVA and CM literature.

Study inclusion: Peer-reviewed systematic reviews of controlled or quasi experimental comparator intervention studies: with or without randomisation.

The DARE criteria for SRs are at least 4 of the following: reporting of inclusion/exclusion criteria; adequate search; synthesis of included studies; quality assessment of studies; sufficient detail presented (CRD, 1995). For the purposes of this review, SRs will be included if they use an electronic database and have a structured search strategy.

- Published since 2014.
- No restrictions by country. English language only.

- Individual studies must include DVA/CM in one of the following ways:
  - Entry to the intervention is determined by experience, perpetration or identified as at risk of DVA/CM. (Identification of risk is by researchers, practitioners or participants thus we do not have a definition)
  - Subgroup analysis is carried out of participants who have experienced (or are considered to be at risk of) DVA/CM
  - DVA/CM is measured as an exposure (this could be retro or prospectively reported)

Exclusion: Non peer-reviewed studies, qualitative studies, general literature reviews, protocols, case reports, cross-sectional studies, general discussion papers, letters, commentaries, book chapters, conference papers, theses and dissertations.

Population inclusion: children or families with children at risk of experiencing, or experiencing DVA/CM'. This includes unborn children, children (aged 0 to 18 years), designated as victim or witness. For DVA any adult family members who have a parenting role (Early Intervention Foundation, 2014), whether designated as perpetrator, victim, witness or household member.
For CM any adult family members who have a caring role, whether designated as maltreating parent, witness or household member. These adults and children could either be the primary study population of interest or form a subgroup in a wider study population.

Intervention inclusion: Any interventions or services where:

For peer review only - http://bmjopen.bmj.com/site/about/guidelines.xhtml

- Experience of or increased risk of experiencing DVA/CM is a criterion for being offered the service
  OR
- DVA/CM is measured as an exposure or outcome of interest
  AND
- At least one child or family-level outcome is measured. Family-level outcomes do not need to be explicitly labelled as 'family' level, we will make a judgement. However, they include any outcome that affects the family/household unit. For example, worklessness in study where at least some participants are reported to be parents would be included.

- Studies must include evaluation of a defined activity/programme and evaluation of a hypothesised effect
- Interventions may be delivered to any family member(s) as an individual or in a group. Any duration of intervention will be included. Any setting will be considered.

Exclusion: universal interventions that do not specifically target children and families at risk of DVA/CM; targeted interventions that do not measure any child or family level outcomes e.g. perpetrator programmes that focus solely on attitudinal change; DVA (only) interventions focused solely on elder abuse, sibling abuse or child perpetration of domestic violence where participants have not been identified as exposed to DVA.

Comparator inclusion: Any control or comparison group/period with participants receiving no care, treatment as usual or any other treatment.

Outcome inclusion:
- Any child outcome related to i) the child's experience of adversity ii) child functioning, including risky behaviours (see (Maclean et al., 2016) for full list of health and wellbeing outcomes).
- Any outcomes related to the quality of the caregiving environment (e.g. parenting, maternal depression, stressful life events, maternal psychological distress, parental substance misuse).
- Any outcomes related to material deprivation e.g. low income, economic hardship or stress (including perceived), social capital, hunger, food poverty, housing instability.
- Any other outcome judged to relate to children or families by the research team.
- Outcomes can be reported by professionals, child, parent or other family member and they can be retrospective or prospective.
- Outcomes can be end points, surrogate markers for end points or intermediate outcomes.
- No minimum or maximum follow-up is required.

Context inclusion: Studies from any country in any setting.

Searches
The following electronic databases will be searched from 2014: Medline, Embase, PsycInfo, Cochrane and Web of Science. Searching will include expert recommendations of relevant broader studies, including relevant parenting programmes.

The search strategy will include MeSH terms relating to DVA/CM and the BMJ systematic review strategy ((*Study Design Search Filters | BMJ Best Practice*, n.d.)). Key word terms for DVA/CM, abuse,

For peer review only - http://bmjopen.bmj.com/site/about/guidelines.xhtml

violence, family members and systematic reviews will be used. These have been developed from the two main NIHR-funded studies in the area ((Howarth et al., 2016) and (Macdonald et al., 2016)) and adapted as required for the different databases with guidance from an expert librarian.

These reviews will be carried out separately for DVA and CM. The DVA search will be run first and any CM studies that do not mention DVA will be excluded (and vice versa). As part of the review involves collecting definitions of DVA/CM, any study deemed to fit within the umbrella by the research team will be included.

Data extraction (selection and coding)

All systematic reviews identified by database searches will be downloaded to CADIMA (Kohl et al., 2018) and de-duplicated. Screening criteria will be tested by two reviewers on 200 titles/abstracts and interrater reliability assessed. Titles/abstracts will be screened by one reviewer for inclusion in full-text review. A second reviewer will independently review 10% of title/abstracts. If there is a high level of disagreement, the second reviewer will continue reviewing titles/abstracts until agreement is reached. Full-text systematic reviews will be screened for inclusion and a second reviewer will independently review 10% of these as above. Key data from the systematic reviews (e.g. definition of DVA/CM) will be extracted into CADIMA by one reviewer.

Individual studies will be extracted from the included full-text systematic reviews. These studies will be downloaded to Zotero and de-duplicated. The remaining studies will then be screened for inclusion in full-text review and data extraction. Data will be extracted into Access using a standardised form and a second researcher will review extraction from the first 5 papers. The following data will be extracted: bibliographic information, study design, setting, sample characteristics, definitions of DVA/CM, intervention details, primary and secondary outcomes (applicable for children and families) and their measures, descriptions of mechanisms. (Where DVA/CM is not measured as an outcome, nor is there a subgroup analysis, only exposure definition will be extracted.) Quality control/risk of bias will not be assessed because the aim of the review is solely to collect outcomes.

Strategy for data synthesis

Narrative synthesis and tabulation of outcomes extracted.

B - Rapid review of qualitative studies

Review questions:

1) What outcomes (benefits or harms) are sought or experienced by actual or potential recipients of interventions/services aiming to prevent or reduce the risk of harm associated with DVA/CM?

2) What outcomes (benefits or harms) are sought by stakeholders* involved in developing and/or delivering interventions to children/families experiencing DVA/CM?

**'stakeholder' is defined as in the IMPROVE study i.e. young people with experience of DVA/CM services, parents/caregivers with experience of using DVA/CM services or professionals involved in commissioning and delivering services to families affected by DVA/CM.*

For peer review only - http://bmjopen.bmj.com/site/about/guidelines.xhtml

This review will be carried out in parallel for DVA and CM.

Study inclusion:

- Primary qualitative (i.e. analysis of interviews, focus groups or other verbal analysis which is not quantified) intervention studies either as a standalone study or a discrete component of mixed-method studies.
- Direct and sufficient verbatim text from participants for analysis (i.e. more than two lines) c.f. Arai et al. (2019).
- Published since October 2015 (DVA) and July 2014 (CM) to build on Howarth et al. (2016) and Macdonald et al. (2016).
- No restrictions by country. English language only.

- Individual studies must include DVA/CM in one of the following ways:
    - Participation in the study is determined by experience, perpetration or specifically identified as at risk of DVA/CM. Participants may have received an intervention or may be discussing the impact of DVA/CM and their desired outcomes for the future. (To ensure we are not limited by outcomes defined by current interventions).

    OR

    - Stakeholders involved in developing and/or delivering interventions to children/families experiencing DVA/CM (c.f. Howarth et al, 2016, p.52), or stakeholder discussion of outcomes that are sought either in relation to an intervention or the future in general.

Exclusion: Non peer-reviewed studies, surveys or quantitative studies with descriptive free-text only, general literature reviews, case reports, general discussion papers, letters, commentaries, editorials, book chapters, conference papers, theses and dissertations.

Population inclusion: Any adult or child stakeholders relevant to DVA/CM. This could be as a result of experience, perpetration, identified as at risk, delivering, commissioning or intending to deliver services.

Phenomenon of interest: DVA/CM

Design: Any qualitative approach to data collection and analysis (e.g. interviews, focus groups)

Evaluation: Perspectives of experienced or anticipated benefits or harms of interventions, and/or desired outcomes in general related to DVA/CM.

Searches

The following electronic databases as advised for qualitative research (Evans, 2002; McFadden et al, 2012; Booth, 2016) will be searched from October 2015 (DVA) and July 2014 (CM): ASSIA, CINAHL, GoogleScholar (first 100 hits), PsycInfo and SSCI.

This review is building on Howarth et al. (2016) and Macdonald et al. (2016) so relevant studies from these reviews (and related work such as Arai et al. (2019)) will be included. In addition, expert recommendations of relevant qualitative studies or reviews and any qualitative studies identified from the reviews of systematic reviews will be included.

For peer review only - http://bmjopen.bmj.com/site/about/guidelines.xhtml

The search strategy will use the same terms for DVA/CM as the review of systematic reviews, plus additional search terms to identify qualitative research. These will be adapted as required for the different databases with guidance from an expert librarian.
These reviews will be carried out separately for DVA and CM. The DVA search will be run first and any CM studies that do not mention DVA will be excluded (and vice versa) but put aside for inclusion in the relevant review. This review will not adhere to set definitions of DVA/CM, thus any study deemed by the research team to address the phenomena of interest will be included and justified in the discussion of findings.

Screening
Screening of abstracts from the searches and articles included in the full text stage will be guided by questions asked in the IMPROVE study (Howarth et al., 2016):

1) Is this qualitative research?
2) Is there sufficient verbatim text? (i.e. more than 2 lines)
3) Does the paper discuss perspectives of experienced or anticipated benefits or harms of interventions, and/or desired outcomes in general related to DVA/CM.

All articles identified by searches will be downloaded to CADIMA (Kohl et al., 2018) and de-duplicated. Screening criteria will be tested by two reviewers on 10% titles/abstracts and interrater reliability assessed. Titles/abstracts will be screened by one reviewer for inclusion in full-text review. A second reviewer will independently review 10% of title/abstracts. If there is a high level of disagreement, the second reviewer will continue reviewing titles/abstracts until agreement is reached. Full-text systematic reviews will be screened for inclusion and a second reviewer will independently review 10% of these as above. Key details (e.g. bibliographic information, study design, setting, participants etc.) about each full-text inclusion will be recorded in Access.

Strategy for data synthesis
Thematic frameworks will be developed from the IMPROVE study (Howarth et al., 2016) for DVA and the parallel CM study (MacDonald et al., 2016), and input into NVivo 11 (QSR International). The frameworks will focus on barriers and harms of interventions according to parents, children and stakeholders, based on the research questions. These will be used as the basis for a framework analysis (Ritchie & Lewis, 2003) of the studies from the review (Howarth et al., 2016; Arai et al., 2019; Macdonald et al., 2016). As per Howarth et al. (2016), participant quotations and author-identified themes will be extracted rather than line by line coding. Findings will be grouped by whose view was reported and extracts from the texts will be categorised according to this framework with the aim will be to meta-aggregate the studies' findings. Further categories will be developed where there are discrepancies or gaps in the initial framework.

The analysis and interpretation of the findings will occur at the synthesis stage in order to provide an overview of the findings, informed by the principles of meta-synthesis (c.f. Noblit & Hare, 1988), although using a lighter touch given time constraints. Two researchers will work together throughout this process to ensure consistency of categorisation and analysis. Quality will not be assessed because the aim of the review is solely to identify candidate outcomes. The ENTREQ statement (Tong et al., 2012) will be followed for the write-up.

For peer review only - http://bmjopen.bmj.com/site/about/guidelines.xhtml

C – Rapid review of grey literature

Review questions:

1) How are DVA and CM defined in relevant UK service policy contexts?
2) How are outcomes defined: (i) in UK service-based evaluations of interventions? (ii) in relevant policy or commissioning frameworks?

This review will be carried out as a single process given the likelihood of crossover literature. Findings will be recorded as DVA or CM or both.

Literature inclusion:

- Any national or regional policy or practice document that reports on DVA/CM-relevant services or outcomes (e.g. measurement/theory).
- Participation in the service is determined by experience, perpetration or identified as at risk of DVA/CM. (Identification of risk is by practitioners or participants thus we do not have a definition).
- Published since 2016 to build on Howarth et al. (2016) and Macdonald et al. (2016).
- England-based only. English language only.

Exclusion: Publication in academic journals, book chapters, conference papers, theses and dissertations.

Population inclusion: children or families with children at risk of experiencing, or experiencing DVA/CM. This includes unborn children, children (aged 0 to 18 years), designated as victim or witness. For DVA any adult family members who have a caring or parenting role (Early Intervention Foundation, 2014), whether designated as perpetrator, victim, witness or household member. For CM any adult family members who have a caring role, whether designated as perpetrator, witness or household member.

Service inclusion: Any services where:

- Experience of or increased risk of experiencing DVA/CM is a criterion for being offered the service/intervention.
- Services/interventions may be delivered to any family member(s) as an individual or in a group. Any duration of service/intervention will be included. Any setting will be considered.

OR

- Any evaluative work or outcomes framework where at least one child or family-level outcome is evaluated/discussed. Family-level outcomes do not need to be explicitly labelled as 'family' level, we will make a judgement. However, they include any outcome that affects the family/household unit. For example, worklessness in study where at least some participants are reported to be parents would be included.

Exclusion: universal services/interventions that do not specifically target children and families at risk of DVA/CM; targeted services/interventions that do not measure any child or family level outcomes e.g. perpetrator programmes that focus solely on attitudinal change; DVA (only) services/interventions focused solely on elder abuse, sibling abuse or child perpetration of domestic violence, where participants have not been identified as exposed to DVA (i.e. perpetration of abuse by a child could feasibly be an outcome associated with exposure).

For peer review only - http://bmjopen.bmj.com/site/about/guidelines.xhtml

Outcome inclusion: Any family or child-level outcome measured or evaluated or discussed in any way. Intermediate outcomes that could feasibly represent preconditions needed to reach distal/final outcomes (including those relating to the process of service delivery) will be included, along with final/distal outcomes.

Searches

The following databases and websites will be searched:

Grey databases: NICE Evidence Search and Open Grey

Organisation websites including but not limited to:

DVA: Women's Aid, Refuge, Respect, Safe Lives, Voices, AVA, Standing Together, Imkaan, The Stefanou Foundation, Women's Trust, Hestia, DVIP, Nia, The Haven, ManKind Initiative, Everyman Project, NCDV, Galop, LAWA, IDAS, Advance, Your Sanctuary, Advocacy After Fatal Domestic Abuse (AAFDA); Aurora New Dawn; My Sister's Place

CM: Centre of expertise on child sexual abuse, FDAC, SCIE, The Survivors' Trust

General websites: Victim Support, Barnardos, NSPCC, Early Intervention Foundation, NatCen, RCGP, RCN, RCM, NICE, BPS, IHV, WHO, UNICEF, Working together, gov.uk (incls e.g. DA bill, 'Working together'), Public Health for any UK nation, Office of the children's commissioner for any UK nation, Big Lottery, Comic Relief, The Childhood Trust, UK College of Policing, Research in Practice, 'What Works', Joseph Rowntree Foundation, What Works for Children's Social Care.

Websites will be searched manually for relevant documents. It is anticipated there will be an element of snowball searching as relevant organisations will have links to further organisations. Searches will be run simultaneously and then relevant reports assigned to DVA/CM or both. All websites searched will be recorded in Excel/Access along with relevant details about any reports captured. The expert reference group will be consulted about relevant websites to search or reports to include at multiple timepoints.

Data extraction and synthesis

As a range of types of data are anticipated, both the systematic review and the qualitative review protocols will be adapted as necessary to capture and record relevant information. It is likely that there will be non-standardised evaluation measures and interview quotations. Report identification from websites/databases will be carried out by a single researcher and the process transparently recorded. All details regarding evaluation studies and relevant outcomes will be recorded, and where necessary synthesised when the data is qualitative. Access/Excel/NVivo will be used as required to record all steps and ensure a transparent process. A second researcher will cross-check a subset of the reports and the data extracted to ensure consistency and focus on the review questions.

For peer review only - http://bmjopen.bmj.com/site/about/guidelines.xhtml

Bibliography

Arai, L., Heawood, A., Feder, G., Howarth, E., MacMillan, H., Moore, T. H. M., Stanley, N., & Gregory, A. (2019). Hope, Agency, and the Lived Experience of Violence: A Qualitative Systematic Review of Children's Perspectives on Domestic Violence and Abuse. *Trauma, Violence, & Abuse*, 152483801984958. https://doi.org/10.1177/1524838019849582

CRD. (1995). *Database of Abstracts of Reviews of Effects (DARE): Quality-assessed Reviews*. Centre for Reviews and Dissemination (UK).

Early Intervention Foundation. (2014). *Early Intervention in Domestic Violence and Abuse*.

Howarth, E., Moore, T. H., Welton, N. J., Lewis, N., Stanley, N., MacMillan, H., Shaw, A., Hester, M., Bryden, P., & Feder, G. (2016). IMPRoving Outcomes for children exposed to domestic ViolencE (IMPROVE): An evidence synthesis. *Public Health Research*, *4*(10), 1–342. https://doi.org/10.3310/phr04100

Kohl, C., McIntosh, E. J., Unger, S., Haddaway, N. R., Kecke, S., Schiemann, J., & Wilhelm, R. (2018). Online tools supporting the conduct and reporting of systematic reviews and systematic maps: A case study on CADIMA and review of existing tools. *Environmental Evidence*, *7*(1), 8. https://doi.org/10.1186/s13750-018-0115-5

Macdonald, G., Livingstone, N., Hanratty, J., McCartan, C., Cotmore, R., Cary, M., Glaser, D., Byford, S., Welton, N. J., Bosqui, T., Bowes, L., Audrey, S., Mezey, G., Fisher, H. L., Riches, W., & Churchill, R. (2016). The effectiveness, acceptability and cost-effectiveness of psychosocial interventions for maltreated children and adolescents: An evidence synthesis. *Health Technology Assessment*, *20*(69), 1–508. https://doi.org/10.3310/hta20690

Maclean, M. J., Sims, S., O'Donnell, M., & Gilbert, R. (2016). Out-of-Home Care versus In-home Care for Children Who Have Been Maltreated: A Systematic Review of Health and Wellbeing Outcomes: Systematic Review of Outcomes of Out-of-Home Care. *Child Abuse Review*, *25*(4), 251–272. https://doi.org/10.1002/car.2437

Study design search filters | BMJ Best Practice. (n.d.). Retrieved 16 April 2019, from https://bestpractice.bmj.com/info/us/toolkit/learn-ebm/study-design-search-filters/

Tong, A., Sainsbury, P., & Craig, J. (2007). Consolidated criteria for reporting qualitative research (COREQ): A 32-item checklist for interviews and focus groups. *International Journal for Quality in Health Care*, *19*(6), 349–357. https://doi.org/10.1093/intqhc/mzm042

For peer review only
